# Copper-mediated synthesis of *N*-alkenyl-α,β-unsaturated nitrones and their conversion to tri- and tetrasubstituted pyridines

**DOI:** 10.3762/bjoc.11.226

**Published:** 2015-11-04

**Authors:** Dimitra Kontokosta, Daniel S Mueller, Dong-Liang Mo, Wiktoria H Pace, Rachel A Simpson, Laura L Anderson

**Affiliations:** 1 Department of Chemistry, University of Illinois at Chicago, 845 W. Taylor St., Chicago, IL 60607, U.S.A

**Keywords:** Chan–Lam, copper, nitrone, oxygen transfer, pyridine

## Abstract

A Chan–Lam reaction has been used to prepare *N*-alkenyl-α,β-unsaturated nitrones, which undergo a subsequent thermal rearrangement to the corresponding tri- and tetrasubstituted pyridines. The optimization and scope of these transformations is discussed. Initial mechanistic experiments suggest a reaction pathway involving oxygen transfer followed by cyclization.

## Introduction

**Scheme 1:**
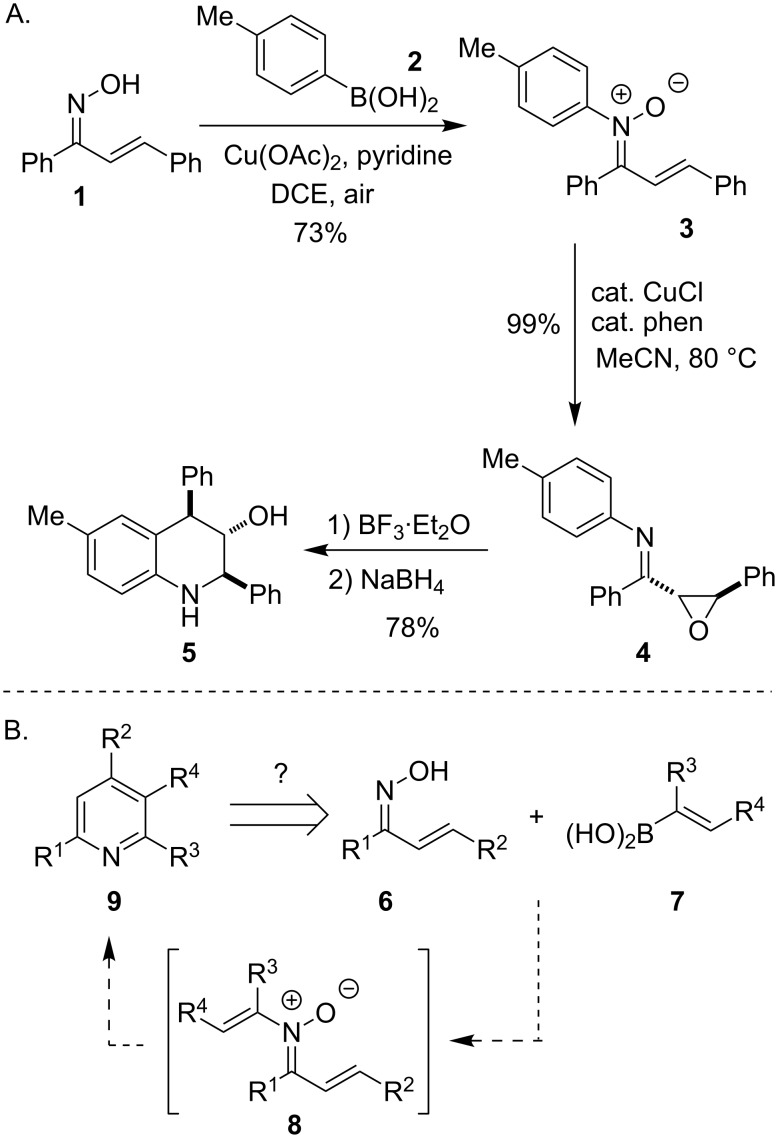
Use of a Chan–Lam reaction for the synthesis of tetrahydroquinolines and potential extension to pyridines [8].

While most applications of the Chan–Lam reaction are focused on the synthesis of aryl ethers and aryl amines, our group has been interested in the use of the Chan–Lam reaction for the synthesis of *O*-alkenyl oximes and hydroxylamines, as well as *N*-alkenyl and *N*-arylnitrones [1–5]. We have discovered that when this transformation is performed with oxime and hydroxamic acid substrates, these reactive intermediates can be accessed and subsequently rearrange to a variety of challenging organic fragments and heterocyclic products [6–13]. Specifically, we reported that *N*-arylnitrones **3** can be prepared by a Chan–Lam coupling of **1** and **2** and that these compounds undergo a copper-catalyzed rearrangement to α,β-epoxyimines such as **4** [8]. Reduction of these products in the presence of a Lewis acid gave tetrahydroquinolines such as **5** (Scheme 1A). These studies encouraged us to consider if similar *N*-alkenylnitrones **8** could be accessed by a Chan–Lam coupling and transformed into the corresponding substituted pyridines **9** (Scheme 1B).

**Scheme 2:**
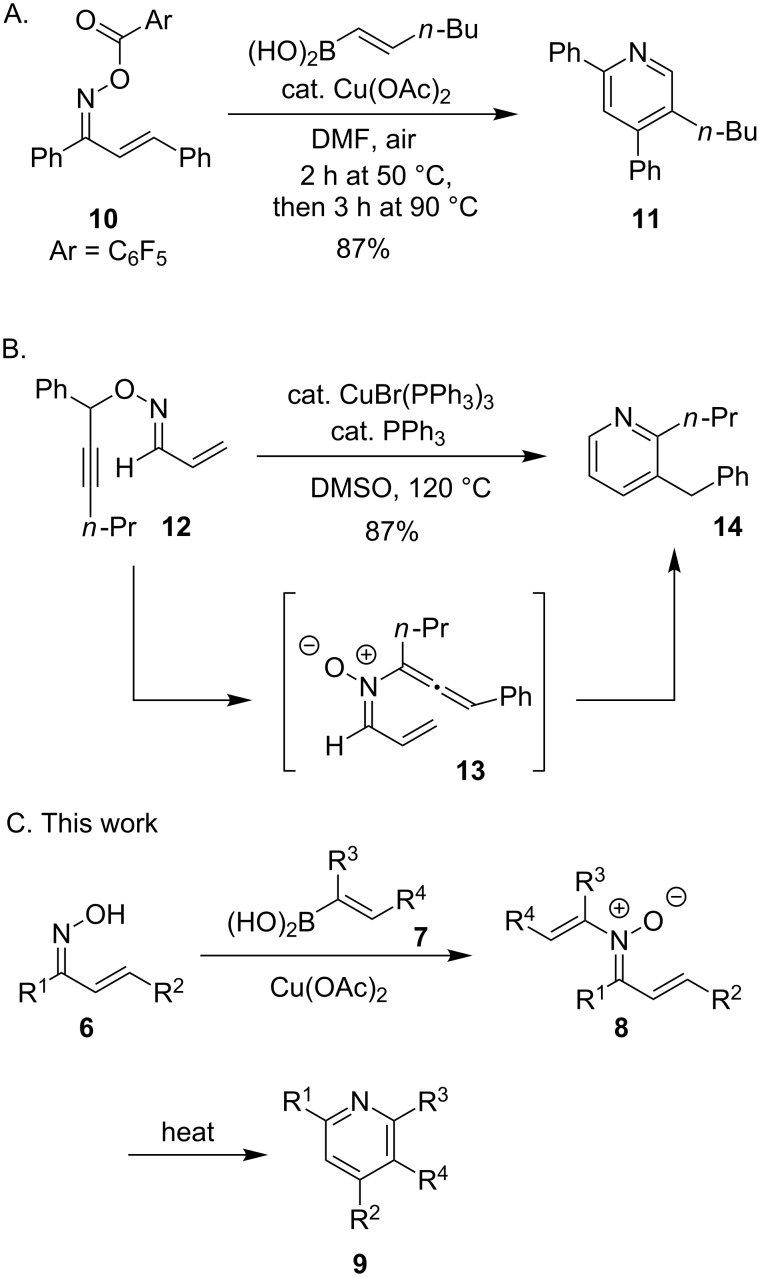
Examples of pyridine synthesis from oxime precursors [51–52].

Pyridines are important heterocycles that are often found in biologically active molecules [14–21]. Due to the high demand for these compounds, there are many methods for preparing substituted pyridines through condensation reactions [22–26], cycloadditions [27–30], functionalization of parent pyridine structures [31–38], fragment couplings [39–42], and transition metal-catalyzed C–H bond functionalization of α,β-unsaturated imines and oximes [43–50]. We were inspired by the copper-catalyzed coupling of protected α,β-unsaturated oximes and alkenylboronic acids developed by Liebeskind and coworkers due to its modularity and control of regioselectivity and wondered if a Chan–Lam route to *N*-alkenylnitrones would allow us to prepare similar intermediates (Scheme 2A) [51]. Nakamura and coworkers have reported that *N*-allenylnitrones can be accessed through rearrangements of *O*-propargylic oximes and undergo similar electrocyclizations to form pyridines (Scheme 2B) [52]. Herein, we show that *N*-alkenylnitrones **8** can be prepared through a Chan–Lam coupling of α,β-unsaturated oximes **6** and an alkenylboronic acids **7** and that these compounds undergo a novel thermal rearrangement to the corresponding tri- and tetrasubstituted pyridines **9** (Scheme 2C). This use of α,β-unsaturated oxime reagents for the synthesis of pyridines is unique from transition metal-catalyzed C–H bond functionalization processes that require a regioselective migratory insertion. This route is appealing due to the modularity of the Chan–Lam coupling process, and proceeds through a pathway that is distinct from the Liebeskind copper-catalyzed C–N bond coupling and electrocyclization (Scheme 2).

## Results and Discussion

A Chan–Lam coupling between chalcone oxime **6a** and cyclohexenylboronic acid (**7a**) was initially tested using reaction conditions that we had previously identified as optimal for analogous *N*-alkenylnitrone syntheses from fluorenone oxime [7]. Nitrone **8aa** was successfully isolated in 40% yield using 2 equiv of Cu(OAc)_2_ and the reaction conditions indicated in Table 1, entry 1. Only the *E*-nitrone isomer was observed and isolated. Decreasing the amount of copper reagent to 1 equiv had little influence on the reaction and a screen of other common copper salts only resulted in diminished yields (Table 1, entries 2–7). Further reduction of the copper loading to 10–30 mol % of Cu(OAc)_2_ was tolerated without a decrease in reaction efficiency (Table 1, entries 8 and 9). The key factor in improving the yield of the transformation was identified as an alkene additive. Alkene and alkyne additives have previously been observed to improve similar copper-catalyzed coupling reactions [53]. As shown in Table 1, entries 10–14, the addition of 1.2 equiv of cyclooctadiene (COD), cyclooctene (COE), norbornadiene (NBD), 1-octene, and dibenzylideneacetone (dba) all improved the yield of the Chan–Lam reaction, but COD was most efficient.

**Table 1:** Optimization of Chan–Lam coupling for the synthesis of *N*-cyclohexenyl-α,β-unsaturated nitrones.^a^

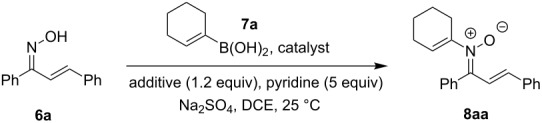				

entry	catalyst	cat. conc	additive	yield (%)^b^

1	Cu(OAc)_2_	2 equiv	none	40
2	Cu(OAc)_2_	1 equiv	none	46
3	CuTC	1 equiv	none	30
4	CuBr	1 equiv	none	dec
5	CuI	1 equiv	none	trace
6	CuOTf_2_	1 equiv	none	trace
7	Cu(TFA)_2_	1 equiv	none	trace
8	Cu(OAc)_2_	10 mol %	none	45
9	Cu(OAc)_2_	30 mol %	none	45
10	Cu(OAc)_2_	10 mol %	COE	65
11	Cu(OAc)_2_	10 mol %	NBD	58
12	Cu(OAc)_2_	10 mol %	COD	72
13	Cu(OAc)_2_	10 mol %	1-octene	57
14	Cu(OAc)_2_	10 mol %	dba	51

^a^Conditions: **6a** (1 equiv), **7a** (2 equiv), pyridine (5 equiv), Na_2_SO_4_ (8–9 equiv), 0.1 M in DCE, 25 °C, air, 18 h. ^b^Determined by ^1^H NMR spectroscopy using CH_2_Br_2_ as a reference.

Having identified optimal conditions for the synthesis of *N*-cyclohexenylnitrone **8aa**, the scope of the nitrone synthesis was explored by varying the oxime and alkenylboronic acid reagents. As shown in Table 2, chalcone oximes with both electron-rich and electron-poor aryl substituents, as well as heteroaryl substituents, were tolerated for the transformation with electron-donating substituents providing higher yields (Table 2, entries 1–4). A significant increase in reaction efficiency was also observed for dba oxime (Table 2, entry 5). Evaluation of acyclic alkenylboronic acids further highlighted the differences between dba oxime and chalcone oximes as substrates for the Chan–Lam reaction. When dba oxime was treated with but-2-en-2-ylboronic acid, nitrone **8eb** was isolated in good yield; in contrast, treatment of chalcone oxime with but-2-en-2-ylboronic acid, resulted in the isolation of nitrone **8ab** in only 15% yield (Table 2, entries 6 and 7). Phenyl-substituted alkenylboronic acid **7c** and monosubstituted alkenylboronic acids **7d** and **7e**, were more efficient reaction partners with chalcone oxime and gave the corresponding nitrones in good to excellent yield (Table 2, entries 8–10). Acyclic alkenylboronic acids were also incompatible with the optimal conditions determined for cyclohexenylboronic acid (**7a**) and required the use of 1–2 equiv of Cu(OAc)_2_. The use of both copper-catalyzed and copper-mediated reaction conditions with oximes **6** and alkenylboronic acids **7**, allowed for the preparation of a variety of *N*-alkenyl-α,β-unsaturated nitrones to test for further reactivity.

**Table 2:** Scope of *N*-alkenyl-α,β-unsaturated nitrone synthesis.^a^

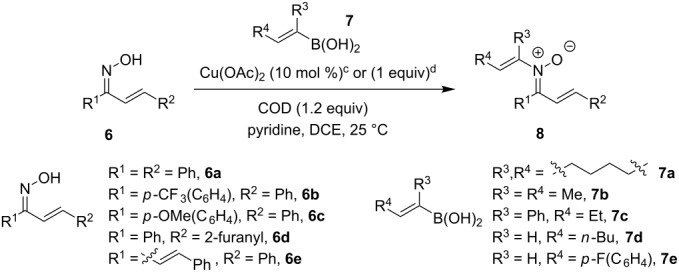					

entry	**8**	yield (%)^b^	entry	**8**	yield (%)^b^

1	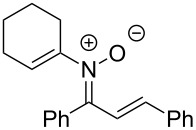 **8aa**	72^c^	6	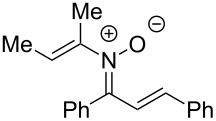 **8ab**	15^d^
2	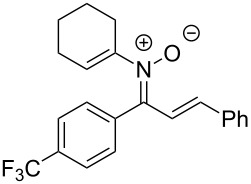 **8ba**	41^c^	7	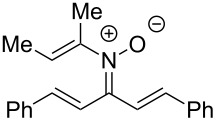 **8eb**	68^d^
3	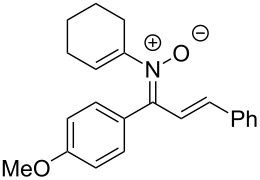 **8ca**	63^c^	8	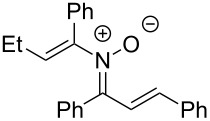 **8ac**	57^d^
4	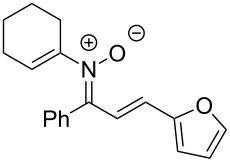 **8da**	70^e^	9	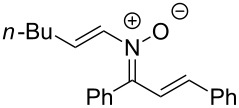 **8ad**	75^d^
5	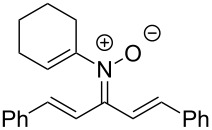 **8ea**	84^c^	10	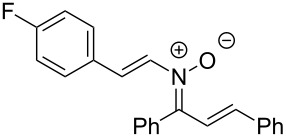 **8ae**	83^d^

^a^Conditions: **6** (1 equiv), **7** (2 equiv), pyridine (5 equiv), COD (1.2 equiv), Na_2_SO_4_ (8–9 equiv), 0.1 M in DCE, 25 °C, air, 18 h. ^b^Percent isolated yield. ^c^Cu(OAc)_2_ (10 mol %). ^d^Cu(OAc)_2_ (1–2 equiv), pyridine (10 equiv), no COD. ^e^Cu(OAc)_2_ (10 mol %), no COD.

**Scheme 3:**
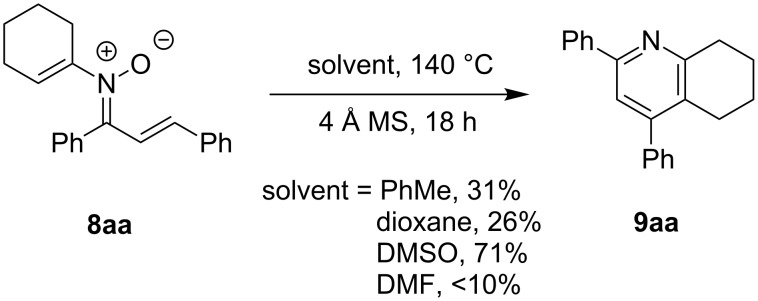
Solvent effect on conversion of *N*-alkenylnitrones to pyridines.

The preparation of the *N*-alkenyl-α,β-unsaturated nitrones shown in Table 2, allowed for further study of their conversion to tri- and tetrasubstituted pyridines. The preliminary evaluation of this thermal transformation with **8aa** indicated that DMSO was a more efficient reaction medium than PhMe, dioxane, or DMF (Scheme 3). As shown in Table 3, all of the chalcone nitrones were readily converted to the corresponding pyridines in good yield (Table 3, entries 1–4, 6, 8–10). In contrast, the dba nitrones gave the corresponding pyridines in attenuated yields (Table 3, entries 5 and 7). The high density of substituents and regiospecificity of the transformation due to the use of the Chan–Lam reaction for the synthesis of the nitrone precursor are noteworthy and provide advantages over pyridine syntheses that require regioselective insertion reactions or nucleophilic additions.

**Table 3:** Scope of conversion of *N*-alkenyl-α,β-unsaturated nitrones to pyridines.^a^

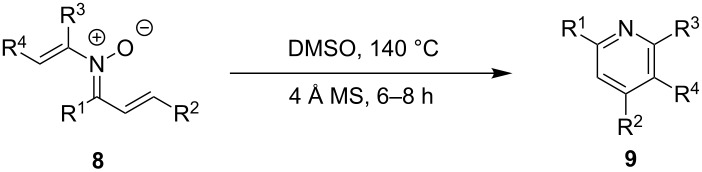					

entry	**9**	yield (%)^b^	entry	**9**	yield (%)^b^

1	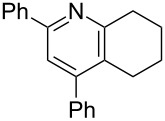 **9aa**	71	6	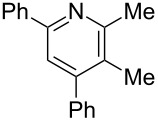 **9ab**	50
2	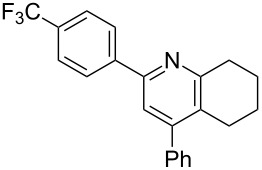 **9ba**	68	7	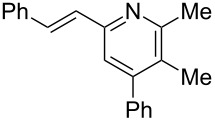 **9eb**	36
3	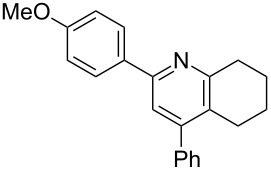 **9ca**	64	8	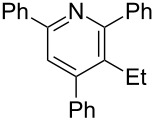 **9ac**	87
4	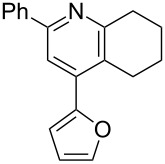 **9da**	76	9	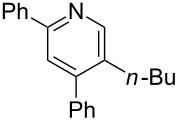 **9ad**	71
5	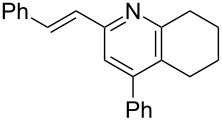 **9ea**	43	10	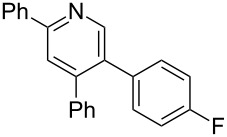 **9ae**	65

^a^Conditions: **8** (1 equiv), 4 Å MS, 0.1 M in DMSO, 140 °C, 6–8 h. ^b^Percent isolated yield.

**Scheme 4:**
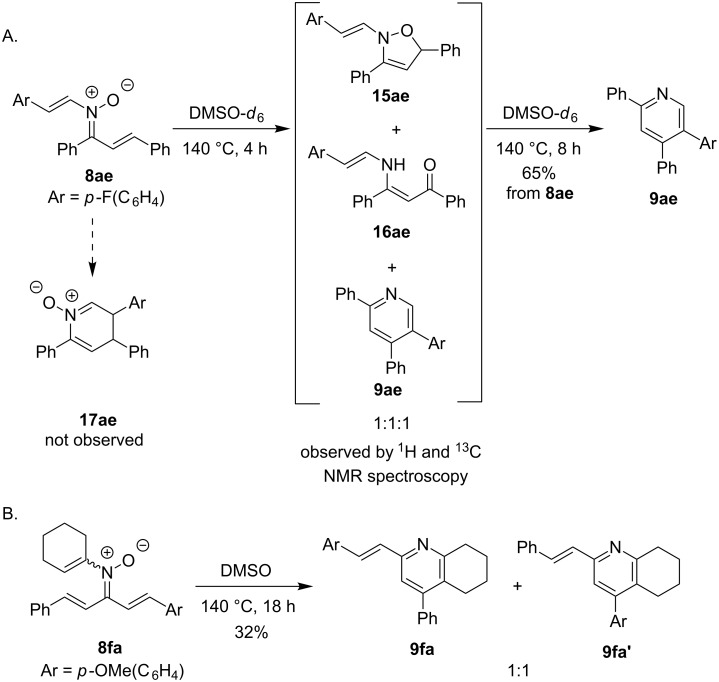
Mechanistic experiments.

To better understand the conversion of *N*-alkenyl-α,β-unsaturated nitrones **8** to pyridines **9**, two mechanistic experiments were evaluated (Scheme 4). The conversion of nitrone **8ae** to pyridine **9ae** was monitored by ^1^H and ^13^C NMR spectroscopy. Surprisingly, after heating **8ae** for 4 h at 140 °C in DMSO-*d*_6_, a 1:1:1 mixture of isoxazoline **15ae**, enaminoketone **16ae**, and pyridine **9ae** was observed [54–55]. Further heating this mixture of intermediates for 4 h resulted in the sole formation of pyridine **9ae**. This experiment suggests that the conversion of nitrone **8ae** to pyridine **9ae** proceeds by oxygen transfer to give **16ae** and nucleophilic addition of the enamine to the ketone. This pathway may explain the solvent dependence that was observed for the transformation (Scheme 3). The lack of any observation of dihydropyridine *N*-oxide intermediate **17ae** suggests that the reaction is not proceeding through an electrocyclization and elimination process. A second experiment tested the electronic effect of this oxygen-transfer process. Unsymmetrically substituted dba nitrone **8fa** was subjected to the cyclization conditions and a 1:1 mixture of **9fa**:**9fa'** was observed. This experiment indicated a lack of any significant electronic preference for the oxygen-transfer process.

## Conclusion

A new method for the preparation of tri- and tetrasubstituted pyridines has been developed that hinges on the use of a Chan–Lam coupling to construct *N*-alkenyl-α,β-unsaturated nitrone precursors from the corresponding oximes and alkenylboronic acids. This method is tolerant of a variety of chalcone- and dba-derived oxime substrates as well as both mono- and disubstituted alkenylboronic acids. Initial reaction monitoring experiments suggest that the cyclization of the *N*-alkenyl-α,β-unsaturated nitrone to the pyridine occurs through an oxygen transfer from the nitrone functionality to the β-position of the conjugated olefin followed by nucleophilic attack of the enamine.

## Supporting Information

File 1 Experimental part.
