# Supplementary material for: Copper-mediated synthesis of N-alkenyl-α,β-unsaturated nitrones and their conversion to tri- and tetrasubstituted pyridines
Source: Beilstein J Org Chem. 2015 Nov 4;11:2097–104. doi: 10.3762/bjoc.11.226 (PMC4660996; doi:10.3762/bjoc.11.226)
Supplement: File 1 — Experimental part. [file Beilstein_J_Org_Chem-11-2097-s001.pdf]

Supporting Information

for

Copper-mediated synthesis of *N*-alkenyl-α,β-unsaturated nitrones and their conversion to tri- and tetrasubstituted pyridines

Dimitra Kontokosta, Daniel S. Mueller, Dong-Liang Mo, Wiktoria H. Pace, Rachel A. Simpson and Laura L. Anderson*

Address: Department of Chemistry, University of Illinois at Chicago, 845 W. Taylor St., Chicago, IL 60607, U.S.A.

Email: Laura L. Anderson - lauralin@uic.edu

*Corresponding author

Experimental part

Contents:

I.	General experimental information	S2
II.	Synthesis of nitrones **8**	S3
III.	Synthesis of pyridines **9**	S10
IV.	References	S15
V.	^{1}H and ^{13}C NMR spectra for nitrones **8**	S16
VI.	^{1}H and ^{13}C NMR spectra for pyridines **9**	S38
VII.	^{1}H and ^{13}C NMR spectra for reaction monitoring	S60

I. General experimental information

^1H NMR and ^{13}C NMR spectra were recorded at ambient temperature using 500 MHz spectrometers. The data are reported as follows: chemical shift in ppm from internal tetramethylsilane on the δ scale, multiplicity (br = broad, s = singlet, d = doublet, t = triplet, q = quartet, m = multiplet), coupling constants (Hz), and integration. High-resolution mass spectra were acquired on an LTQ FT spectrometer, and were obtained by peak matching. Analytical thin-layer chromatography was performed on 0.25 mm extra hard silica gel plates with UV_{254} fluorescent indicator. Medium pressure liquid chromatography was performed to force flow the indicated solvent system down columns packed with 60 Å (40–60 μm) mesh silica gel (SiO_2). Unless otherwise noted, all reagents were obtained from commercial sources and, where appropriate, purified prior to use. Anhydrous 1,2-dichloroethane (DCE) was used as received. Anhydrous dimethyl sulfoxide (DMSO) was used as received.

Oximes **6a**–**e** were reported previously.[1] Alkenylboronic acids **7a**–**d** were reported previously.[2] Boronic acid **7e** was obtained from a commercial source.

II. Synthesis of nitrones

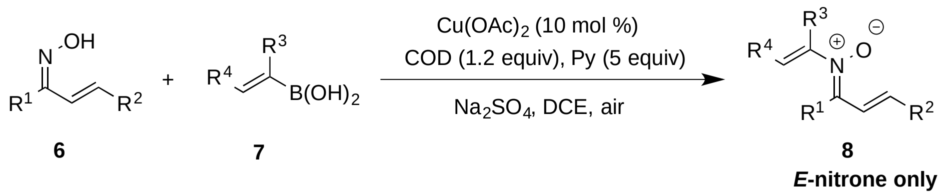

General procedure A: A scintillation vial was charged with oxime **6** (1.0 equiv), alkenyl boronic acid **7** (2 equiv), $Cu(OAc)_2$ (10 mol %), and anhydrous Na_2SO_4 (8–9 equiv). These solids were diluted with DCE to form a 0.1 M solution of oxime. Pyridine (5 equiv) was added to the resulting slurry via syringe, followed by cyclooctadiene (COD) (1.2 equiv). The scintillation vial was then capped with a septum pierced with a ventilation needle and the reaction mixture was stirred at 25 °C for 18 h. DCE and pyridine were removed under reduced pressure and the crude reaction mixture was purified by medium pressure chromatography (2:1; ethyl acetate:hexanes) to give ***E*-8** as a yellow oil or solid. Only the *E*-isomer of the nitrone was observed and isolated.

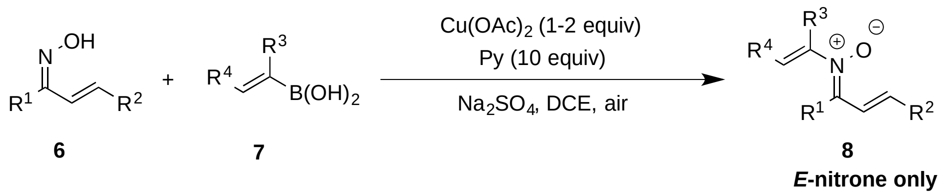

General procedure B: A scintillation vial was charged with oxime **6** (1.0 equiv), alkenyl boronic acid **7** (2-3 equiv), $Cu(OAc)_2$ (1–2 equiv), and anhydrous Na_2SO_4 (8–9 equiv). These solids were diluted with DCE to form a 0.1 M solution of oxime. Pyridine (10 equiv) was added to the resulting slurry via syringe. The scintillation vial was then capped with a septum pierced with a ventilation needle and the reaction mixture was stirred at 25 °C for 18 h. DCE and pyridine were removed under reduced pressure and the crude reaction mixture was purified by medium pressure chromatography (2:1; ethyl acetate:hexanes) to give ***E*-8** as a yellow oil or solid. Only the *E*-isomer of the nitrone was observed and isolated.

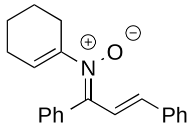

Nitrone 8aa: General procedure **A** was applied using the following reagents: chalcone oxime **6a** (0.030 g, 0.13 mmol), cyclohex-1-enylboronic acid (**7a**, 0.033 g, 0.26 mmol), $Cu(OAc)_2$ (0.0024 g, 0.013 mmol), pyridine (0.052 mL, 0.65 mmol), COD (0.021 mL, 0.16 mmol), and Na_2SO_4 (0.11 g, 0.77 mmol). This procedure afforded ***E*-8aa** as a yellow solid (0.028 g, 72%) after purification using medium pressure chromatography (2:1, EtOAc:hexanes). ^{1}H NMR (500 MHz, $CDCl_3$): δ 8.00 (d, *J* = 16.4 Hz, 1H), 7.48-7.34 (m, 5H), 7.29-7.19 (m, 5H), 6.54 (d, *J* = 16.4 Hz, 1H), 5.56-5.53 (m, 1H), 2.31-2.23 (m, 2H), 1.82-1.80 (m, 2H), 1.57-1.49 (m, 2H), 1.35-1.32 (m, 2H); ^{13}C NMR (125 MHz, $CDCl_3$) δ 148.5, 144.2, 140.3, 136.3, 133.3, 129.8, 129.1, 129.0, 128.6, 128.3, 127.5, 125.8, 121.6, 26.9, 23.9, 21.9, 21.0; IR (thin film) 3056, 3032, 2932, 2858, 2843, 1665, 1603, 1573, 1500, 1467 cm^{-1}; HRMS (ESI) *m/z* calcd. for $C_{21}H_{22}NO$ $(M+H)^+$ 304.1701, found 304.1700.

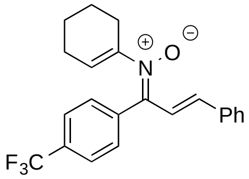

Nitrone 8ba: General procedure **A** was applied using the following reagents: chalcone oxime **6b** (0.030 g, 0.10 mmol), cyclohex-1-enylboronic acid (**7a**, 0.026 g, 0.21 mmol), $Cu(OAc)_2$ (0.0018 g, 0.010 mmol), pyridine (0.042 mL, 0.51 mmol), COD (0.015 mL, 0.12 mmol), and Na_2SO_4 (0.10 g, 0.70 mmol). This procedure afforded ***E*-8ba** as a yellow amorphous solid (0.015 g, 41%) after purification using medium pressure chromatography (2:1, EtOAc:hexanes). ^{1}H NMR (500 MHz, $CDCl_3$): δ 8.00 (d, *J* = 16.5 Hz, 1H), 7.70 (d, *J* = 8.0 Hz, 2H), 7.52-7.45 (m, 2H), 7.42-7.40 (m, 2H), 7.37-7.27 (m, 3H), 6.50 (d, *J* = 16.5 Hz, 1H), 5.57-5.55 (m, 1H), 2.39-2.26 (m, 2H), 1.96-1.78 (m, 2H), 1.65-1.54 (m, 2H), 1.48-1.31 (m, 2H); ^{13}C NMR (125 MHz, $CDCl_3$) δ 146.8, 144.3, 140.0, 137.2, 136.1, 130.5, 130.3, 129.3 (q, $J_{C\text{-}F}$ = 225.0 Hz), 128.9, 128.8, 127.5, 126.4, 125.5, 125.4, 27.0, 23.9, 21.9, 21.0; IR (thin film) 3056, 2936, 1617, 1468, 1322, 1269, 1230, 1165, 1123, 1067 cm^{-1}; HRMS (ESI) *m/z* calcd. for $C_{22}H_{21}F_3NO$ $(M+H)^+$ 372.1575, found 372.1585.

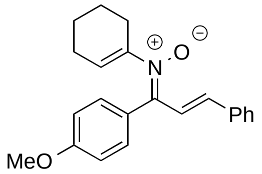

Nitrone 8ca: General procedure **A** was applied using the following reagents: chalcone oxime **6c** (0.040 g, 0.16 mmol), cyclohex-1-enylboronic acid (**7a**, 0.040 g, 0.32 mmol), $Cu(OAc)_2$ (0.0029 g, 0.016 mmol), pyridine (0.065 mL, 0.80 mmol), COD (0.024 mL, 0.19 mmol), and Na_2SO_4 (0.11 g, 0.77 mmol). This procedure afforded ***E*-8ca** as a yellow amorphous solid (0.033 g, 63%) after purification using medium pressure chromatography (2:1, EtOAc:hexanes). ^{1}H NMR (500 MHz, $CDCl_3$): δ 8.05 (d, *J* = 16.4 Hz, 1H), 7.51 (d, *J* = 7.5 Hz, 2H), 7.37-7.29 (m, 3H), 7.21 (d, *J* = 8.3 Hz, 2H), 6.97 (d, *J* = 8.3 Hz, 2H), 6.61 (d, *J* = 16.4 Hz, 1H), 5.66-5.64 (m, 1H), 2.40-2.20 (m, 2H), 1.95-1.92 (m, 2H), 1.74-1.54 (m, 2H), 1.52-1.38 (m, 2H); ^{13}C NMR (125 MHz, $CDCl_3$) δ 159.9, 148.0, 144.6, 140.1, 136.4, 131.2, 129.0, 128.7, 127.5, 125.6, 125.4, 122.1, 113.8, 55.3, 27.1, 24.0, 22.1, 21.1; IR (thin film) 3039, 2933, 2836, 1606, 1573, 1517, 1447, 1289, 1247, 1223, 1174 cm^{-1}; HRMS (ESI) *m/z* calcd. for $C_{22}H_{24}NO_2$ $(M+H)^+$ 334.1807, found 334.1800.

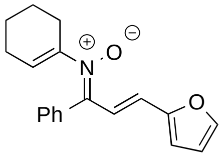

Nitrone 8da: General procedure **A** was applied using the following reagents: chalcone oxime **6d** (0.022 g, 0.10 mmol), cyclohex-1-enylboronic acid (**7a**, 0.041 g, 0.33 mmol), $Cu(OAc)_2$ (0.0029 g, 0.016 mmol), pyridine (0.062 mL, 0.80 mmol), and Na_2SO_4 (0.10 g, 0.70 mmol). No COD was added to this reaction mixture. This procedure afforded ***E*-8da** as a yellow liquid (0.021 g, 70%) after purification using medium pressure chromatography (2:1, EtOAc:hexanes). ^{1}H NMR (500 MHz, $CDCl_3$): δ 7.83 (d, *J* = 16.3 Hz, 1H), 7.50-7.34 (m, 4H), 7.34-7.17 (m, 2H), 6.51-6.25 (m, 3H), 5.64-5.48 (m, 1H), 2.31-2.28 (m, 2H), 1.93-1.76 (m, 2H), 1.64-1.50 (m, 2H), 1.44-1.28 (m, 2H); ^{13}C NMR (125 MHz, $CDCl_3$) δ 152.9, 147.7, 144.3, 143.8, 133.4, 129.7, 128.9, 128.4, 126.3, 125.7, 119.9, 112.2, 111.9, 27.0, 23.9, 22.0, 21.0; IR (thin film) 3054, 2932, 2859, 1603, 1501, 1440, 1361, 1264, 1248, 1074 cm^{1}; HRMS (ESI) m/z calcd for $C_{19}H_{20}NO_2$ $(M+H)^+$ 294.1485, found 294.1494.

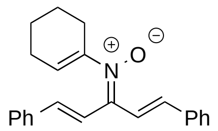

Nitrone 8ea: General procedure **A** was applied using the following reagents: dibenzylideneacetone (dba) oxime **6e** (0.025 g, 0.10 mmol), cyclohex-1-enylboronic acid (**7a**, 0.025 g, 0.20 mmol), $Cu(OAc)_2$ (0.0018 g, 0.010 mmol), pyridine (0.042 mL, 0.50 mmol), COD (0.015 mL, 0.12 mmol), and Na_2SO_4 (0.087 g, 0.61 mmol). This procedure afforded **8ea** as a yellow amorphous solid (0.027 g, 84%) after purification using medium pressure chromatography (2:1, EtOAc:hexanes). ^{1}H NMR (500 MHz, $CDCl_3$): δ 7.60-7.55 (m, 2H), 7.50 (d, *J* = 7.9 Hz, 2H), 7.47-7.43 (m, 2H), 7.40-7.36 (m, 4H), 7.35-7.30 (m, 2H), 7.02-6.92 (m, 2H), 6.03-5.95 (m, 1H), 2.56-2.41 (m, 2H), 2.27-2.21 (m, 2H), 1.91-1.77 (m, 2H), 1.78-1.64 (m, 2H); ^{13}C NMR (125 MHz, $CDCl_3$) δ 145.0, 144.3, 139.3, 136.6, 136.3, 134.1, 129.0, 129.0, 128.8, 128.7, 127.4, 126.7, 125.5, 120.3, 119.1, 26.3, 24.2, 22.3, 21.4; IR (thin film) 3024, 2932, 2858, 1493, 1447, 1374, 1304, 1217, 1188, 1074 cm^{-1}; HRMS (ESI) m/z calcd for $C_{23}H_{24}NO$ $(M+H)^+$ 330.1858, found 330.1851.

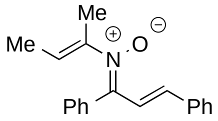

Nitrone 8ab: General procedure **B** was applied using the following reagents: chalcone oxime **6a** (0.230 g , 1.03 mmol), 2-buten-2-ylboronic acid (**7b**, 0.311 g, 3.12 mmol), $Cu(OAc)_2$ (0.219 g, 1.20 mmol), pyridine (0.90 mL, 10.0 mmol), Na_2SO_4 (1.35 g, 9.52 mmol). This procedure afforded ***E*-8ab** as a light yellow amorphous solid (0.042 g, 15%) after medium pressure chromatography (80:20 EtOAc:hexanes). ^{1}H NMR (500 MHz; $CDCl_3$) δ 8.03 (d, *J* = 15.0 Hz, 1H), 7.47-7.46 (m, 2H), 7.43-7.40 (m, 3H), 7.32-7.27 (m, 3H), 7.24-7.23 (m, 2H), 6.53 (d, *J* = 15.0 Hz, 1H), 5.44-5.40 (m, 1H), 1.91 (s, 3H), 1.41 (d, *J* = 7.0 Hz, 3H); ^{13}C NMR (125 MHz) δ 148.2, 142.0, 140.3, 136.3, 133.4, 129.8, 129.1, 129.0, 128.7, 128.5, 127.5, 123.7, 121.8, 15.2, 12.4; IR (thin film) 3055, 2925, 2854, 1663, 1604, 1449, 1335, 1214, 1015, 747 cm^{-1}; HRMS (ESI) *m/z* calcd. for $C_{19}H_{20}NO$ $(M+H)^+$ 278.1545, found 278.1542.

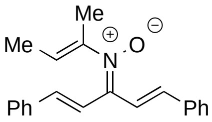

Nitrone 8bb: General procedure **B** was applied using the following reagents: dba oxime **6b** (0.260 g, 1.04 mmol), 2-buten-2-ylboronic acid (**7b**, 0.346 g, 3.46 mmol), $Cu(OAc)_2$ (0.212 g, 1.17 mmol), pyridine (0.80 mL, 10.0 mmol), Na_2SO_4 (1.24 g, 8.74 mmol). This procedure afforded **8bb** as an amorphous yellow solid (0.22 g, 68%) after medium pressure chromatography (60:40 EtOAc:hexanes). ^{1}H NMR (500 MHz; $CDCl_3$) δ 7.59-7.57 (m, 2H), 7.53-7.50 (m, 2H), 7.45-7.44 (m, 2H), 7.37 (q, *J* = 7.3 Hz, 4H), 7.33-7.31 (m, 2H), 6.95 (s, 2H), 5.72-5.68 (m, 1H), 2.12 (s, 3H), 1.79 (d, *J* = 6.8 Hz, 3H); ^{13}C NMR (125 MHz) δ 144.6, 141.9, 139.2, 136.7, 136.4, 134.0, 129.0, 128.9, 128.8, 128.7, 127.4, 126.8, 123.2, 120.4, 119.1, 14.6, 12.8; IR (thin film) 3055, 3025, 2925, 1694, 1597, 1447, 1216, 1191, 963, 753 cm^{-1}; HRMS (ESI) *m/z* calcd. for $C_{21}H_{22}NO$ $(M+H)^+$ 304.1701, found 304.1702.

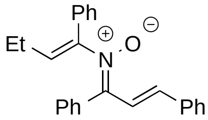

Nitrone 8ac: General procedure **B** was applied using the following reagents: chalcone oxime **6a** (0.066 g, 0.30 mmol), 1-phenylbut-1-enylboronic acid (**7c**, 0.16 g, 0.90 mmol), $Cu(OAc)_2$ (0.11 g, 0.59 mmol), pyridine (0.24 mL, 3.0 mmol), and Na_2SO_4 (0.30 g, 2.11 mmol). This procedure afforded ***E*-8ac** as a yellow liquid (0.061 g, 57%) after purification using medium pressure chromatography (2:1, EtOAc:hexanes). ^{1}H NMR (500 MHz; $CDCl_3$): δ ^{1}H NMR (500 MHz; $CDCl_3$): δ 8.10 (d, *J* = 16.5 Hz, 1H), 7.46 (d, *J* = 6.5 Hz, 2H), 7.35-7.22 (m, 9H), 7.13-7.12 (m, 2H), 7.03 (d, *J* = 8.0 Hz, 2H), 6.53 (d, *J* = 16.5 Hz, 1H), 5.97 (t, *J* = 7.5 Hz, 1H), 2.06-2.01 (m, 2H), 0.87 (t, *J* = 7.5 Hz, 3H); ^{13}C NMR (125 MHz, $CDCl_3$): δ 149.2, 145.3, 140.1, 136.3, 133.7, 132.8, 130.5, 129.2, 129.1, 128.8, 128.7, 128.3, 128.2, 128.1, 128.0, 127.6, 121.9, 21.2, 13.4; IR (thin film) 3025, 2965, 1681, 1598, 1575, 1493, 1447, 1274, 1213, 1175, 1071 cm^{-1}; HRMS (ESI) *m/z* calcd. for $C_{25}H_{24}NO$ $(M+H)^+$ 354.1858, found 354.1852.

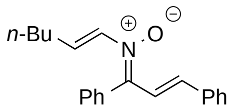

Nitrone 8ad: General procedure **B** was applied using the following reagents: chalcone oxime **6a** (0.066 g, 0.30 mmol), hex-1-enylboronic acid (**7d**, 0.10 g, 0.78 mmol), $Cu(OAc)_2$ (0.11 g, 0.59 mmol), pyridine (0.24 mL, 3.0 mmol), and Na_2SO_4 (0.36 g, 2.53 mmol). This procedure afforded ***E*-8ad** as a yellow liquid (0.068 g, 75%) after purification using medium pressure chromatography (2:1, EtOAc:hexanes). ^{1}H NMR (500 MHz; $CDCl_3$): δ 8.19 (d, J = 16.5 Hz, 1H), 7.52-7.40 (m, 5H), 7.30-7.26 (m, 5H), 6.89-6.83 (m, 1H), 6.61 (d, J = 13.0 Hz, 1H), 6.50 (d, J = 16.5 Hz, 1H), 2.06-2.01 (m, 2H), 1.36-1.33 (m, 2H), 1.29-1.25 (m, 2H), 0.85 (t, J = 7.0 Hz, 3H); ^{13}C NMR (125 MHz, $CDCl_3$): δ 147.9, 140.9, 136.4, 132.0, 130.9, 130.3, 129.7, 129.3, 129.0, 128.8, 128.7, 127.6, 122.5, 30.8, 29.0, 22.1, 13.7; IR (thin film) 3052, 2929, 1712, 1604, 1575, 1448, 1364, 1335, 1264, 1216 cm^{-1}; HRMS (ESI) m/z calcd. for $C_{21}H_{24}NO$ (M+H)+ 306.1858, found 306.1860.

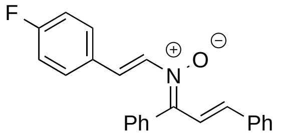

Nitrone 8ae: General procedure **B** was applied using the following reagents: chalcone oxime **6a** (0.066 g, 0.30 mmol), (*p*-fluorostyryl)boronic acid (**7e**, 0.15 g, 0.90 mmol), $Cu(OAc)_2$ (0.11 g, 0.59 mmol), pyridine (0.24 mL, 3.0 mmol), and Na_2SO_4 (0.36 g, 2.53 mmol). This procedure afforded ***E*-8ae** as a yellow solid (0.085 g, 83%) after purification using medium pressure chromatography (2:1, EtOAc:hexanes). ^{1}H NMR (500 MHz; $CDCl_3$): δ 8.27 (d, J = 16.5 Hz, 1H), 7.72 (d, J = 13.0 Hz, 1H), 7.57-7.55 (m, 3H), 7.51-7.49 (m, 2H), 7.40-7.38 (m, 2H), 7.32-7.31 (m, 3H), 7.26-7.21 (m, 3H), 6.97-6.94 (m, 2H), 6.58 (d, J = 16.5 Hz, 1H); ^{13}C NMR (125 MHz, $CDCl_3$): δ 164.1 (d, $J_{C\text{-}F}$ = 248.0 Hz), 149.5, 141.6, 136.4, 131.9, 130.8, 130.5, 130.1, 129.5, 129.3, 129.1, 128.8, 128.6, 127.8, 126.9, 122.5, 116.0; IR (thin film) 3111, 3056, 1965, 1887, 1741, 1631, 1599, 1569, 1507, 1446 cm^{-1}; HRMS (ESI) m/z calcd. for $C_{23}H_{19}FNO$ (M+H)+ 344.1451, found 344.1449.

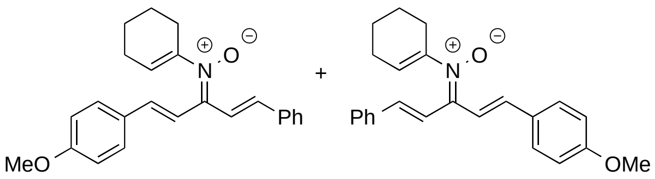

Nitrone 8fa/8fa': General procedure **A** was applied using the following reagents: oxime **6f** (0.119 g , 0.427 mmol), cyclohexenylboronic acid (**7a**, 0.181 g, 1.43 mmol), $Cu(OAc)_2$ (0.0080 g, 0.044 mmol), pyridine (0.17 mL, 2.1 mmol), cyclooctadiene (64 μL, 0.47 mmol), and Na_2SO_4 (0.589 g, 4.15 mmol). This procedure afforded **8fa:8fa'** as a yellow oil (0.12 g, 76%) and as a 1:1 mixture of nitrone isomers after medium pressure chromatography (60:40 EtOAc:hexanes). ^{1}H NMR (500 MHz; $CDCl_3$) *E*- and *Z*-isomers were coincidental δ 7.57 (d, *J* = 10 Hz, 1H), 7.52 (d, *J* = 10 Hz, 1H), 7.49-7.43 (m, 3H), 7.40-7.35 (m, 4H), 7.33-7.31 (m, 1H), 6.95 (m, 1H), 6.92-6.85 (m, 3H), 5.91-5.89 (m, 1H), 3.83 (s, 3H), 2.49-2.47 (m, 2H), 2.24-2.21 (m, 2H), 1.85-1.81 (m, 2H), 1.71-1.68 (m, 2H); ^{13}C NMR (125 MHz; $CDCl_3$) *E*- and *Z*-isomers were distinguishable δ 160.5, 145.3, 139.3, 136.7, 134.1, 129.4, 129.0, 128.9, 128.8, 128.2, 127.4, 125.4, 120.4, 119.3, 114.5, 55.4, 26.3, 24.2, 22.3, 21.5; ^{13}C NMR of other isomer (125 MHz; $CDCl_3$) δ 160.3, 144.3, 139.1, 136.4, 133.9, 129.1, 129.0, 128.8, 128.2, 127.4, 126.8, 125.3, 118.2, 116.9, 114.3, 55.3, 26.2, 24.2, 22.3, 21.5; IR (thin film) 3022, 2938, 2860, 1701, 1601, 1510, 1249, 1172, 1104, 1030 cm^{-1}; HRMS (ESI) *m/z* calcd. for $C_{24}H_{26}NO_2$ $(M+H)^+$ 360.1964, found 360.1964.

III. Synthesis of pyridines

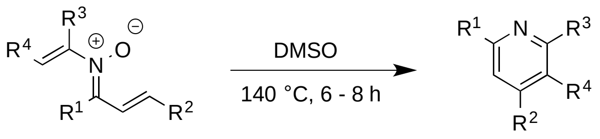

8 **9**

General procedure C: A Teflon-sealed reaction flask was charged with nitrone **8**, 4 Å molecular sieves (3 beads) and DMSO (0.1 M) and solution was heated at 140 °C for 6–8 h. The solution was then diluted with EtOAc (10 mL) and extracted with water. The aqueous layer was extracted twice with EtOAc (2 × 10 mL), and the combined organic extracts were washed with brine (1 × 10 mL). The organic layer was dried with $MgSO_4$ and concentrated under vacuum. The crude product mixture was purified by medium pressure chromatography (15:85, EtOAc:hexanes) to give **9** as a light yellow oil.

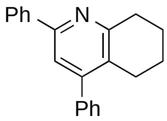

Pyridine 9aa:[3] General procedure C was applied using nitrone **8aa** (0.031 g, 0.10 mmol). This procedure afforded **9aa** as a yellow oil (0.020 g, 71%) after purification using medium pressure chromatography (15:85, EtOAc:hexanes). ^{1}H NMR (500 MHz, $CDCl_3$): δ 7.99-7.96 (m, 2H), 7.49-7.42 (m, 5H), 7.39-7.35 (m, 2H), 7.37-7.33 (m, 2H), 3.10 (t, *J* = 6.5 Hz, 2H), 2.66 (t, *J* = 6.3 Hz, 2H), 2.02-1.90 (m, 2H), 1.84-1.68 (m, 2H) ; ^{13}C NMR (125 MHz, $CDCl_3$) δ 157.6, 154.3, 150.3, 139.7, 128.9, 128.6, 128.5, 128.4, 128.3, 128.0, 127.7, 126.9, 119.1, 33.3, 27.3, 23.1, 23.0; IR (thin film) 3057, 2932, 2859, 1587, 1542, 1495, 1439, 1419, 1381, 1264, 1073 cm^{-1}; HRMS (ESI) m/z calcd for $C_{21}H_{20}N$ $(M+H)^+$ 286.1596, found 286.1599.

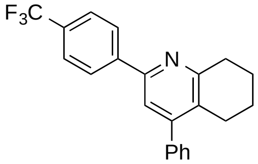

Pyridine 9ba: General procedure C was applied using nitrone **8ba** (0.027 g, 0.074 mmol). This procedure afforded **9ba** as a yellow amorphous solid (0.018 g, 68%) after purification using medium pressure chromatography (15:85, EtOAc:hexanes). ^{1}H NMR (500 MHz, $CDCl_3$): δ 8.11 (d, *J* = 8.1 Hz, 2H), 7.69 (d, *J* = 8.1 Hz, 2H), 7.49-7.41 (m,

4H), 7.36-7.33 (m, 2H), 3.11 (t, *J* = 6.6 Hz, 2H), 2.68 (t, *J* = 6.3 Hz, 2H), 2.06-1.88 (m, 2H), 1.84-1.71 (m, 2H); ^{13}C NMR (125 MHz, $CDCl_3$) δ 158.1, 152.5, 139.3, 130.9, 129.6, 129.0, 128.8, 128.7, 128.5, 128.4, 127.9, 127.1, 125.6 (q, $J_{C\text{-}F}$ = 204.0 Hz), 119.4, 33.2, 27.3, 23.0, 22.9; IR (thin film) 2937, 1714, 1587, 1541, 1496, 1449, 1322, 1264, 1164, 1108, 1067 cm^{-1}; HRMS (ESI) m/z calcd. for $C_{22}H_{19}F_3N$ $(M+H)^+$ 354.1470, found 354.1463.

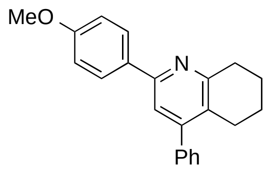

Pyridine 9ca:[3a] General procedure C was applied using nitrone **8ca** (0.015 g, 0.047 mmol). This procedure afforded **9ca** as a yellow oil (0.010 g, 64%) after purification using medium pressure chromatography (15:85, EtOAc:hexanes). ^{1}H NMR (500 MHz, $CDCl_3$): δ 7.93 (d, *J* = 8.7 Hz, 2H), 7.48-7.43 (m, 2H), 7.43-7.39 (m, 1H), 7.36-7.32 (m, 3H), 6.97 (d, *J* = 8.7 Hz, 2H), 3.85 (s, 3H), 3.08 (t, *J* = 6.6 Hz, 2H), 2.64 (t, *J* = 6.3 Hz, 2H), 2.01-1.86 (m, 2H), 1.82-1.68 (m, 2H); ^{13}C NMR (125 MHz, $CDCl_3$) δ 160.1, 157.3, 141.1, 140.1, 139.8, 128.5, 128.3, 128.1, 127.7, 127.5, 127.2, 118.5, 114.0, 55.3, 33.29, 29.72, 27.25, 23.12; IR (thin film) 2931, 2856, 1606, 1587, 1513, 1494, 1449, 1414, 1379, 1249, 1171 cm^{-1}; HRMS (ESI) *m/z* calcd. for $C_{22}H_{22}NO$ $(M+H)^+$ 316.1701, found 316.1702.

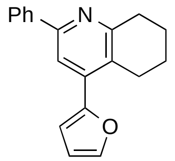

Pyridine 9da:[4] General procedure C was applied using nitrone **8da** (0.016 g, 0.046 mmol). This procedure afforded **9da** as a yellow oil (0.012 g, 76%) after purification using medium pressure chromatography (15:85, EtOAc:hexanes). ^{1}H NMR (500 MHz, $CDCl_3$): δ 8.04-7.99 (m, 2H), 7.90 (s, 1H), 7.60-7.58 (m, 1H), 7.48-7.44 (m, 2H), 7.41-7.38 (m, 1H), 6.78 (d, *J* = 3.4 Hz, 1H), 6.57 (dd, *J* = 3.4, 1.8 Hz, 1H), 3.19-3.06 (m, 2H), 2.94 (t, *J* = 6.2 Hz, 2H), 2.05-1.81 (m, 4H); ^{13}C NMR (125 MHz, $CDCl_3$) δ 162.8, 161.9, 158.0, 143.0, 128.6, 128.4, 128.1, 126.9, 126.8, 126.5, 115.4, 112.1, 111.8, 27.9, 27.7, 23.1, 22.7; IR (thin film) 2930, 1712, 1594, 1565, 1546, 1491, 1445, 1368, 1264, 1026 cm^{-1}; HRMS (ESI) *m/z* calcd. for $C_{19}H_{18}NO$ $(M+H)^+$ 276.1388, found 276.1389.

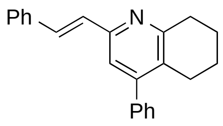

Pyridine 9ea: General procedure C was applied using nitrone **8ea** (0.050 g, 0.15 mmol). This procedure afforded **9ea** as a yellow oil (0.020 g, 43%) after purification using medium pressure chromatography (15:85, EtOAc:hexanes). ^{1}H NMR (500 MHz, $CDCl_3$): δ 7.59-7.54 (m, 2H), 7.51 (d, *J* = 16.2 Hz, 1H), 7.48-7.43 (m, 2H), 7.43-7.40 (m, 1H), 7.38-7.31 (m, 4H), 7.30-7.27 (m, 1H), 7.22-7.18 (m, 2H), 3.05 (t, *J* = 6.6 Hz, 2H), 2.63 (t, *J* = 6.4 Hz, 2H), 2.01-1.87 (m, 2H), 1.83-1.68 (m, 2H); ^{13}C NMR (125 MHz, $CDCl_3$) δ 157.4, 152.5, 139.5, 136.9, 131.8, 128.7, 128.6, 128.5, 128.4, 128.3, 128.2, 128.0, 127.7, 126.9, 119.8, 33.2, 27.4, 23.0, 23.0; IR (thin film) 3026, 2933, 2859, 1712, 1581, 1537, 1495, 1449, 1386, 1264, 1073 cm^{-1}; HRMS (ESI) m/z calcd. for $C_{23}H_{22}N$ $(M+H)^+$ 312.1752, found 312.1750.

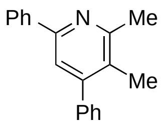

Pyridine 9ab:[5] General procedure C was applied using nitrone **8ab** (0.0421 g, 0.152 mmol). This procedure afforded **9ab** as a yellow oil (0.0198 g, 50%) after purification using medium pressure chromatography (5:95, EtOAc:hexanes). ^{1}H NMR (500 MHz, $CDCl_3$): δ 7.58-7.55 (m, 3H), 7.48-7.45 (m, 1H), 7.43-7.41 (m, 1H), 7.37-7.32 (m, 3H), 7.28-7.26 (m, 1H), 7.20-7.17 (m, 2H), 2.63 (s, 3H), 2.20 (s, 3H); ^{13}C NMR (125 MHz, $CDCl_3$) δ 157.7, 151.9, 149.9, 140.1, 137.0, 131.5, 128.7, 128.6, 128.3, 128.0, 127.7, 126.9, 120.4, 23.6, 16.2; IR (thin film) 3055, 3022, 2922, 1580, 1545, 1495, 1448, 1388, 966, 779 cm^{-1}; HRMS (ESI) *m/z* calcd. for $C_{19}H_{18}N$ $(M+H)^+$ 260.1439, found 260.1437.

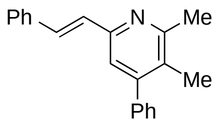

Pyridine 9eb: General procedure C was applied using nitrone **8eb** (0.0642 g, 0.212 mmol). This procedure afforded **9eb** as a yellow oil (0.0217 g, 36%) after purification using medium pressure chromatography (5:95, EtOAc:hexanes). ^{1}H NMR (500 MHz, $CDCl_3$): δ 7.58-7.55 (m, 3H), 7.48-7.45 (m, 2H), 7.43-7.32 (m, 5H), 7.29-7.27 (m, 1H), 7.20-7.17 (m, 2H), 2.63 (s, 3H), 2.20 (s, 3H); ^{13}C NMR (125 MHz, $CDCl_3$) δ 157.7, 152.0, 150.0, 140.1, 137.0, 131.6, 128.8, 128.7, 128.4, 128.3, 128.0, 127.7, 127.6, 127.0,

120.5, 23.7, 16.2; IR (thin film) 3058, 2967, 1721,1575, 1495, 1416, 1257, 1087, 964, 761 cm^{-1}; HRMS (ESI) *m/z* calcd. for $C_{21}H_{20}N$ $(M+H)^+$ 286.1596, found 286.1592.

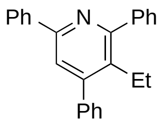

Pyridine 9ac:[6] General procedure C was applied using nitrone **8ac** (0.090 g, 0.25 mmol). This procedure afforded **9ac** as a yellow oil (0.073 g, 87%) after purification using medium pressure chromatography (15:85, EtOAc:hexanes). ^1H NMR (500 MHz, $CDCl_3$): δ 8.08 (d, *J* = 7.5 Hz, 2H), 7.63 (d, *J* = 7.5 Hz, 2H), 7.58 (s, 1H), 7.51-7.44 (m, 10H), 7.39 (t, *J* = 7.0 Hz, 1H), 2.74 (q, *J* = 7.5 Hz, 2H), 0.81 (t, *J* = 7.5 Hz, 3H); ^{13}C NMR (125 MHz, $CDCl_3$): δ 159.6, 153.7, 151.4, 141.7, 140.4, 139.2, 133.5, 129.1, 128.8, 128.7, 128.6, 128.5, 128.4, 128.2, 127.7, 127.0, 120.7, 22.2, 14.9; IR (thin film) 3057, 2970, 1714, 1584, 1575, 1491, 1443, 1415, 1374, 1264, 1087 cm^{-1}; HRMS (ESI) m/z calcd. for $C_{25}H_{22}N$ $(M+H)^+$ 336.1752, found 336.1759.

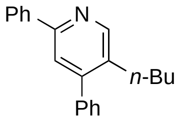

Pyridine 9ad:[7] General procedure C was applied using nitrone **8ad** (0.059 g, 0.20 mmol). This procedure afforded **9ad** as a yellow oil (0.038 g, 71%) after purification using medium pressure chromatography (15:85, EtOAc:hexanes). ^1H NMR (500 MHz, $CDCl_3$): δ 8.59 (s, 1H), 8.01 (d, *J* = 8.0 Hz, 2H), 7.57 (s, 1H), 7.47-7.39 (m, 6H), 7.36-7.35 (m, 2H), 2.61 (t, *J* = 7.5 Hz, 2H), 1.48-1.45 (m, 2H), 1.27-1.24 (m, 2H), 0.82 (t, *J* = 7.5 Hz, 3H); ^{13}C NMR (125 MHz, $CDCl_3$): δ 154.8, 150.7, 150.0, 139.5, 139.2, 134.1, 129.0, 128.7, 128.5, 128.4, 127.9, 126.7, 121.3, 33.2, 29.7, 22.3, 13.7; IR (thin film) 3057, 2958, 2860, 2361, 1592, 1576, 1474, 1446, 1375, 1265, 1216 cm^{-1}; HRMS (ESI) m/z calcd. for $C_{21}H_{22}N$ $(M+H)^+$ 288.1752, found 288.1750.

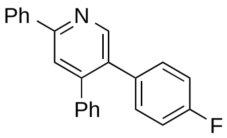

Pyridine 9ae: General procedure C was applied using nitrone **8ae** (0.047 g, 0.14 mmol). This procedure afforded **9ae** as a yellow oil (0.029 g, 65%) after purification using

medium pressure chromatography (15:85, EtOAc:hexanes). ^{1}H NMR (500 MHz, $CDCl_3$): δ 8.70 (s, 1H), 8.10-8.06 (m, 2H), 7.77 (s, 1H), 7.53-7.48 (m, 2H), 7.47-7.44 (m, 1H), 7.32-7.29 (m, 3H), 7.23-7.19 (m, 2H), 7.17-7.12 (m, 2H), 7.00-6.96 (m, 2H); ^{13}C NMR (125 MHz, $CDCl_3$) δ 163.2 (d, $J_{C\text{-}F}$ = 246.2 Hz), 156.4, 150.4, 138.7, 133.4, 131.4, 131.3, 129.2, 129.1, 128.8, 128.6, 128.4, 128.1, 126.9, 121.7, 115.4, 115.3; IR (thin film) 3057, 1711, 1586, 1537, 1510, 1494, 1471, 1443, 1367, 1264, 1222, 1158 cm^{-1}; HRMS (ESI) m/z calcd. for $C_{23}H_{17}FN$ $(M+H)^+$ 326.1345, found 326.1340.

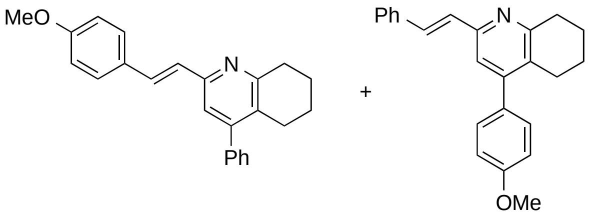

Pyridine 9fa: General procedure C was applied using nitrone **8fa:8fa'** (0.0895 g, 0.249 mmol). This procedure afforded **9fa:9fa'** as a yellow oil (0.0272 g, 32%) after purification using medium pressure chromatography (40:60, EtOAc:hexanes). ^{1}H NMR of **9fa:9fa'** (500 MHz, $CDCl_3$): δ 7.56 (d, *J* = 5 Hz, 1H), 7.52-7.39 (m, 3H), 7.38-7.32 (m, 2H), 7.28-7.27 (m, 1H), 7.21-7.15 (m, 2H), 7.09-7.05 (m, 1H), 6.98 (d, *J* = 5 Hz, 1H), 6.89 (d, *J* = 5 Hz, 1H), 3.87 (s, 3H), 3.05-3.02 (m, 2H), 2.67-2.61 (m, 2H), 1.94-1.91 (m, 2H), a second OMe resonance indicated the presence of the other isomer δ 3.82 (s, 3H); ^{13}C NMR of pyridine **9fa** (125 MHz, $CDCl_3$) δ 159.6, 157.5, 150.0, 139.7, 131.6, 129.8, 128.7, 128.5, 128.3, 128.2, 128.0, 127.0, 126.5, 120.0, 114.1, 55.4, 33.3, 27.6, 27.4, 23.1; ^{13}C NMR of pyridine **9fa'** (125 MHz, $CDCl_3$) δ 159.3, 157.4, 149.7, 137.0, 131.3, 129.8, 128.7, 128.5, 128.3, 128.2, 127.7, 127.0, 126.5, 119.5, 113.8, 55.4, 33.3, 27.4, 23.0; IR (thin film) 3061, 2930, 2857, 1607, 1510, 1450, 1246, 1174, 1033, 830 cm^{-1}; HRMS (ESI) *m/z* calcd. for $C_{24}H_{24}NO$ $(M+H)^+$ 342.1858, found 342.1863.

IV. References:

1. a) Mo, D.-L.; Anderson, L. L. *Angew. Chem. Int. Ed.* **2013**, *52*, 6722-6725; b) Mo, D.-L.; Pecak, W. H.; Zhao, M.; Wink, D. J.; Anderson, L. L. *Org. Lett.* **2014**, *16*, 3696-3699.

2. a) Patil, A. S.; Mo, D.-L.; Wang, H.-Y.; Mueller, D. S.; Anderson, L. L. *Angew. Chem. Int. Ed.* **2012**, *51*, 7799-7803; b) Kontokosta, D.; Mueller, D. S.; Wang, H.-Y.; Anderson, L. L. *Org. Lett.* **2013**, *15*, 4830-4833.

3. a) Gezegen, H.; Dingil, A.; Ceylan, M. *J. Heterocycl. Chem.* **2010**, *47*, 1017-1024; b) Katritzky, A. R.; Awartani, R. *Tetrahedron* **1982**, *38*, 2505-2512.

4. Banerjee, C. K.; Umarye, J. D.; Kanjilal, P. R. *Synth. Commun.* **2013**, *43*, 2208-2216.

5. Yamamoto, S.-I.; Okamoto, K.; Murakoso, M.; Kuninobu, Y.; Takai, K. *Org. Lett.* **2012**, *14*, 3182-3185.

6. Fischer, G. W. *J. Prakt. Chem.* **1985**, *327*, 983-997.

7. Liu, S.; Liebeskind, L. S. *J. Am. Chem. Soc.* **2008**, *130*, 6918-6919.

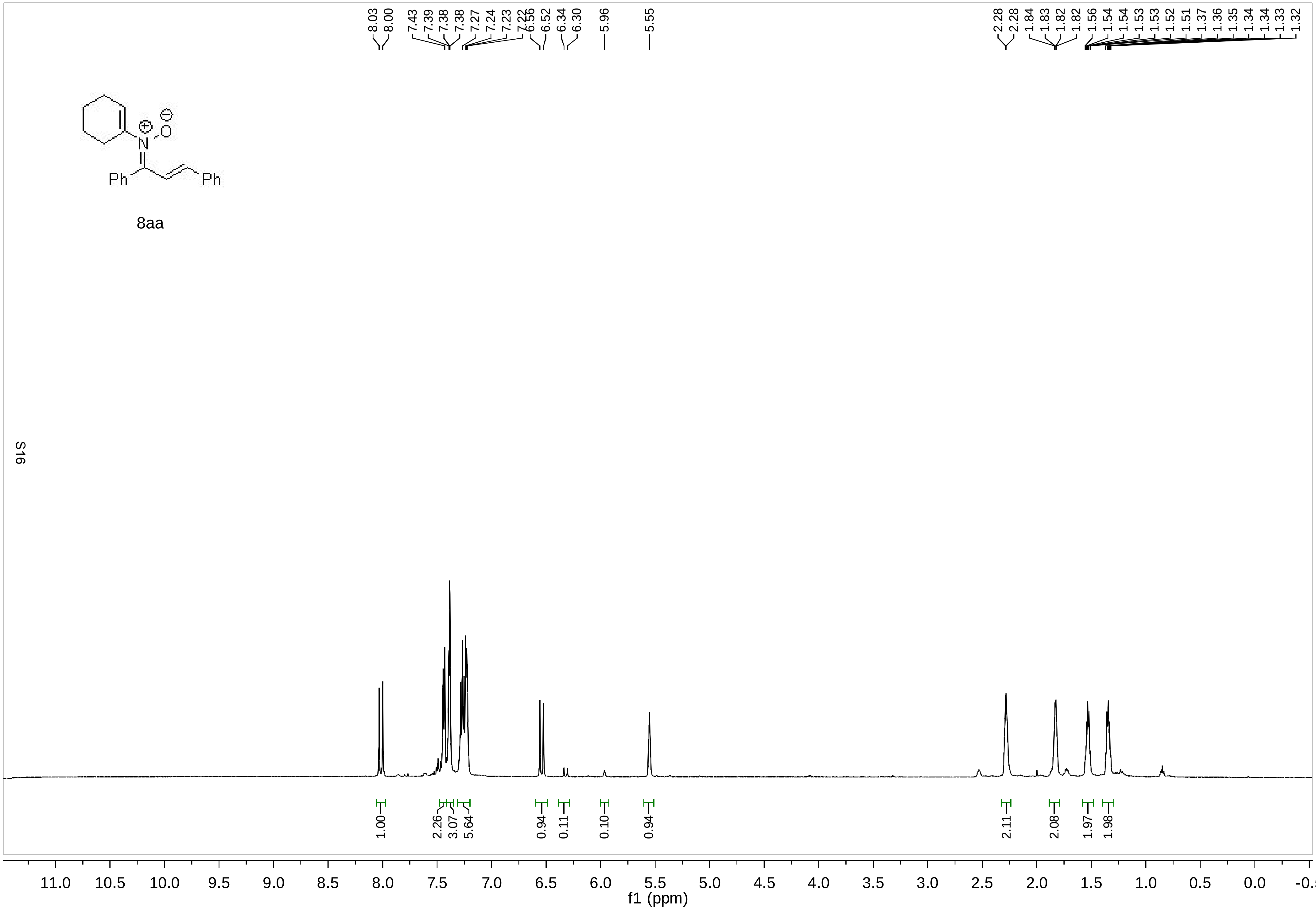
8.03
8.00
7.43
7.39
7.38
7.38
7.27
7.24
7.23
7.22
6.56
6.52
6.34
6.30
5.96
5.55
2.28
2.28
1.84
1.83
1.82
1.82
1.56
1.54
1.54
1.53
1.53
1.52
1.51
1.37
1.36
1.35
1.34
1.34
1.33
1.32
Ph
N
O
Ph
1.00
2.26
3.07
5.64
0.94
0.11
0.10
0.94
2.11
2.08
1.97
1.98
11.0 10.5 10.0 9.5 9.0 8.5 8.0 7.5 7.0 6.5 6.0 5.5 5.0 4.5 4.0 3.5 3.0 2.5 2.0 1.5 1.0 0.5 0.0 -0.5
f1 (ppm)

8aa

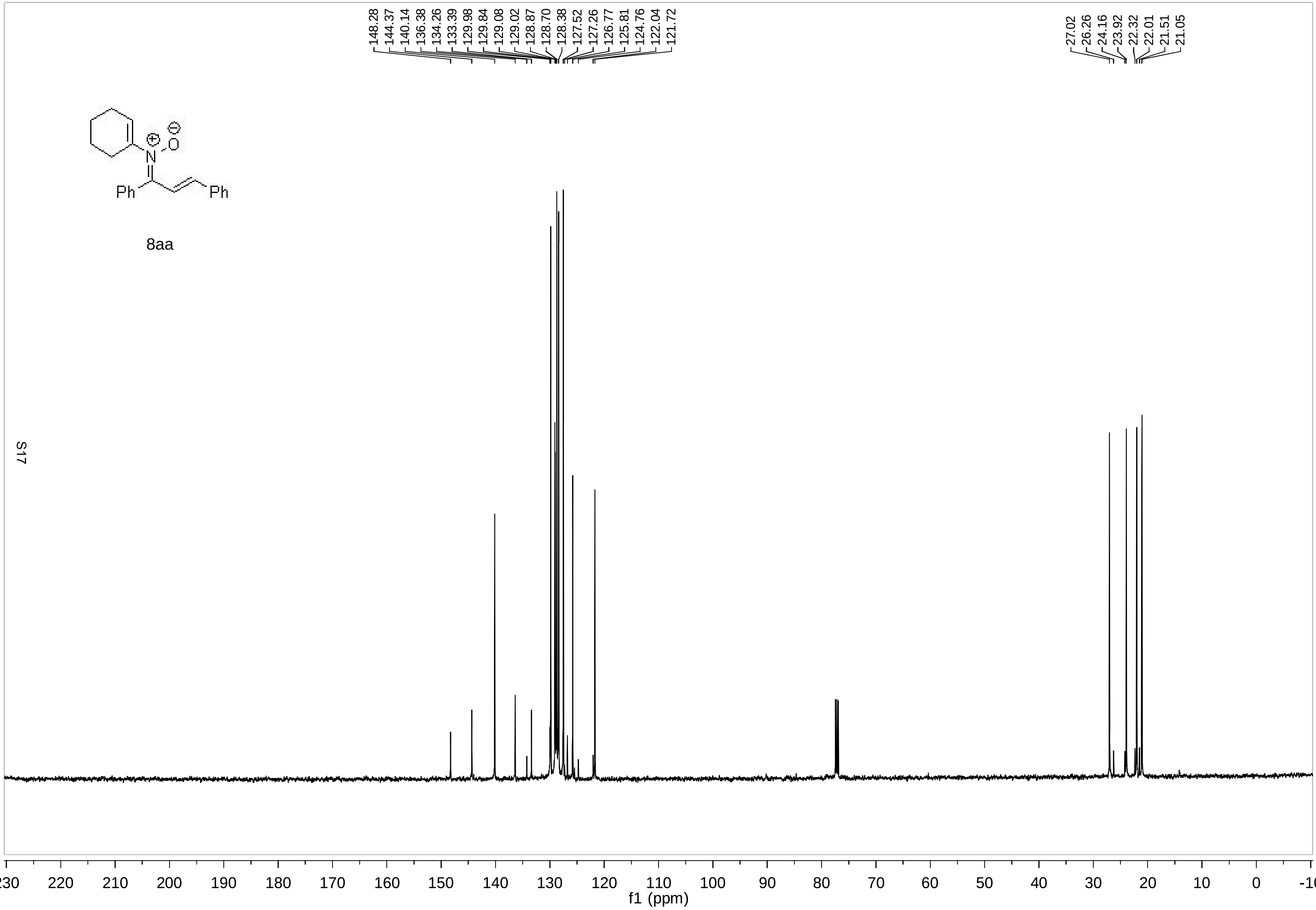
148.28
144.37
140.14
136.38
134.26
133.39
129.98
129.84
129.08
129.02
128.87
128.70
128.38
127.52
127.26
126.77
125.81
124.76
122.04
121.72
27.02
26.26
24.16
23.92
22.32
22.01
21.51
21.05
Ph
Ph
N
O
8aa
230
220
210
200
190
180
170
160
150
140
130
120
110
100
90
80
70
60
50
40
30
20
10
0
-10
f1 (ppm)

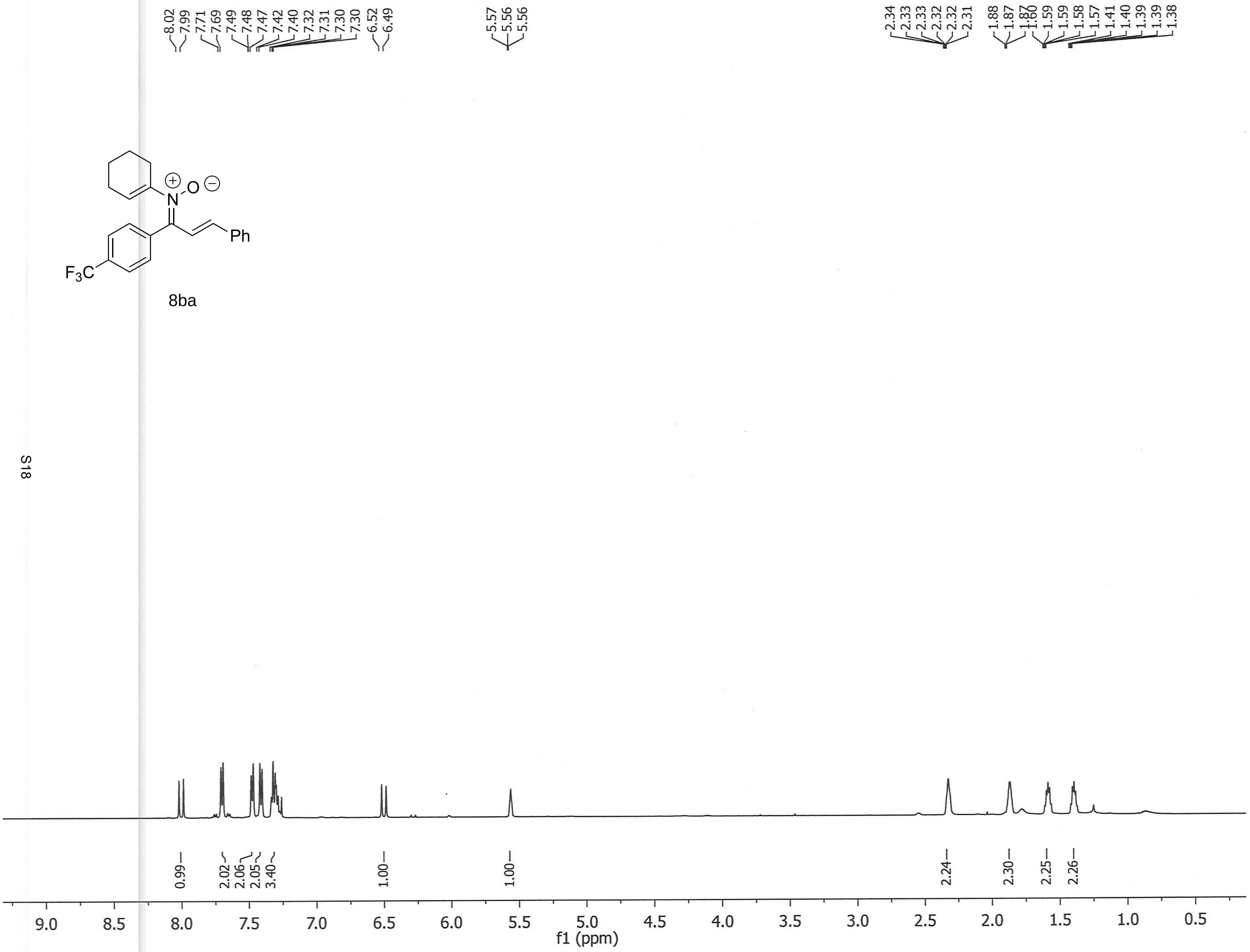
8.02
7.99
7.71
7.69
7.49
7.48
7.47
7.42
7.40
7.32
7.31
7.30
7.30
6.52
6.49
5.57
5.56
5.56
2.34
2.33
2.33
2.32
2.32
2.31
1.88
1.87
1.87
1.60
1.59
1.59
1.58
1.57
1.41
1.40
1.39
1.39
1.38
0.99
2.02
2.06
2.05
3.40
1.00
1.00
2.24
2.30
2.25
2.26
f1 (ppm)
Ph
F3C

8ba

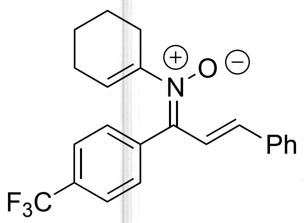

O
N
Ph
F3C

8ba

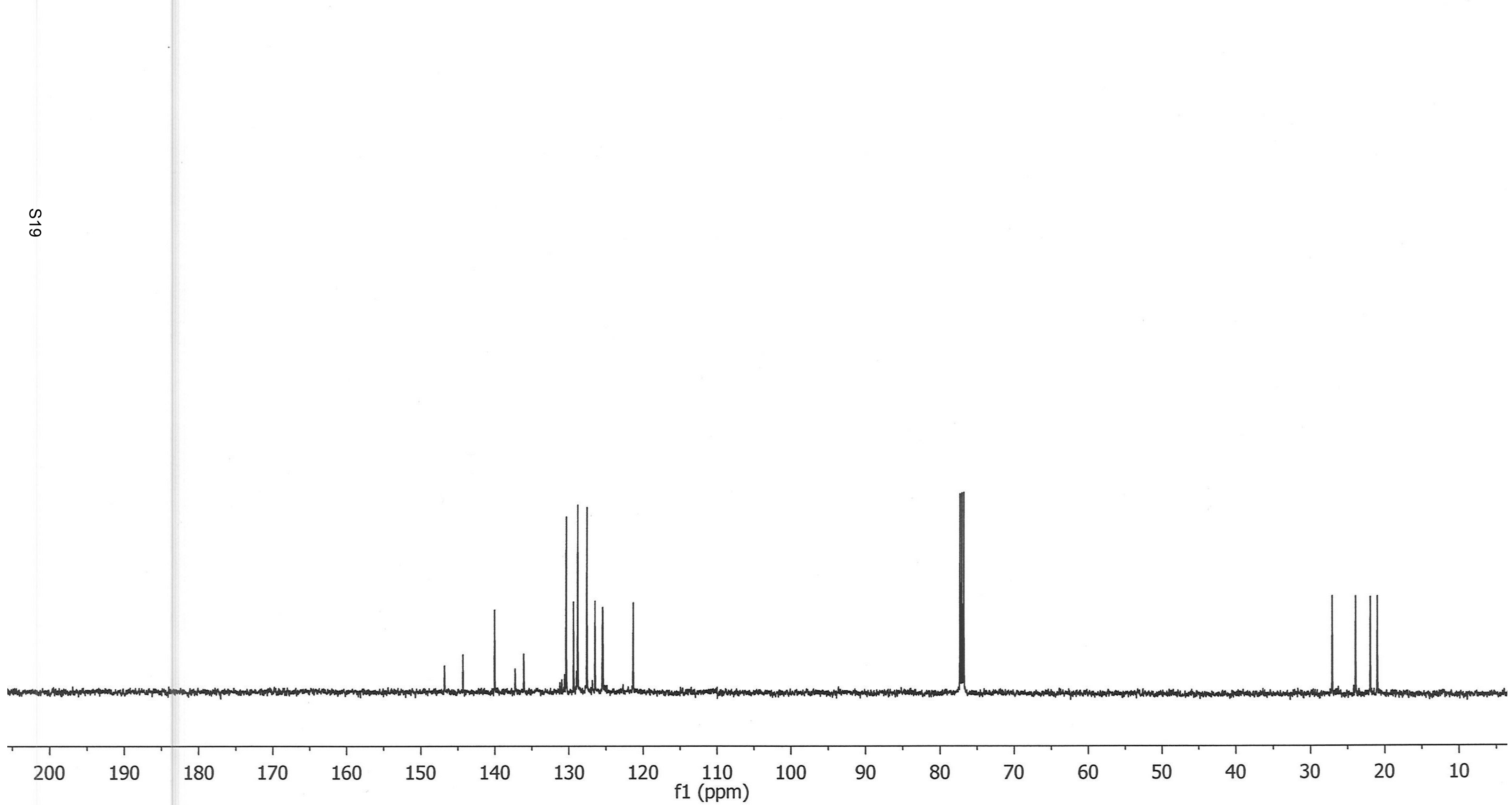

146.81
144.32
140.03
137.27
136.10
130.58
130.36
129.36
128.95
128.80
127.56
126.46
125.45
125.41
121.34
27.06
23.93
21.96
21.00
200
190
180
170
160
150
140
130
120
110
100
90
80
70
60
50
40
30
20
10
f1 (ppm)

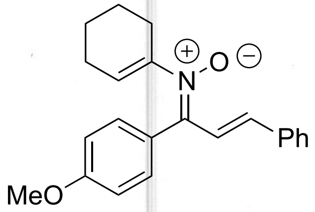

8ca

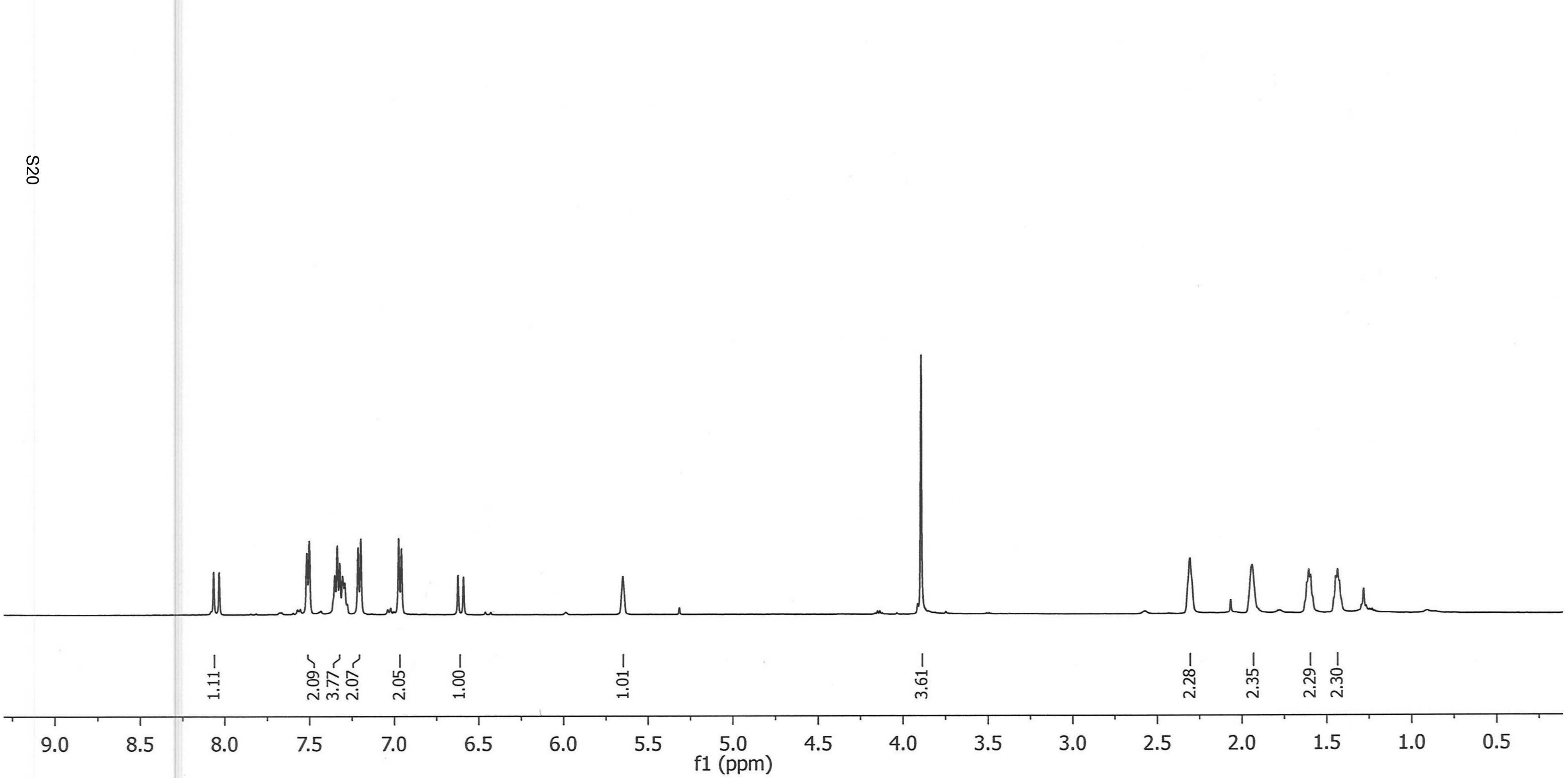
8.07
8.03
7.52
7.50
7.35
7.34
7.32
7.31
7.21
7.20
6.97
6.96
6.62
6.59
5.65
3.89
2.31
2.29
1.94
1.94
1.93
1.60
1.59
1.45
1.43
1.42
1.11
2.09
3.77
2.07
2.05
1.00
1.01
3.61
2.28
2.35
2.29
2.30
9.0
8.5
8.0
7.5
7.0
6.5
6.0
5.5
5.0
4.5
4.0
3.5
3.0
2.5
2.0
1.5
1.0
0.5
f1 (ppm)

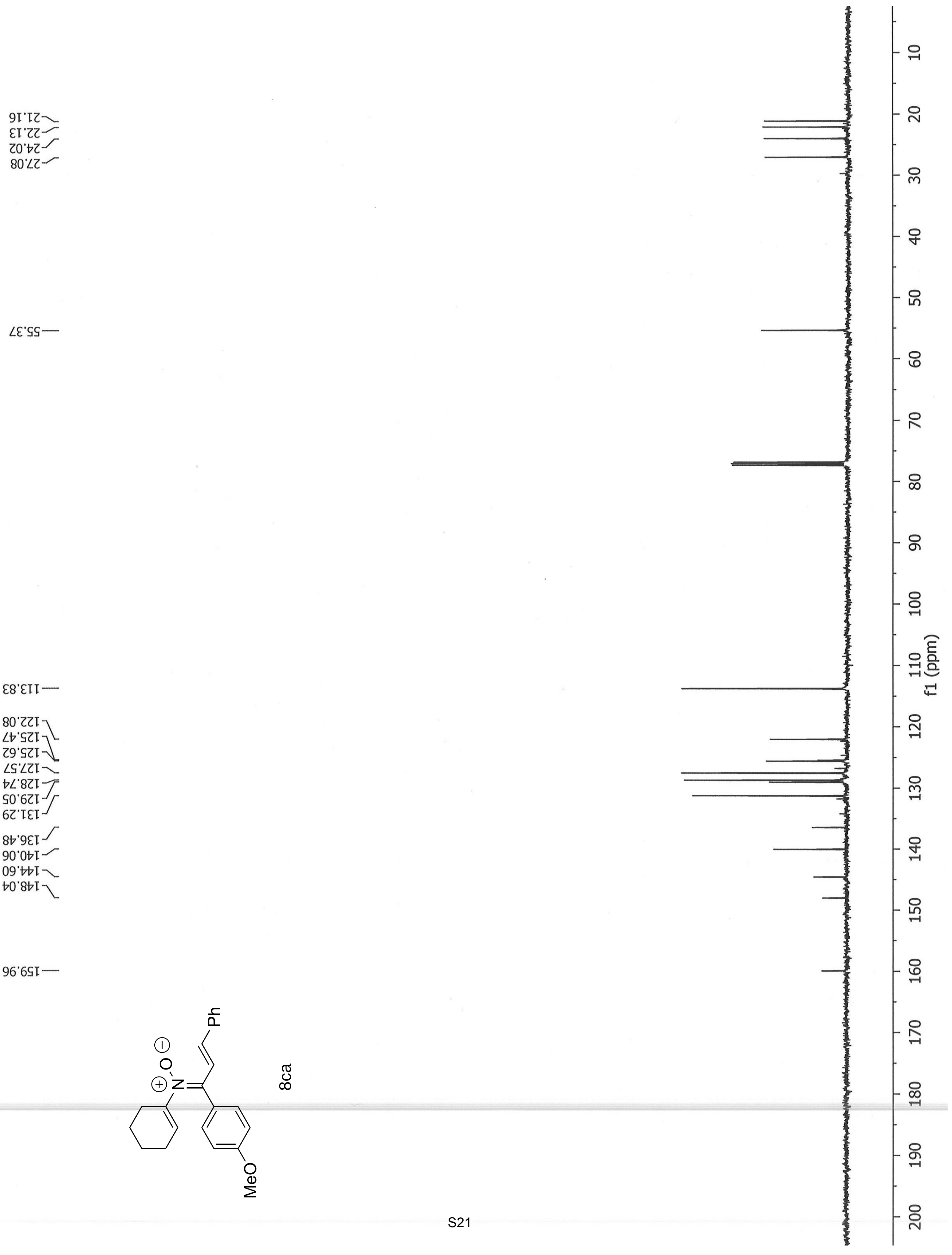
21.16
22.13
24.02
27.08
55.37
113.83
122.08
125.47
125.62
127.57
128.74
129.05
131.29
136.48
140.06
144.60
148.04
159.96
Ph
O
N
MeO
f1 (ppm)
10
20
30
40
50
60
70
80
90
100
110
120
130
140
150
160
170
180
190
200

8ca

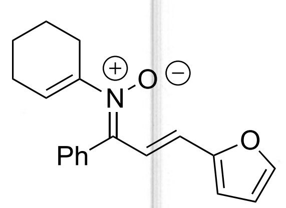

8da

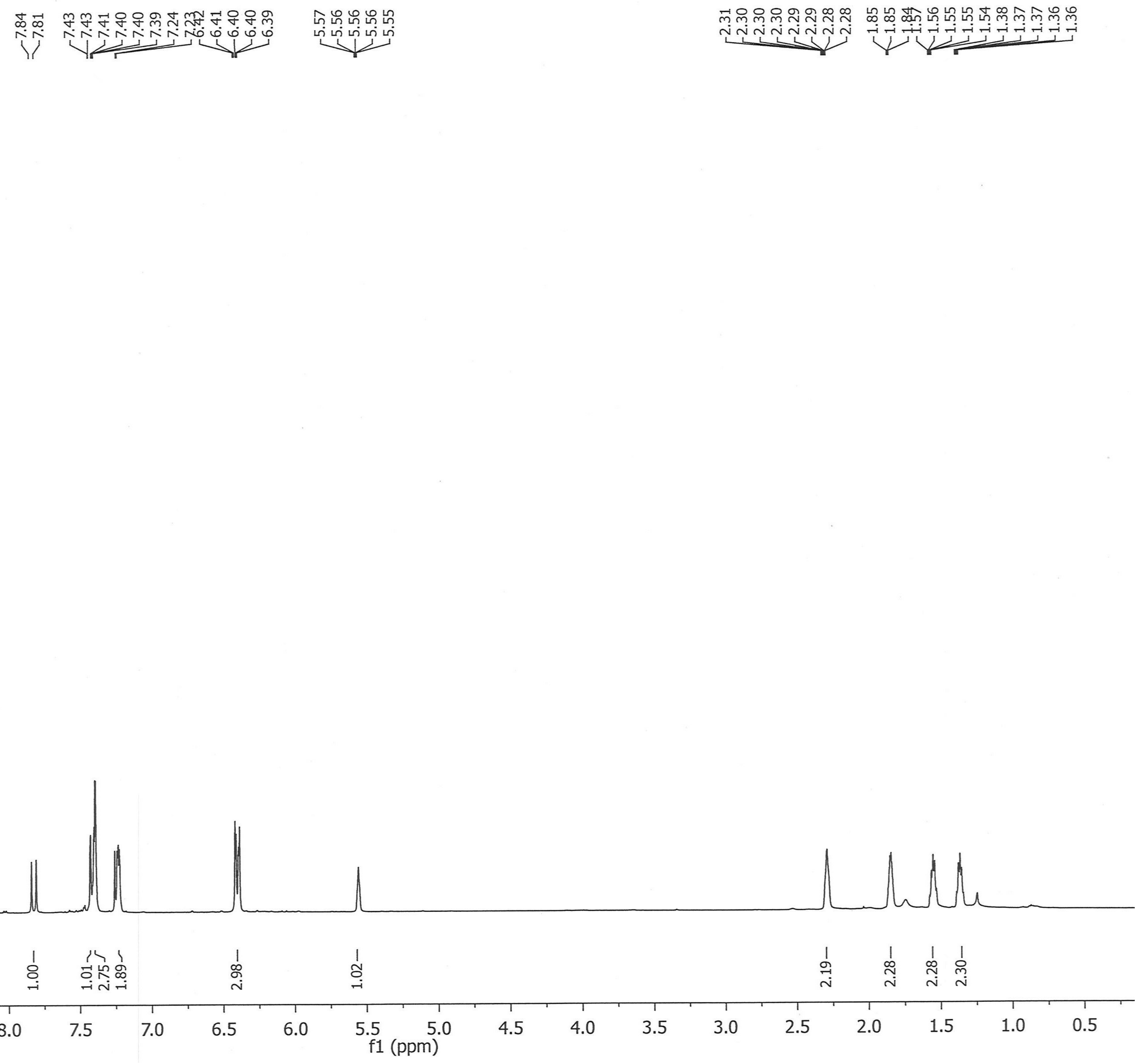
7.84
7.81
7.43
7.43
7.41
7.40
7.40
7.39
7.24
7.23
6.42
6.41
6.40
6.40
6.39
5.57
5.56
5.56
5.56
5.55
2.31
2.30
2.30
2.30
2.29
2.29
2.28
2.28
1.85
1.85
1.84
1.57
1.56
1.55
1.55
1.54
1.38
1.37
1.37
1.36
1.36
1.00
1.01
2.75
1.89
2.98
1.02
2.19
2.28
2.28
2.30
f1 (ppm)

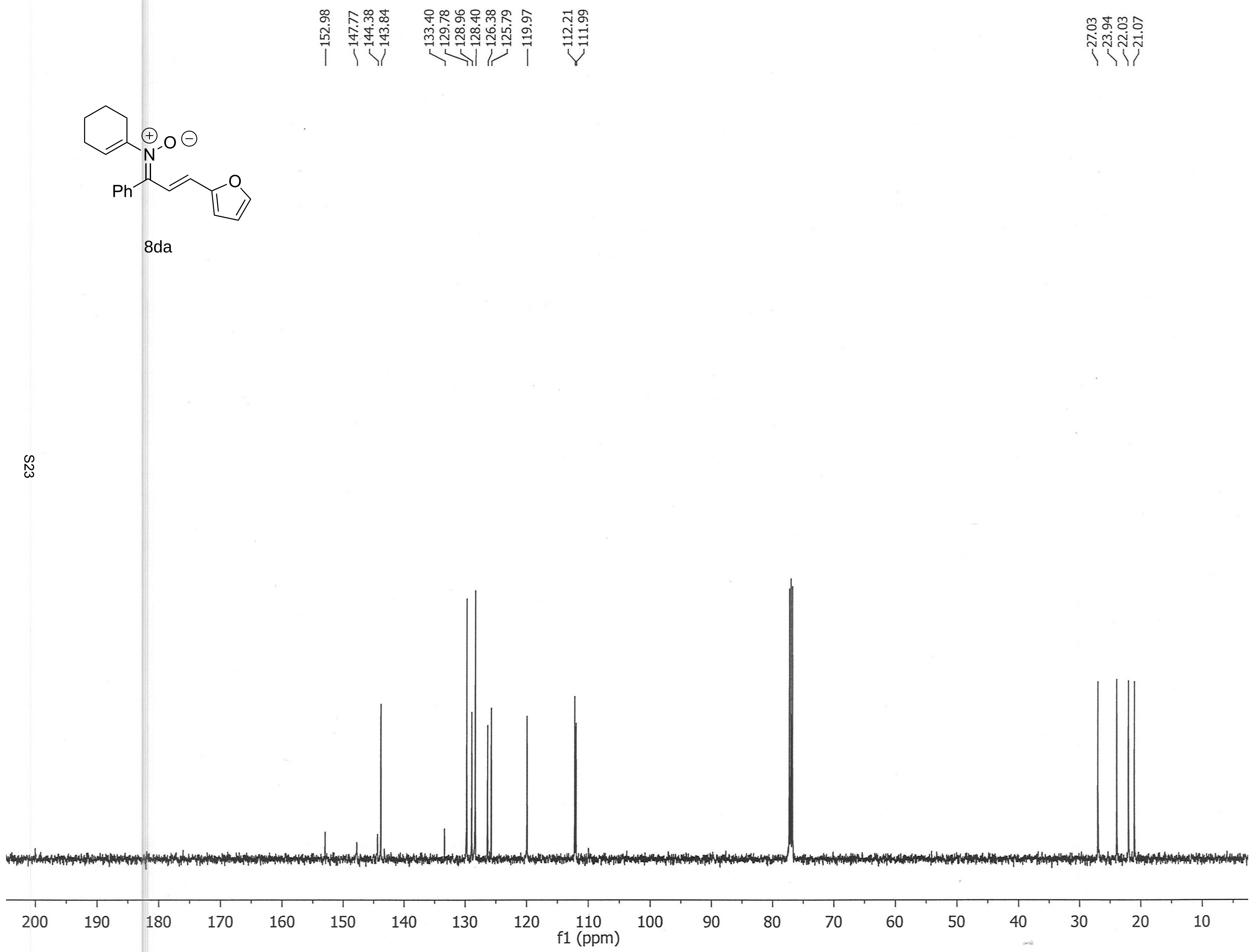

152.98
147.77
144.38
143.84
133.40
129.78
128.96
128.40
126.38
125.79
119.97
112.21
111.99
27.03
23.94
22.03
21.07
Ph
N
O
200 190 180 170 160 150 140 130 120 110 100 90 80 70 60 50 40 30 20 10
f1 (ppm)

8da

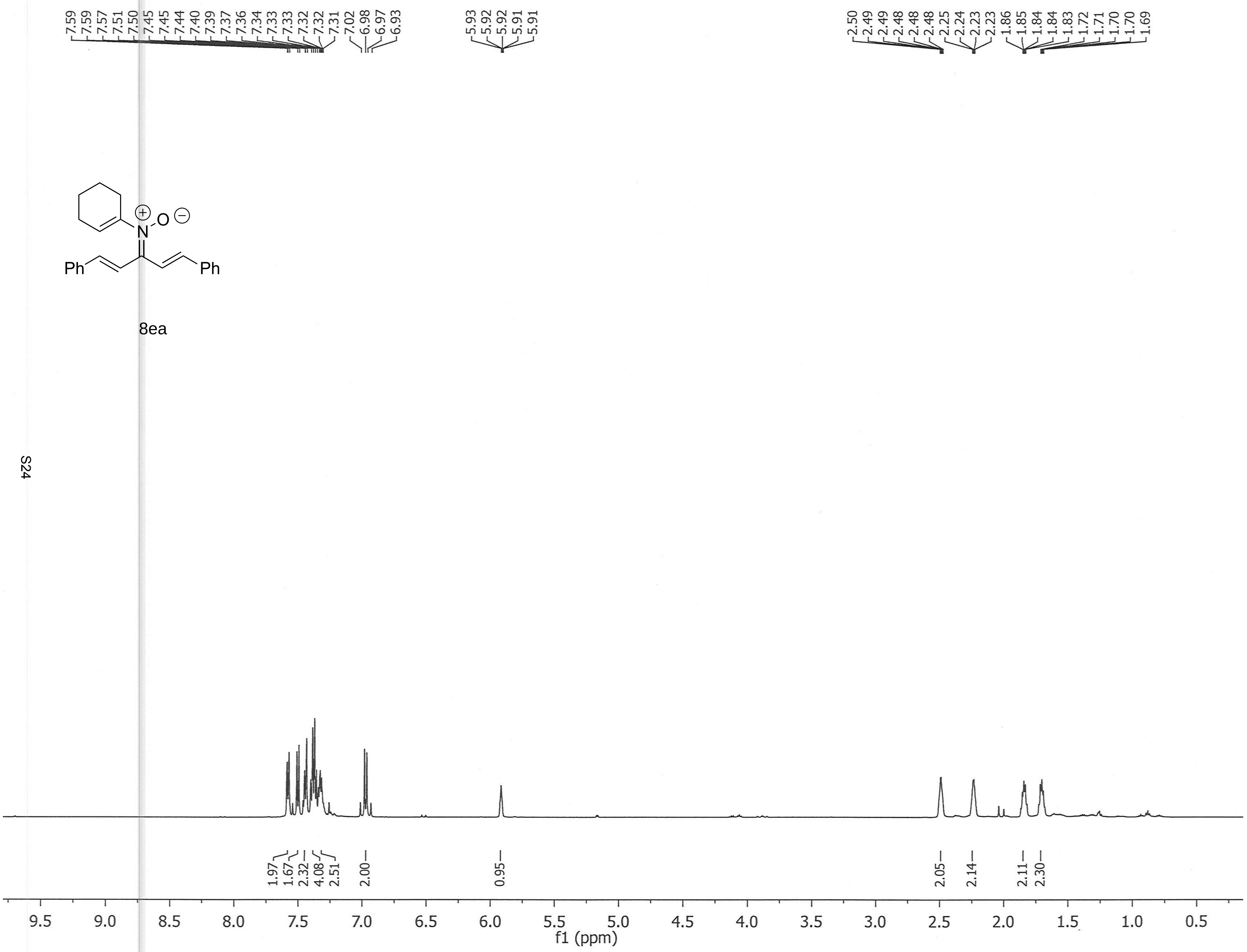

8ea

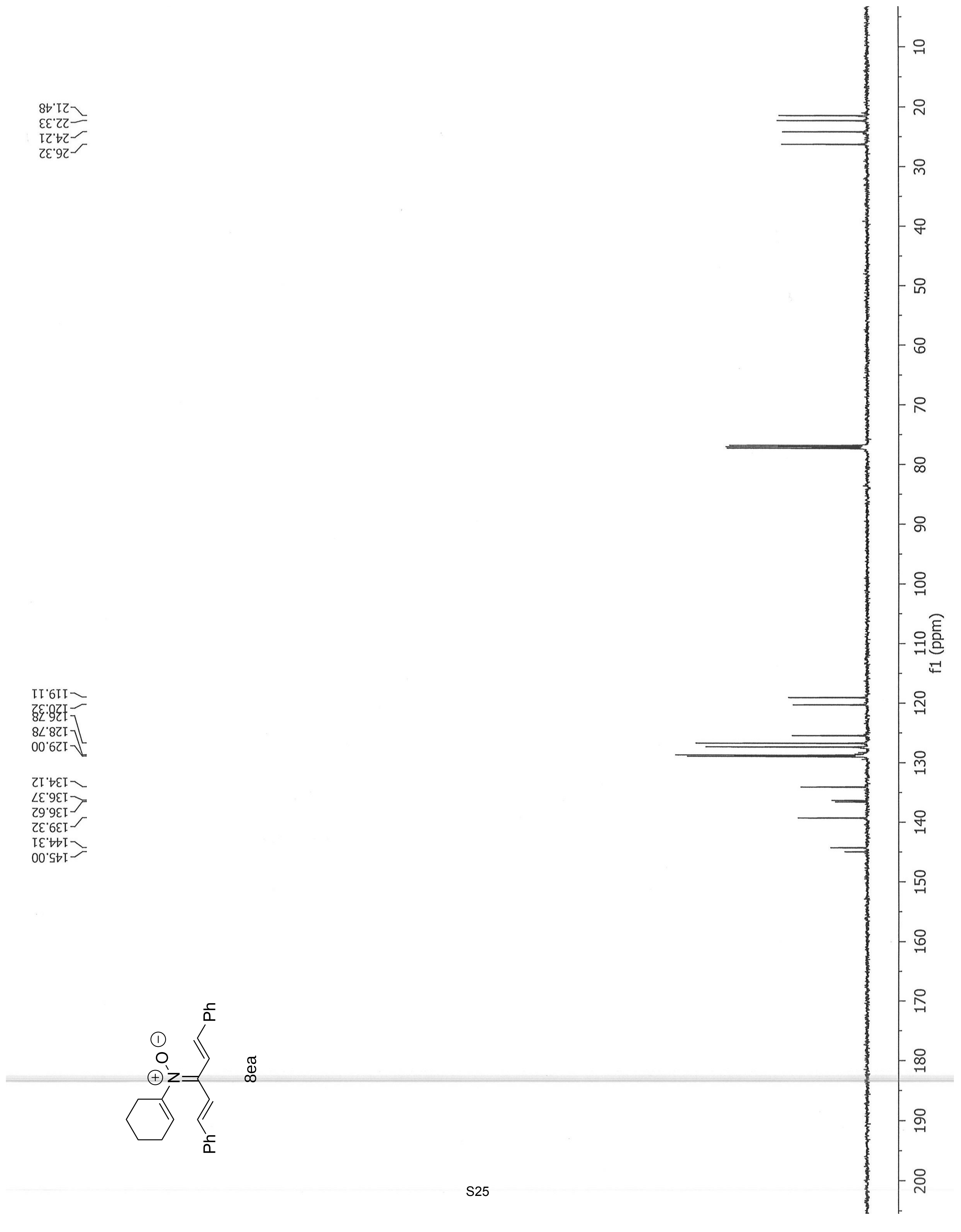
21.48
22.33
24.21
26.32
119.11
120.32
126.78
128.78
129.00
134.12
136.37
136.62
139.32
144.31
145.00
Ph
Ph
8ea
f1 (ppm)

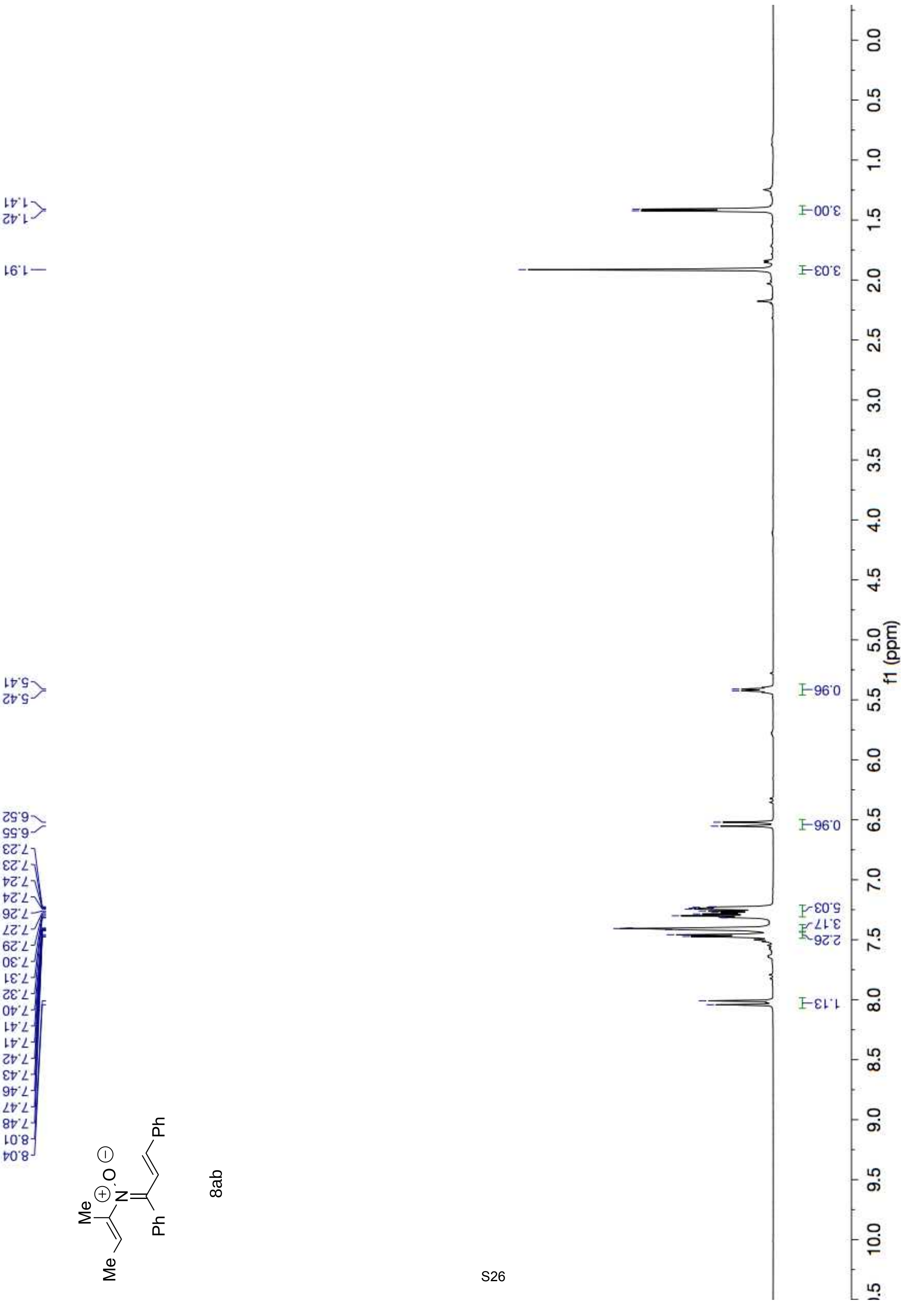

8ab

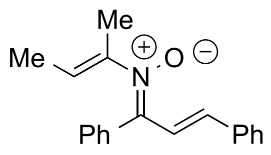

8ba

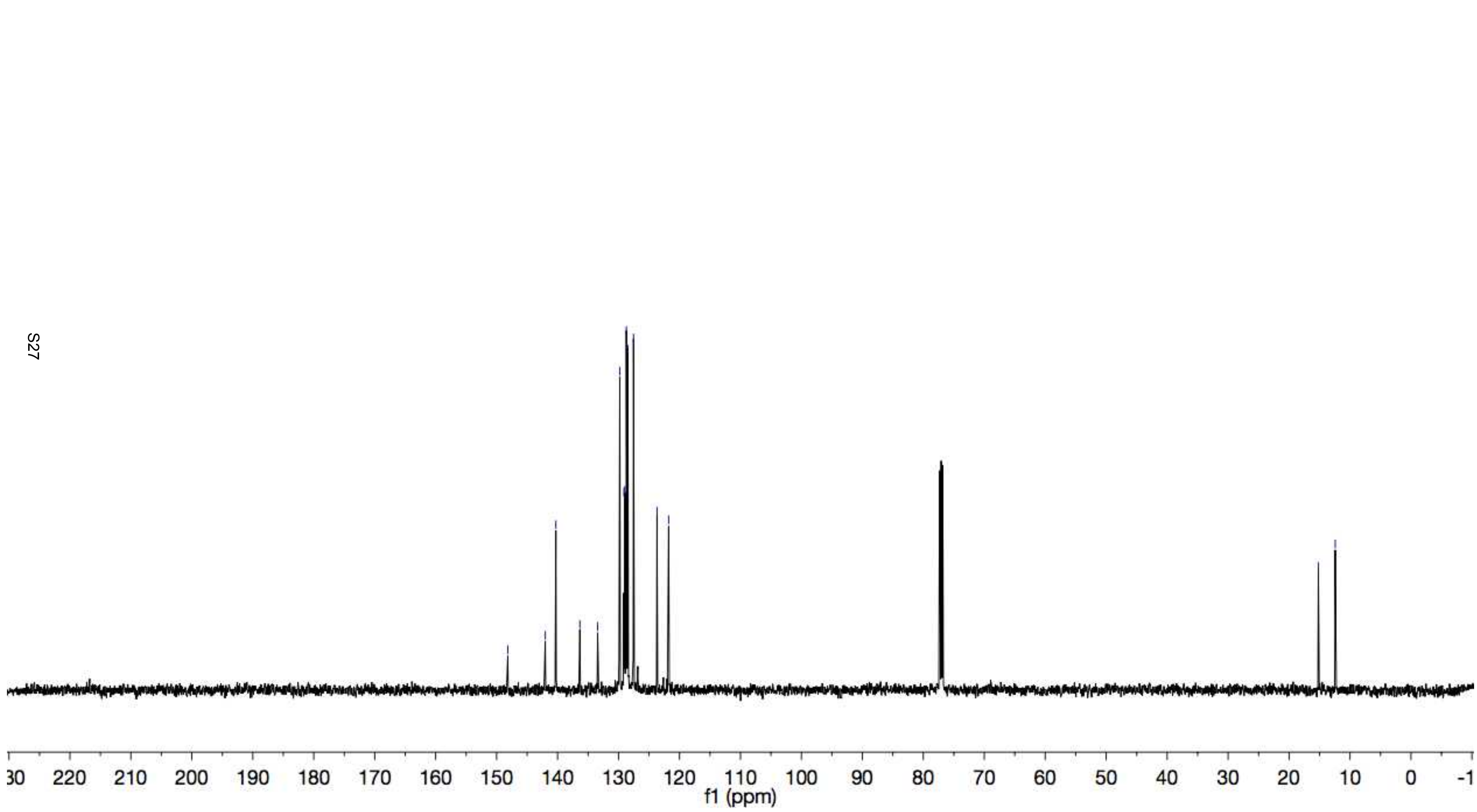

148.17
142.02
140.28
136.34
133.43
129.79
129.11
128.97
128.72
128.48
127.54
123.69
121.78
15.19
12.42
230 220 210 200 190 180 170 160 150 140 130 120 110 100 90 80 70 60 50 40 30 20 10 0 -1
f1 (ppm)

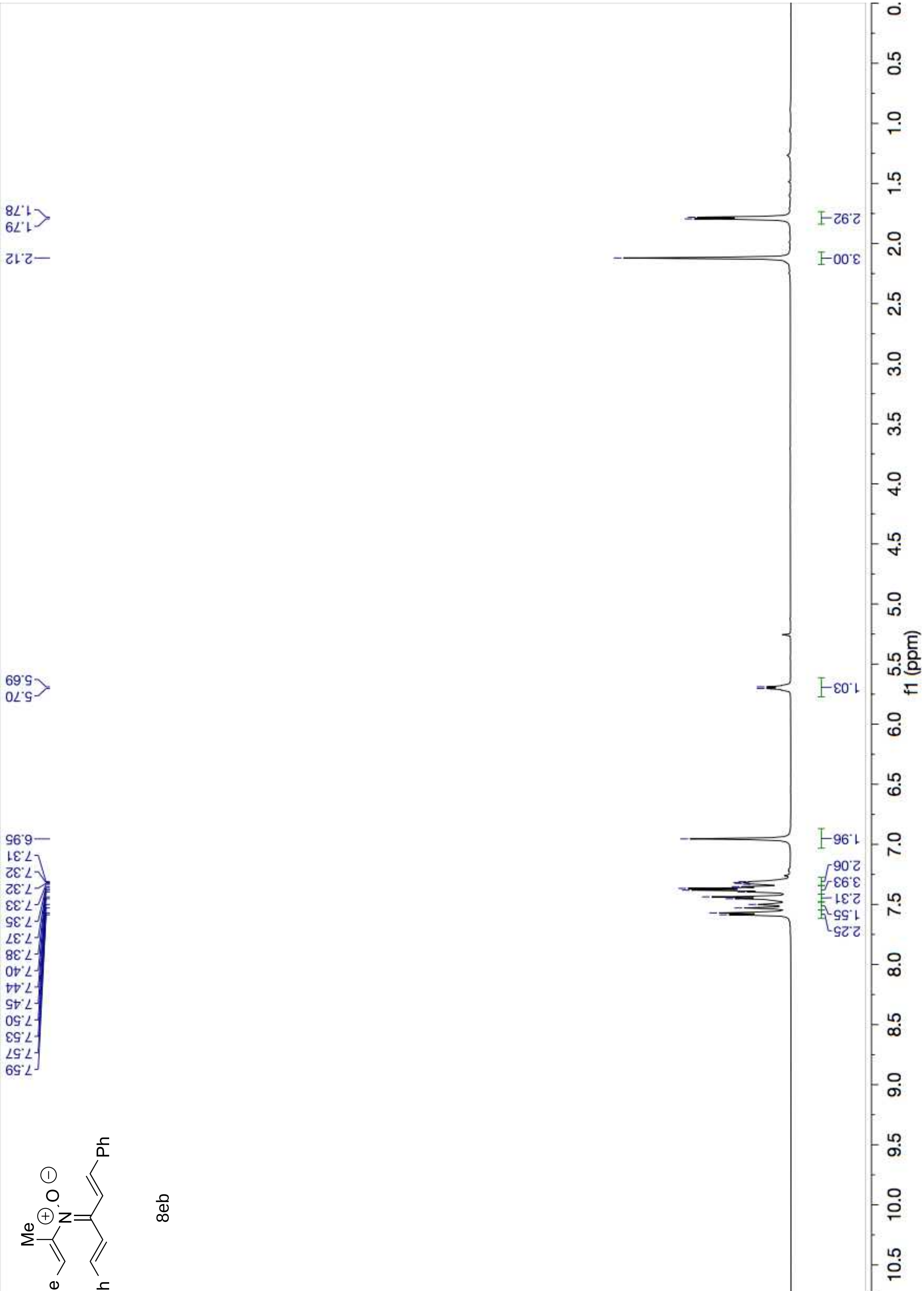

1.78
1.79
2.12
5.69
5.70
6.95
7.31
7.32
7.32
7.33
7.35
7.37
7.38
7.40
7.44
7.45
7.50
7.53
7.57
7.59
2.92
3.00
1.03
1.96
2.06
3.93
2.31
1.55
2.25
f1 (ppm)
8eb

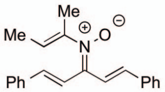
Me
Me
N
O
Ph
Ph

8eb

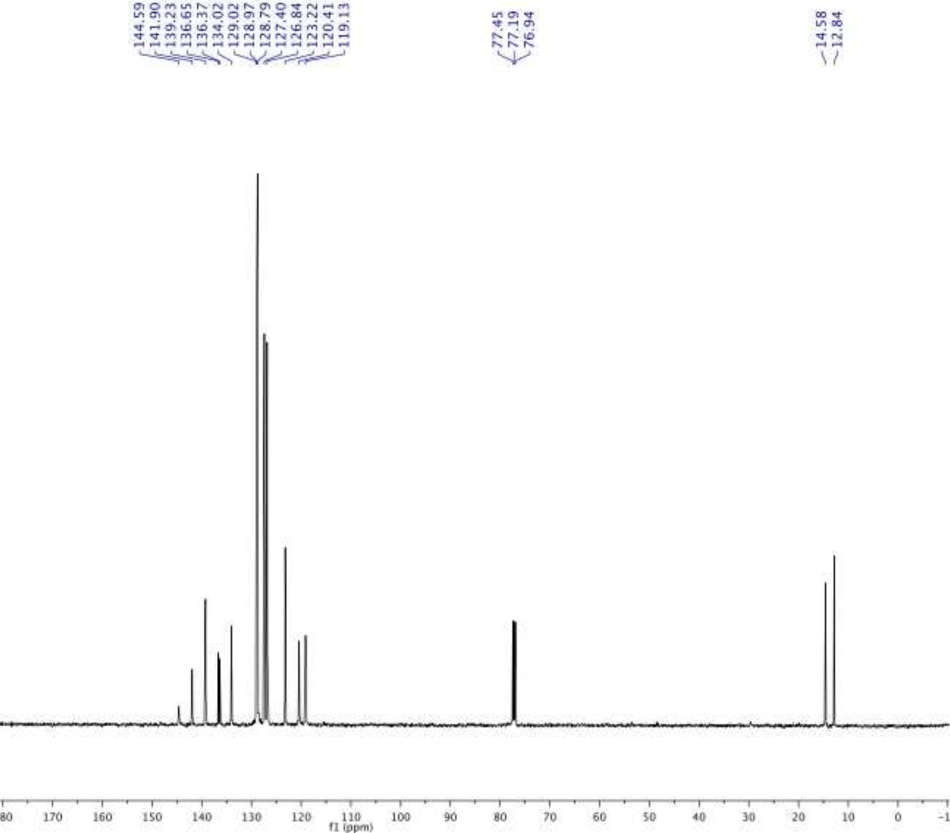
144.59
141.90
139.23
136.65
136.37
134.02
129.02
128.97
128.79
127.40
126.84
123.22
120.41
119.13
77.45
77.19
76.94
14.58
12.84
230
220
210
200
190
180
170
160
150
140
130
120
110
100
90
80
70
60
50
40
30
20
10
0
-1
f1 (ppm)

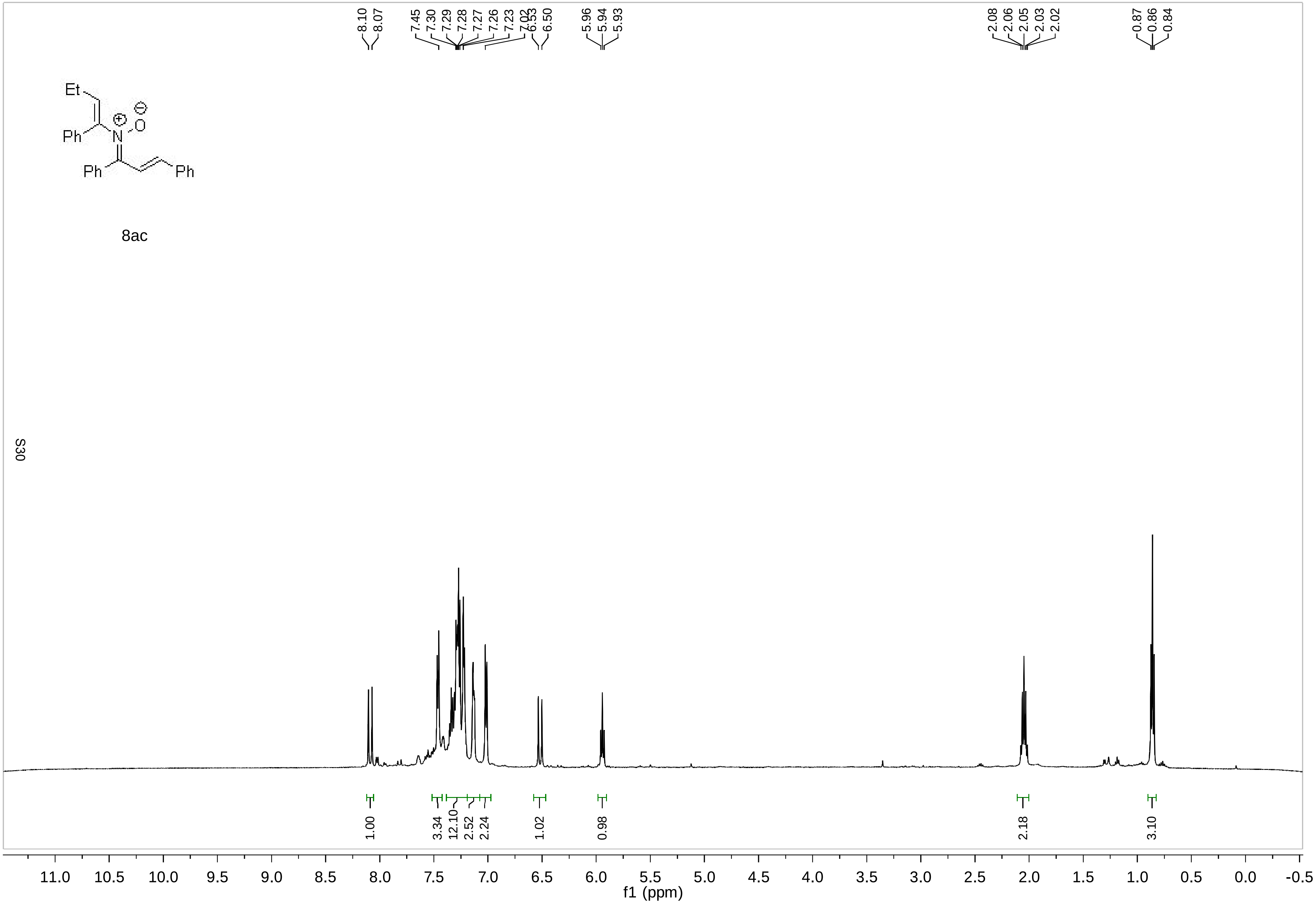

Et
Ph
N
O
Ph
Ph
8.10
8.07
7.45
7.30
7.29
7.28
7.27
7.26
7.23
7.02
6.53
6.50
5.96
5.94
5.93
2.08
2.06
2.05
2.03
2.02
0.87
0.86
0.84
1.00
3.34
12.10
2.52
2.24
1.02
0.98
2.18
3.10
11.0
10.5
10.0
9.5
9.0
8.5
8.0
7.5
7.0
6.5
6.0
5.5
5.0
4.5
4.0
3.5
3.0
2.5
2.0
1.5
1.0
0.5
0.0
-0.5
f1 (ppm)

8ac

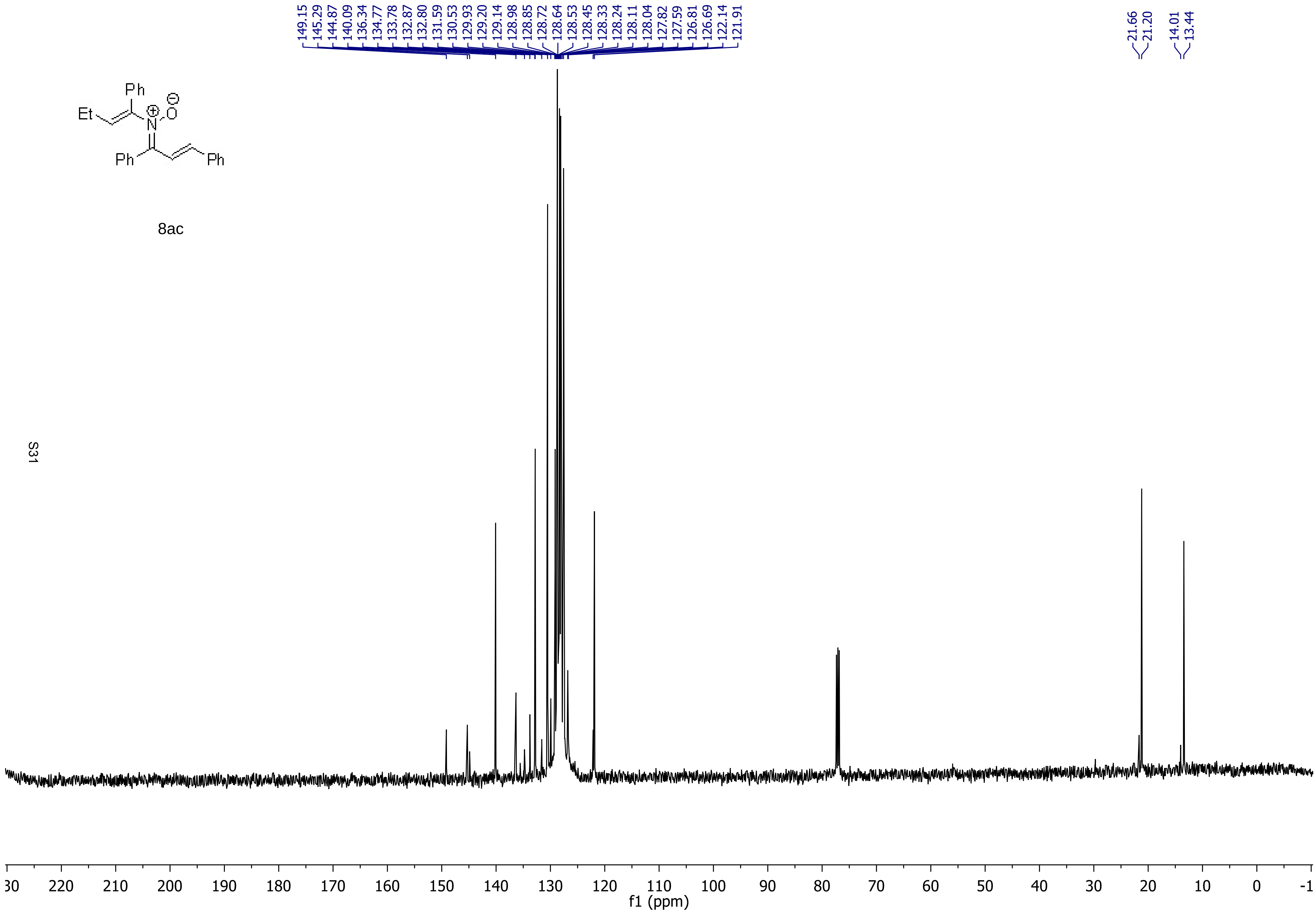

8ac

149.15
145.29
144.87
140.09
136.34
134.77
133.78
132.87
132.80
131.59
130.53
129.93
129.20
129.14
128.98
128.85
128.72
128.64
128.53
128.45
128.33
128.24
128.11
128.04
127.82
127.59
126.81
126.69
122.14
121.91
21.66
21.20
14.01
13.44
f1 (ppm)

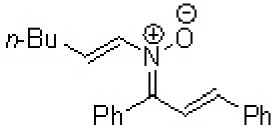

8ad

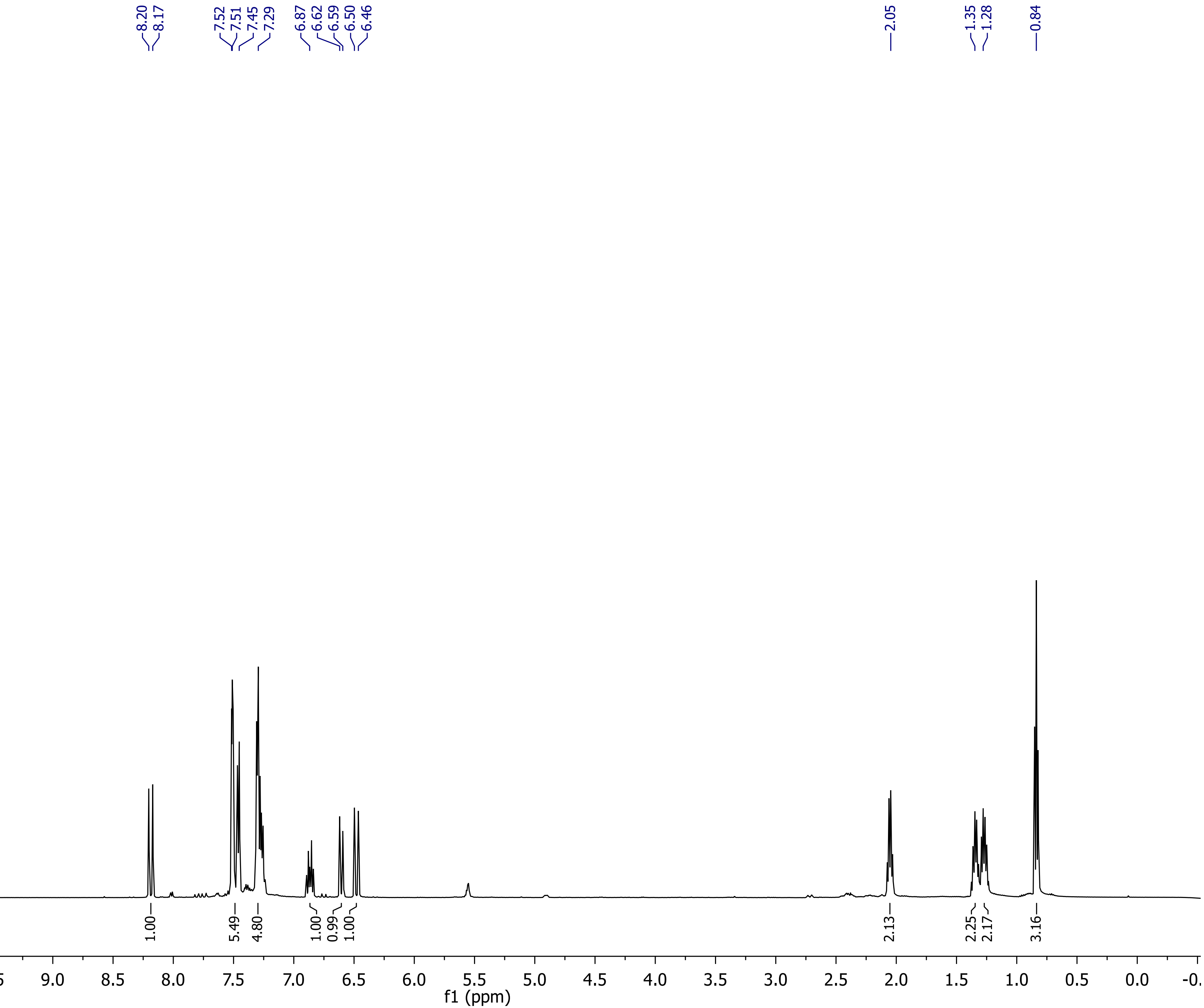
8.20
8.17
7.52
7.51
7.45
7.29
6.87
6.62
6.59
6.50
6.46
2.05
1.35
1.28
0.84
1.00
5.49
4.80
1.00
0.99
1.00
2.13
2.25
2.17
3.16
f1 (ppm)

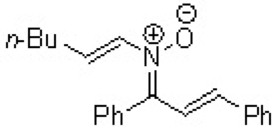

n-Bu
N
O
Ph
Ph

8ad

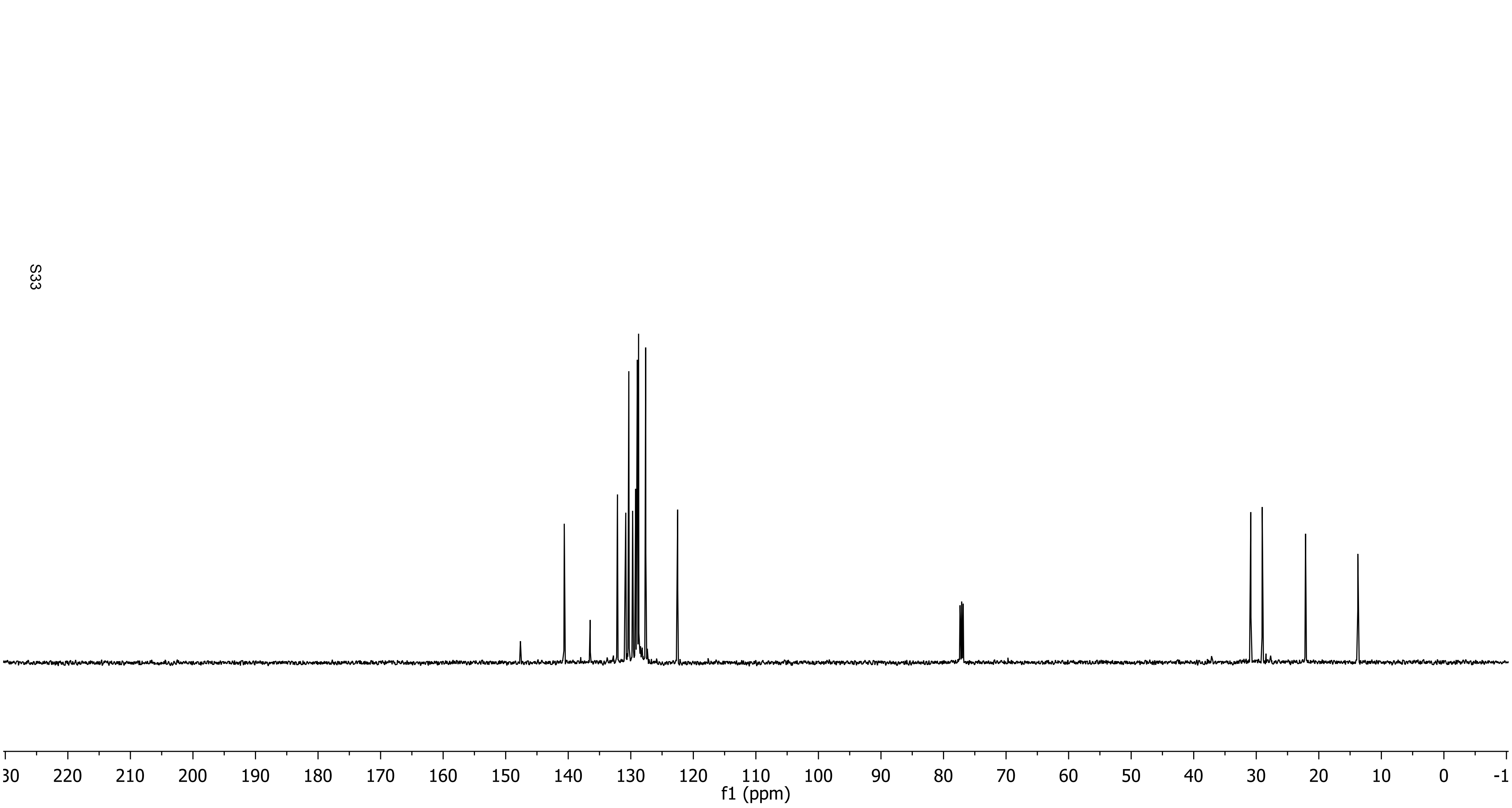

147.65
140.64
136.49
132.13
130.81
129.70
129.22
128.95
128.75
127.61
122.51
77.10
30.87
29.05
22.12
13.76
30
220
210
200
190
180
170
160
150
140
130
120
110
100
90
80
70
60
50
40
30
20
10
0
-1
f1 (ppm)

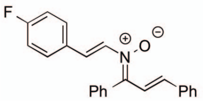

8ae

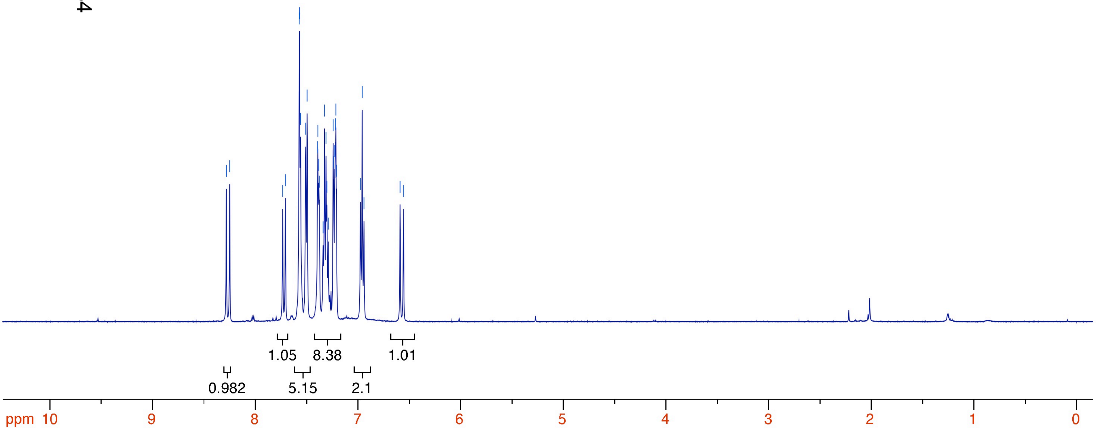
8.282
8.249
7.733
7.706
7.572
7.569
7.560
7.559
7.509
7.495
7.393
7.390
7.382
7.376
7.339
7.326
7.311
7.302
7.289
7.241
7.238
7.226
7.219
7.215
7.209
6.976
6.958
6.941
6.589
6.557
0.982
1.05
5.15
8.38
2.1
1.01
ppm 10
9
8
7
6
5
4
3
2
1
0

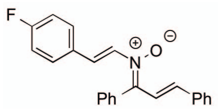

8ae

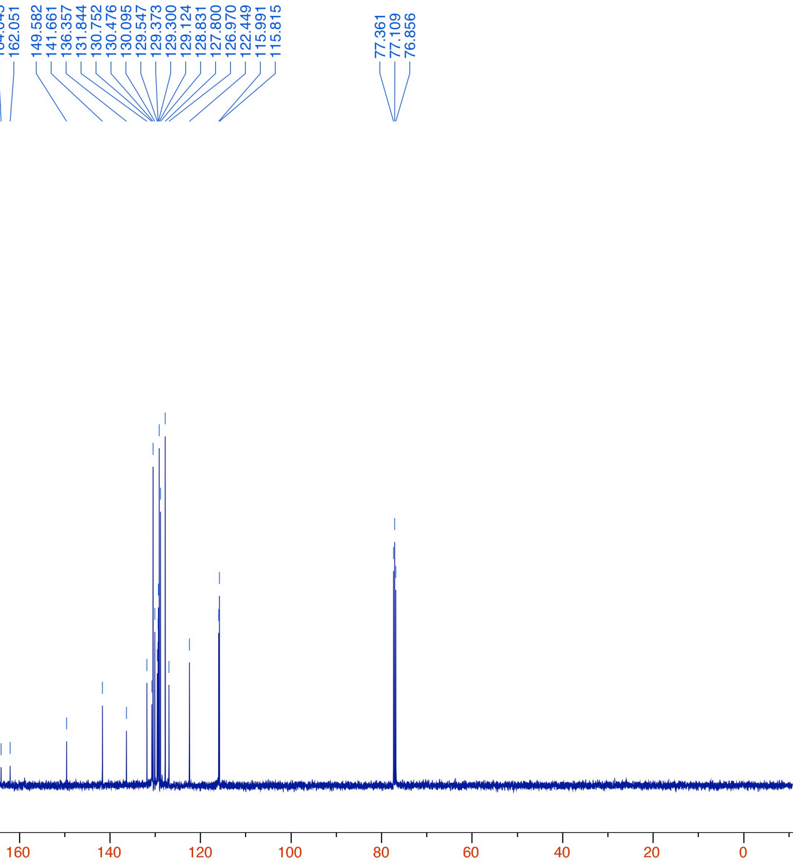
164.045
162.051
149.582
141.661
136.357
131.844
130.752
130.476
130.095
129.547
129.373
129.300
129.124
128.831
127.800
126.970
122.449
115.991
115.815
77.361
77.109
76.856
ppm 220
200
180
160
140
120
100
80
60
40
20
0

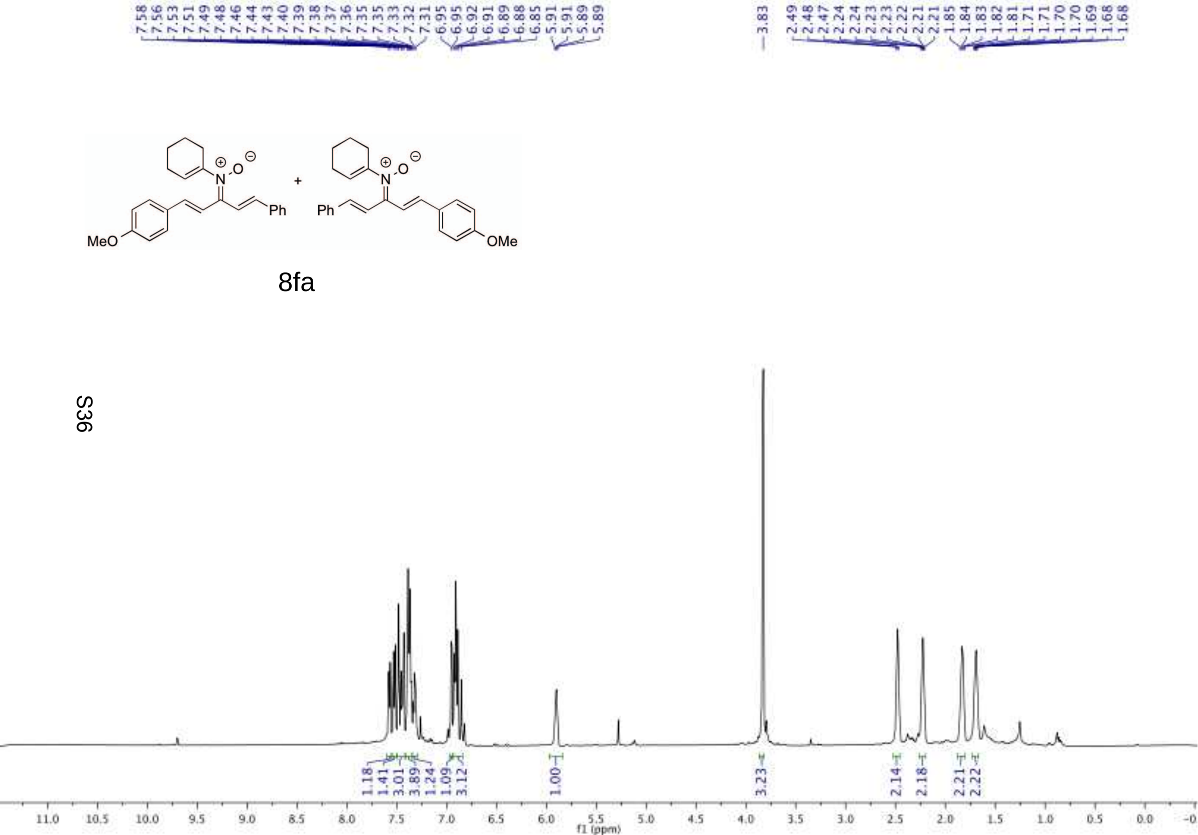
7.58 7.56 7.53 7.51 7.49 7.48 7.46 7.44 7.43 7.40 7.39 7.38 7.37 7.36 7.35 7.35 7.33 7.32 7.31 6.95 6.95 6.92 6.91 6.89 6.88 6.85 5.91 5.91 5.89 5.89
3.83
2.49 2.48 2.47 2.24 2.24 2.23 2.23 2.22 2.21 2.21 1.85 1.84 1.83 1.82 1.81 1.71 1.71 1.70 1.70 1.69 1.68 1.68
MeO
Ph
OMe
1.18 1.41 3.01 3.89 1.24 1.09 3.12 1.00 3.23 2.14 2.18 2.21 2.22
11.0 10.5 10.0 9.5 9.0 8.5 8.0 7.5 7.0 6.5 6.0 5.5 5.0 4.5 4.0 3.5 3.0 2.5 2.0 1.5 1.0 0.5 0.0
f1 (ppm)

8fa

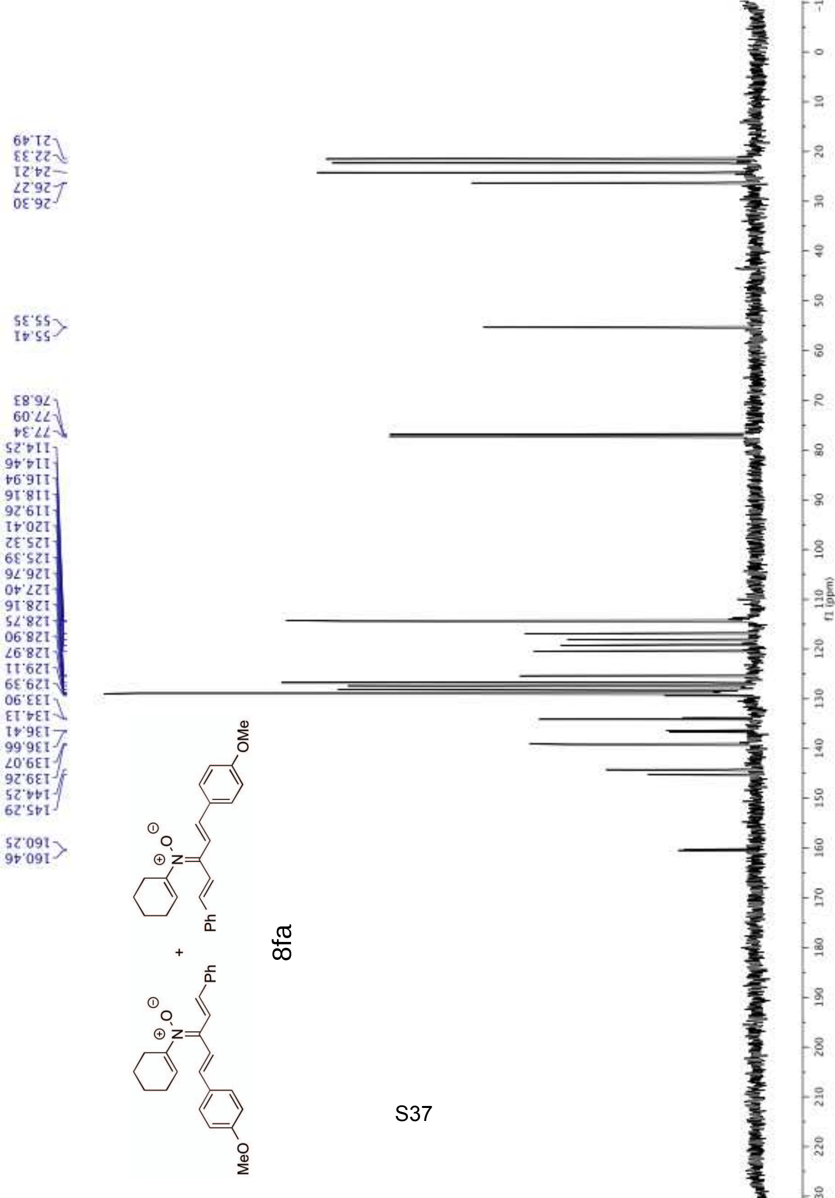
21.49
22.33
24.21
26.27
26.30
55.35
55.41
76.83
77.09
77.34
114.25
114.46
116.94
118.16
119.26
120.41
125.32
125.39
126.76
127.40
128.16
128.75
128.90
128.97
129.11
129.39
133.90
134.13
136.41
136.66
139.07
139.26
144.25
145.29
160.25
160.46
f1 (ppm)
MeO
Ph
OMe
+

8fa

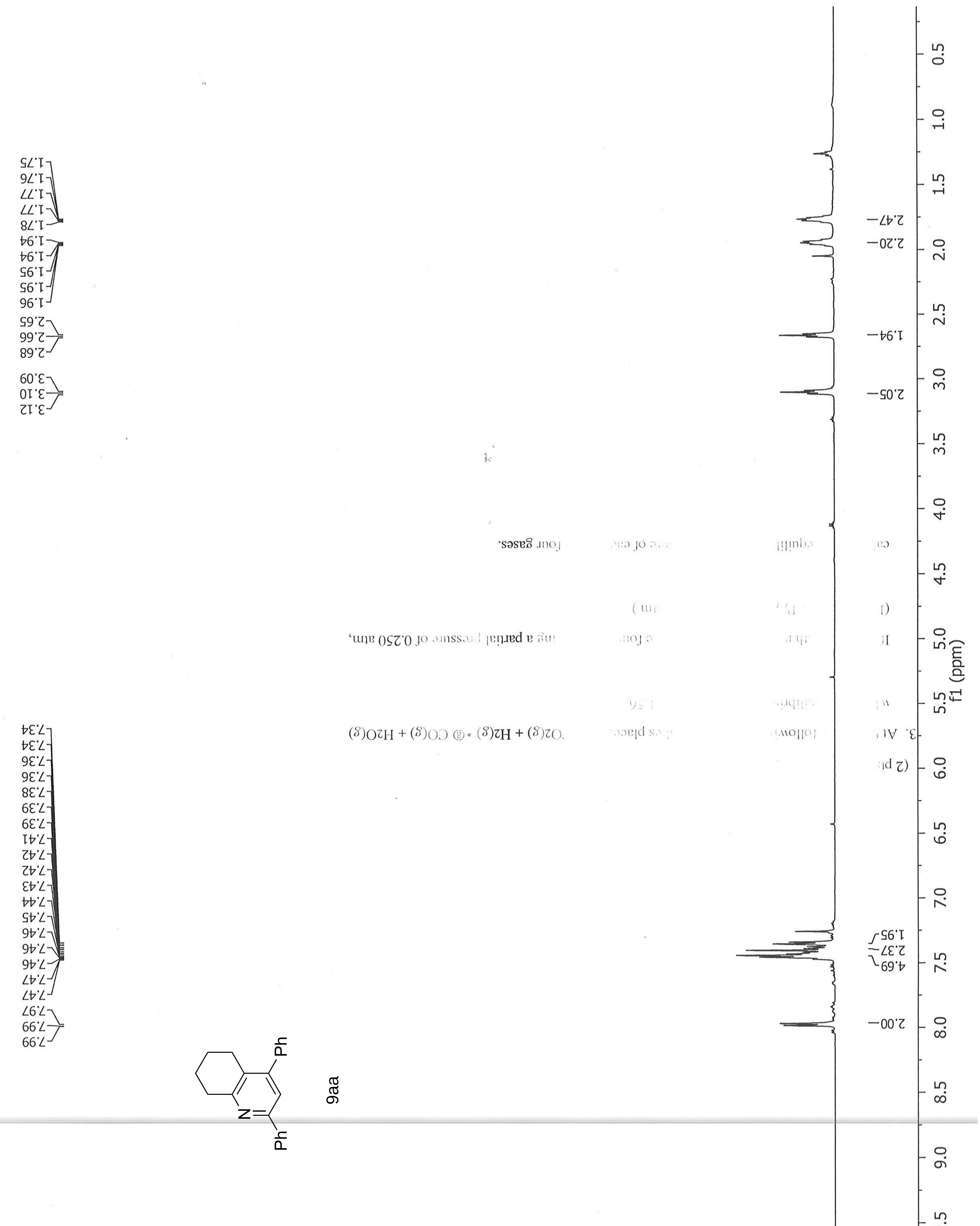
9aa
Ph
N
Ph
f1 (ppm)
2.00
4.69
2.37
1.95
2.05
1.94
2.20
2.47

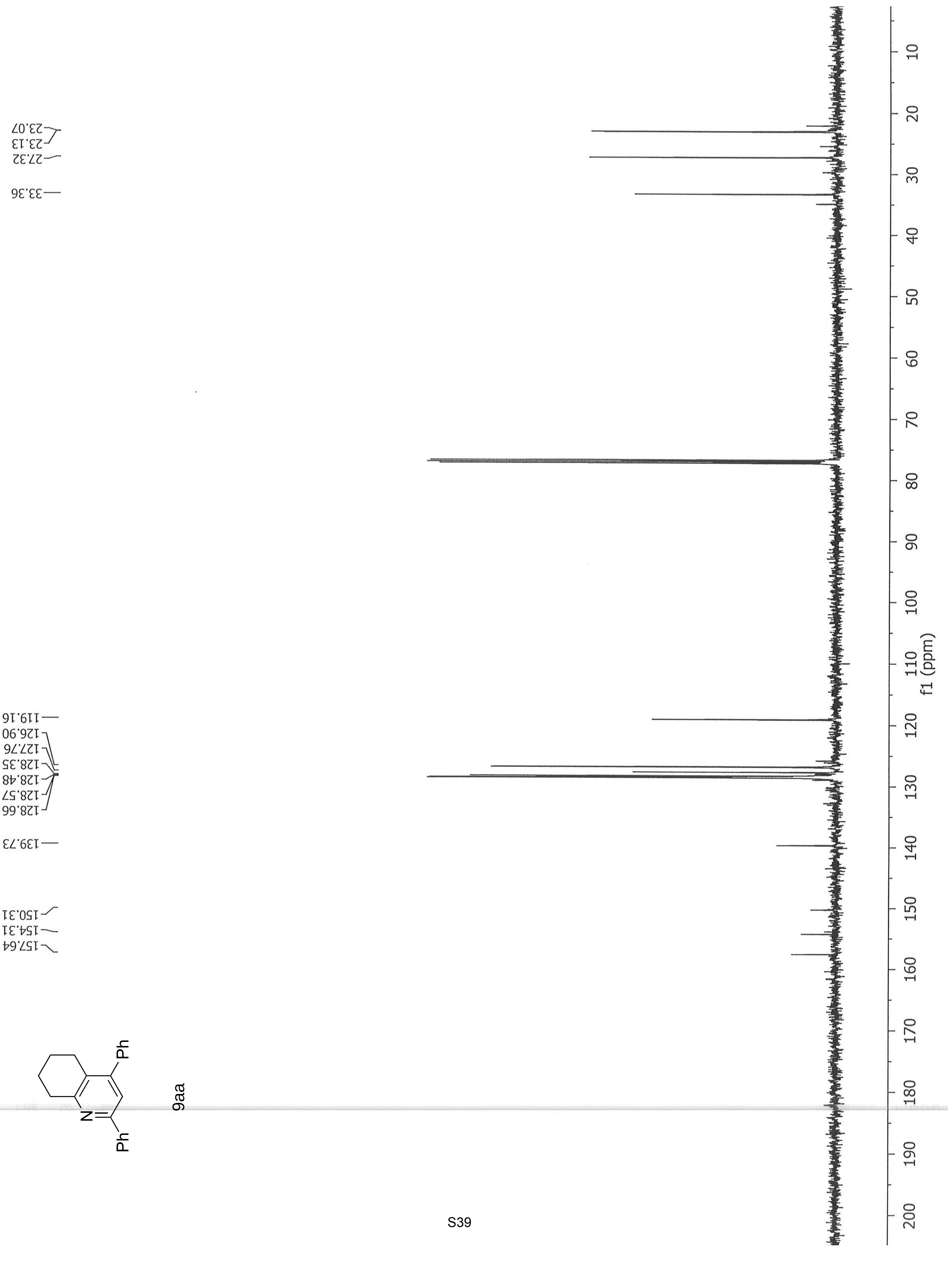
23.07
23.13
27.32
33.36
119.16
126.90
127.76
128.35
128.48
128.57
128.66
139.73
150.31
154.31
157.64
Ph
N
Ph
9aa
f1 (ppm)

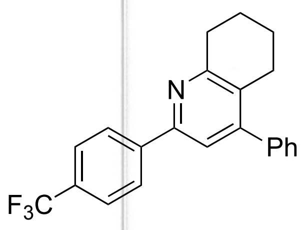
N
Ph
F3C

9ba

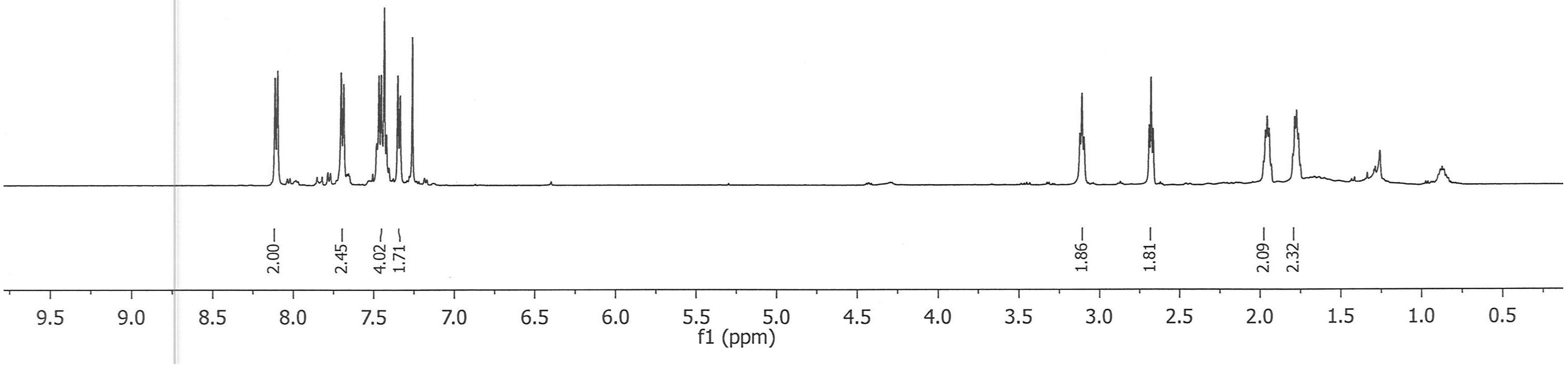
8.11
8.10
7.70
7.69
7.48
7.47
7.46
7.45
7.43
7.42
7.35
7.35
7.34
3.12
3.11
3.10
2.69
2.68
2.66
1.97
1.96
1.95
1.95
1.94
1.78
1.78
1.77
1.77
1.76
2.00
2.45
4.02
1.71
1.86
1.81
2.09
2.32
9.5
9.0
8.5
8.0
7.5
7.0
6.5
6.0
5.5
5.0
4.5
4.0
3.5
3.0
2.5
2.0
1.5
1.0
0.5
f1 (ppm)

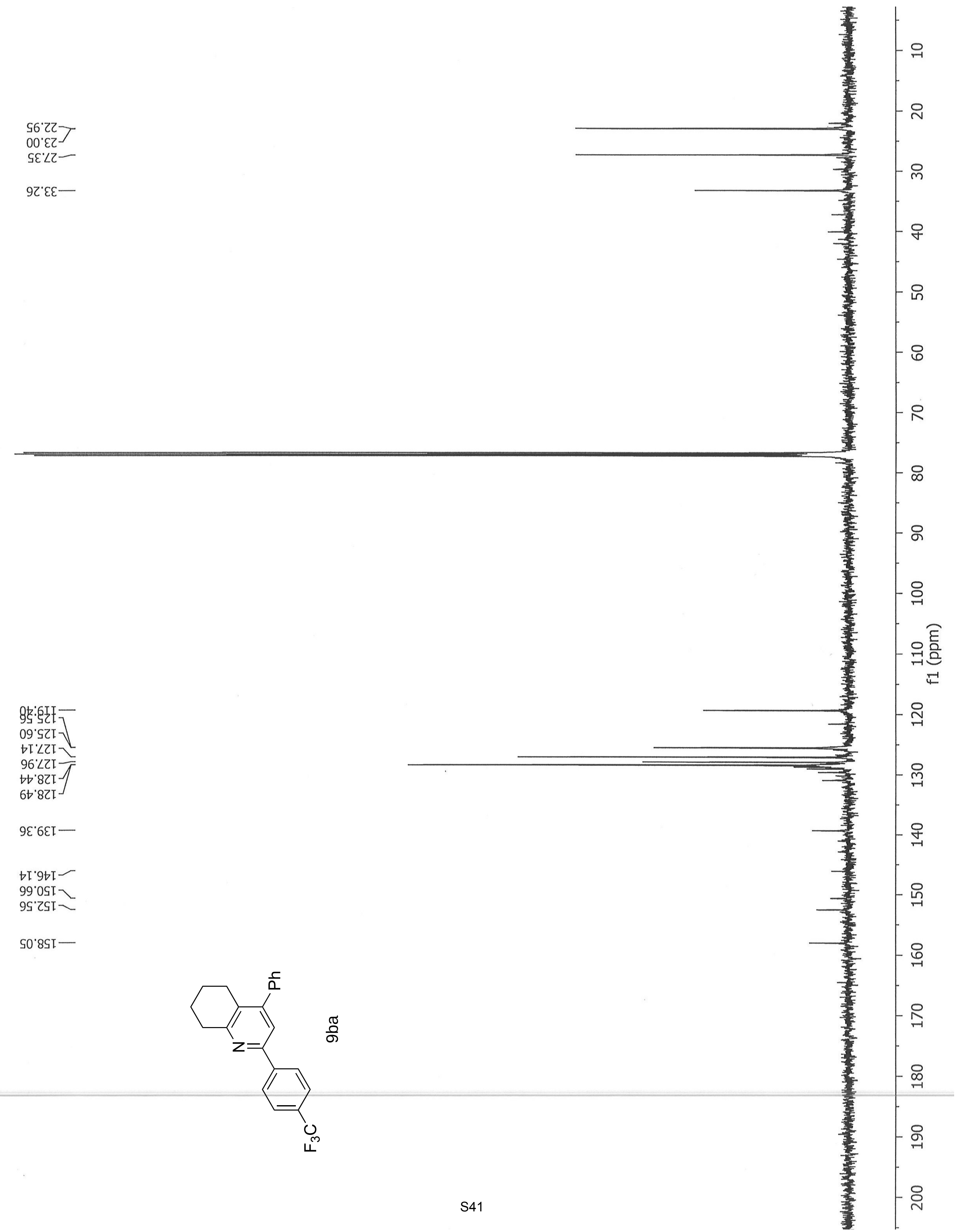

22.95
23.00
27.35
33.26
119.40
125.56
125.60
127.14
127.96
128.44
128.49
139.36
146.14
150.66
152.56
158.05
Ph
N
F_3C
9ba
f1 (ppm)

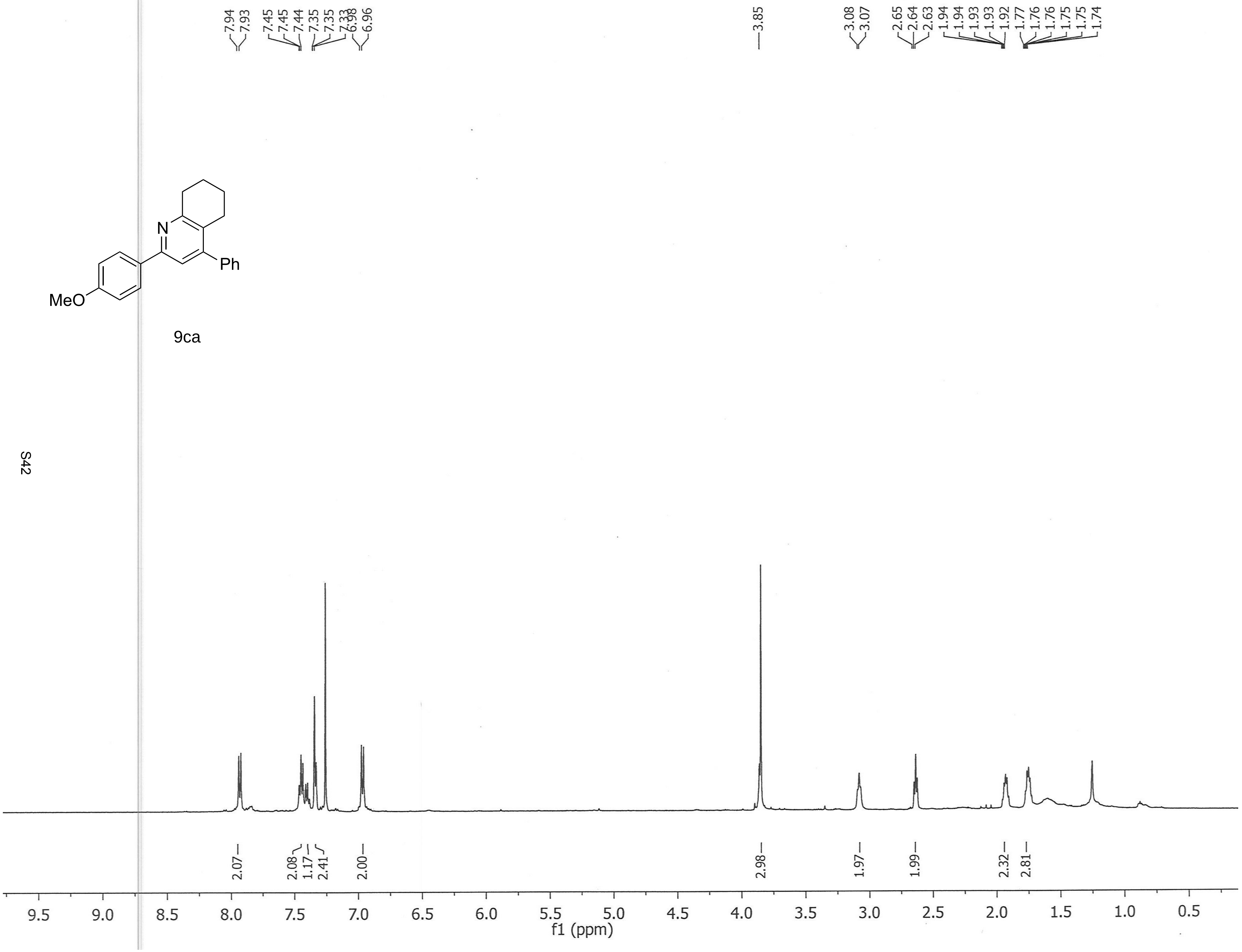
MeO
N
Ph
7.94
7.93
7.45
7.45
7.44
7.35
7.35
7.33
6.98
6.96
3.85
3.08
3.07
2.65
2.64
2.63
1.94
1.94
1.93
1.93
1.92
1.77
1.76
1.76
1.75
1.75
1.74
2.07
2.08
1.17
2.41
2.00
2.98
1.97
1.99
2.32
2.81
9.5
9.0
8.5
8.0
7.5
7.0
6.5
6.0
5.5
5.0
4.5
4.0
3.5
3.0
2.5
2.0
1.5
1.0
0.5
f1 (ppm)

9ca

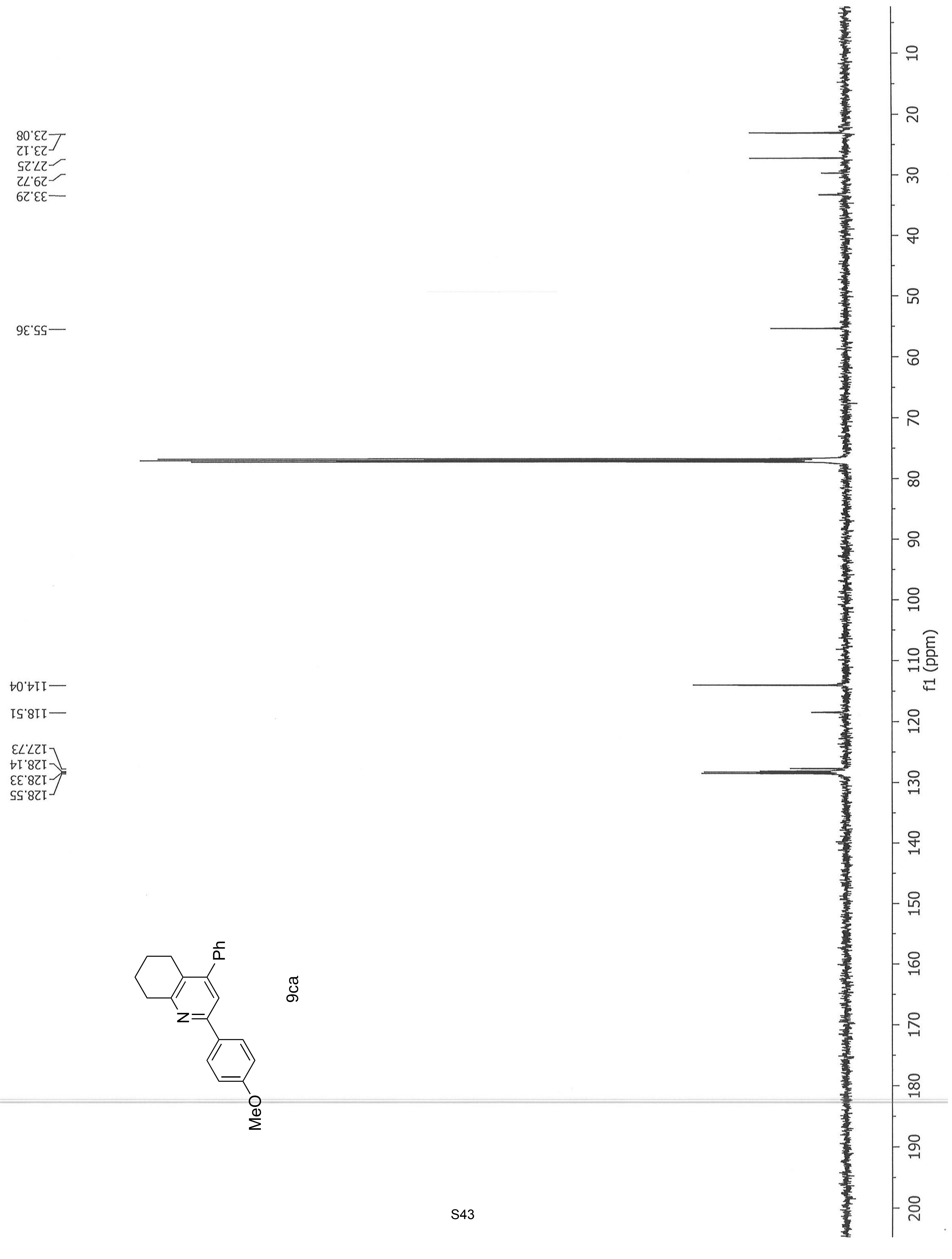
23.08
23.12
27.25
29.72
33.29
55.36
114.04
118.51
127.73
128.14
128.33
128.55
Ph
N
MeO
9ca
f1 (ppm)
10
20
30
40
50
60
70
80
90
100
110
120
130
140
150
160
170
180
190
200

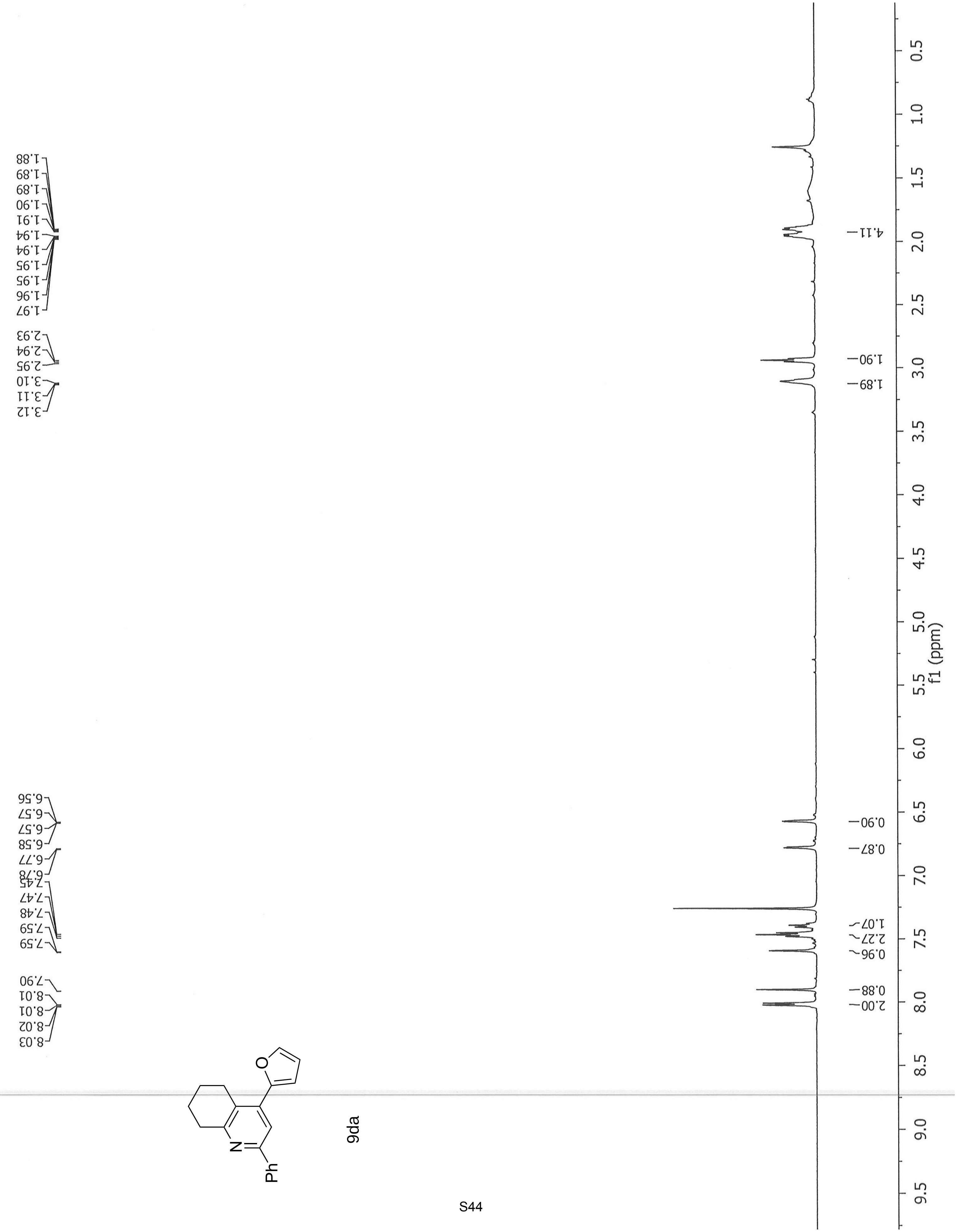
1.88
1.89
1.89
1.90
1.91
1.94
1.94
1.95
1.95
1.96
1.97
2.93
2.94
2.95
3.10
3.11
3.12
6.56
6.57
6.57
6.58
6.77
6.78
7.45
7.47
7.48
7.59
7.59
7.90
8.01
8.01
8.02
8.03
Ph
N
O
4.11
1.90
1.89
0.90
0.87
1.07
2.27
0.96
0.88
2.00
f1 (ppm)

9da

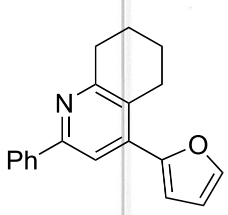

9da

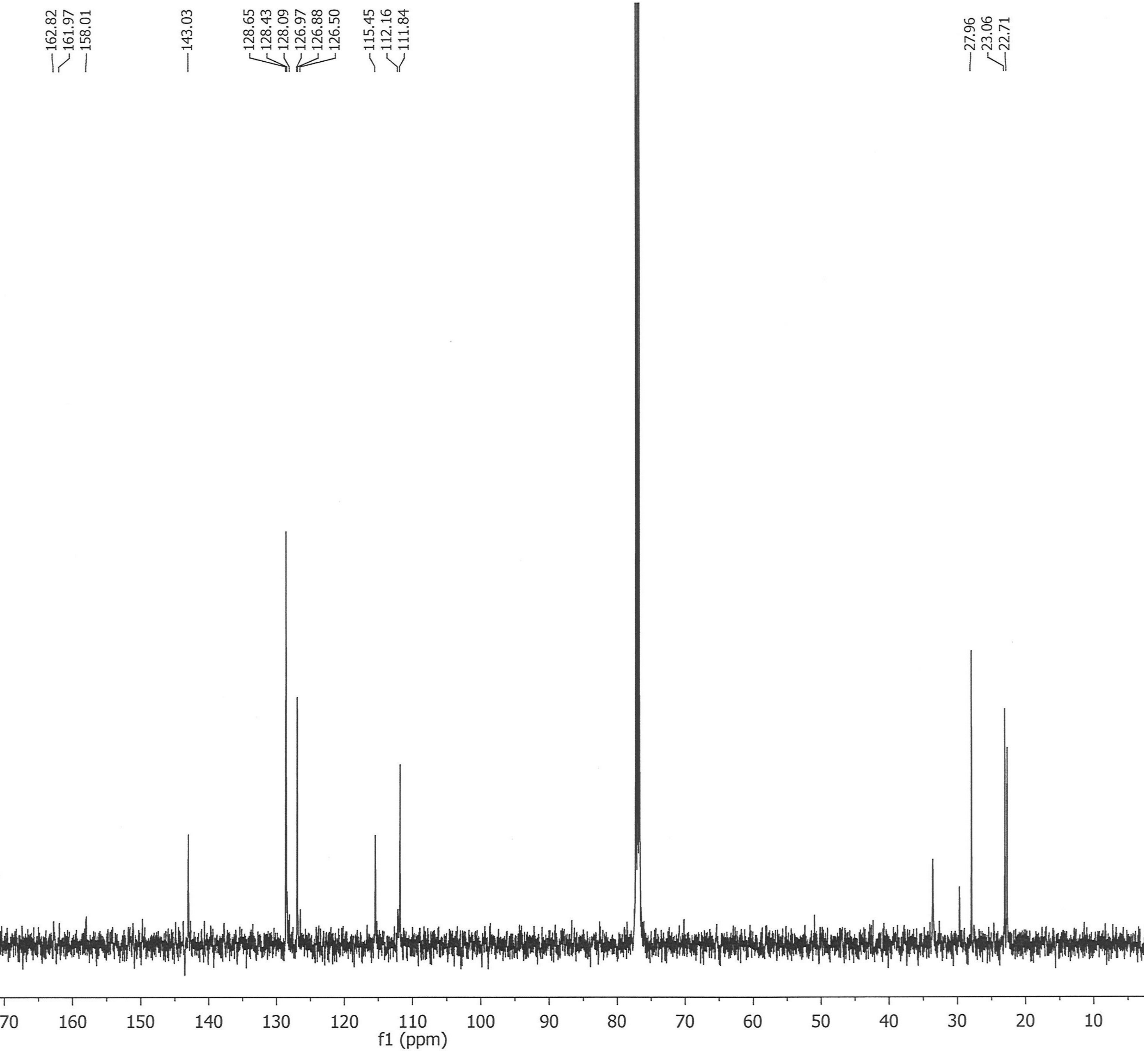
162.82
161.97
158.01
143.03
128.65
128.43
128.09
126.97
126.88
126.50
115.45
112.16
111.84
27.96
23.06
22.71
200
190
180
170
160
150
140
130
120
110
100
90
80
70
60
50
40
30
20
10
f1 (ppm)

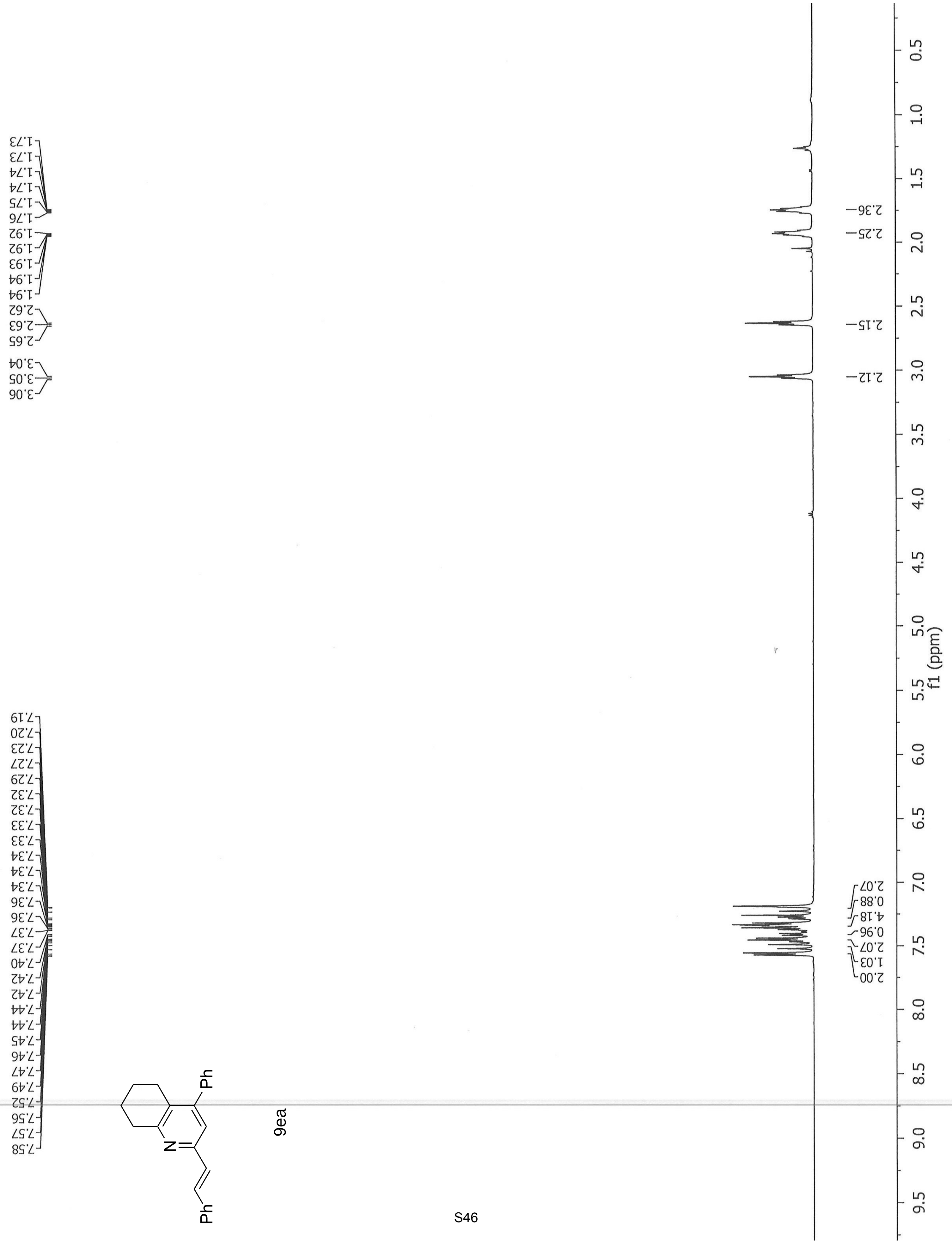
1.73
1.73
1.74
1.74
1.75
1.76
1.92
1.92
1.93
1.94
1.94
2.62
2.63
2.65
3.04
3.05
3.06
7.19
7.20
7.23
7.27
7.29
7.32
7.32
7.33
7.33
7.34
7.34
7.34
7.36
7.36
7.37
7.37
7.40
7.42
7.42
7.44
7.44
7.45
7.46
7.47
7.49
7.52
7.56
7.57
7.58
Ph
N
Ph
2.36
2.25
2.15
2.12
2.07
0.88
4.18
0.96
2.07
1.03
2.00
f1 (ppm)

9ea

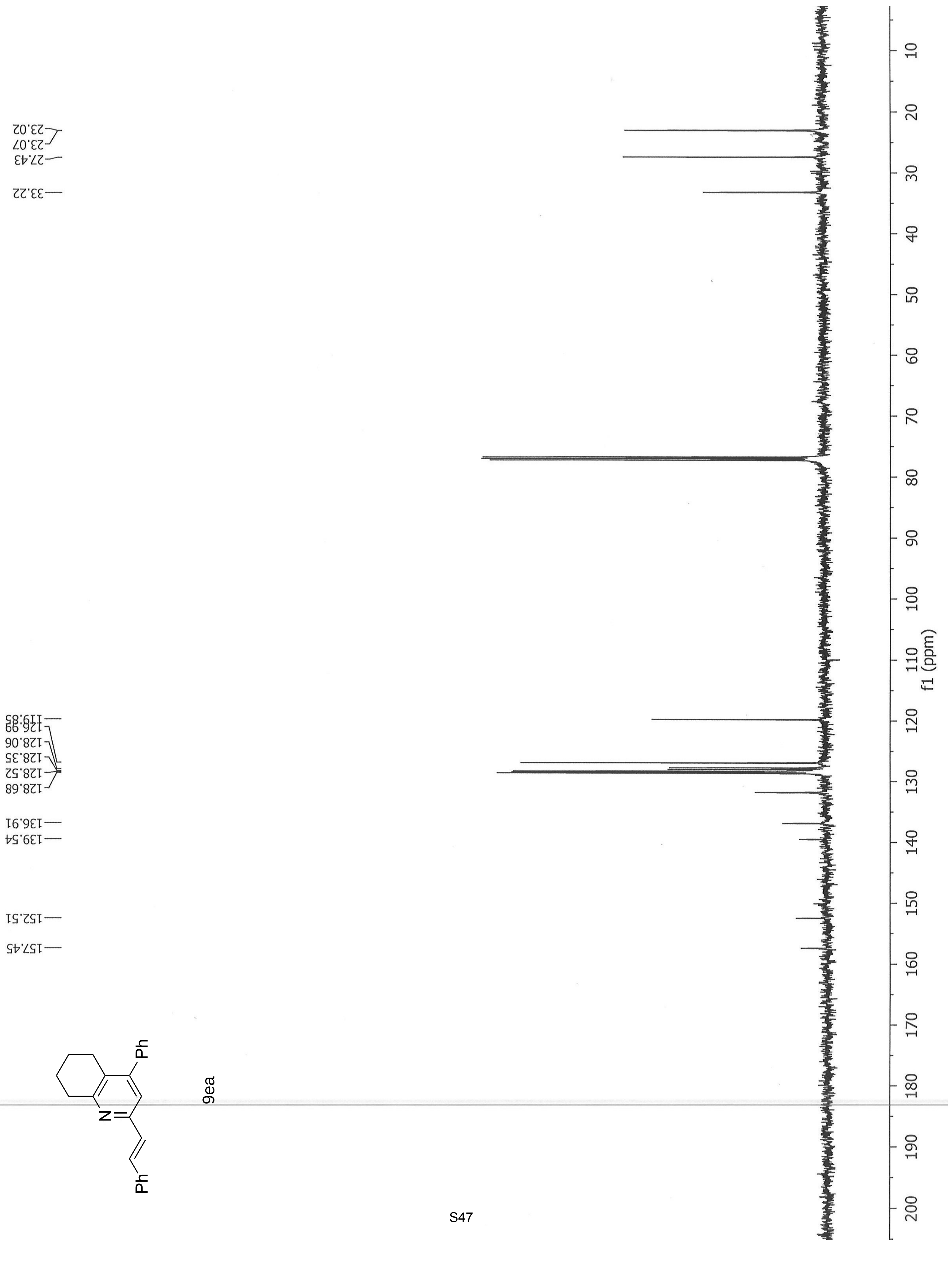
23.02
23.07
27.43
33.22
119.85
126.99
128.06
128.35
128.52
128.68
136.91
139.54
152.51
157.45
Ph
N
Ph
9ea
f1 (ppm)
10
20
30
40
50
60
70
80
90
100
110
120
130
140
150
160
170
180
190
200

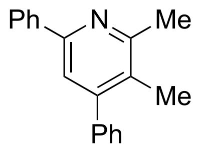

9ab

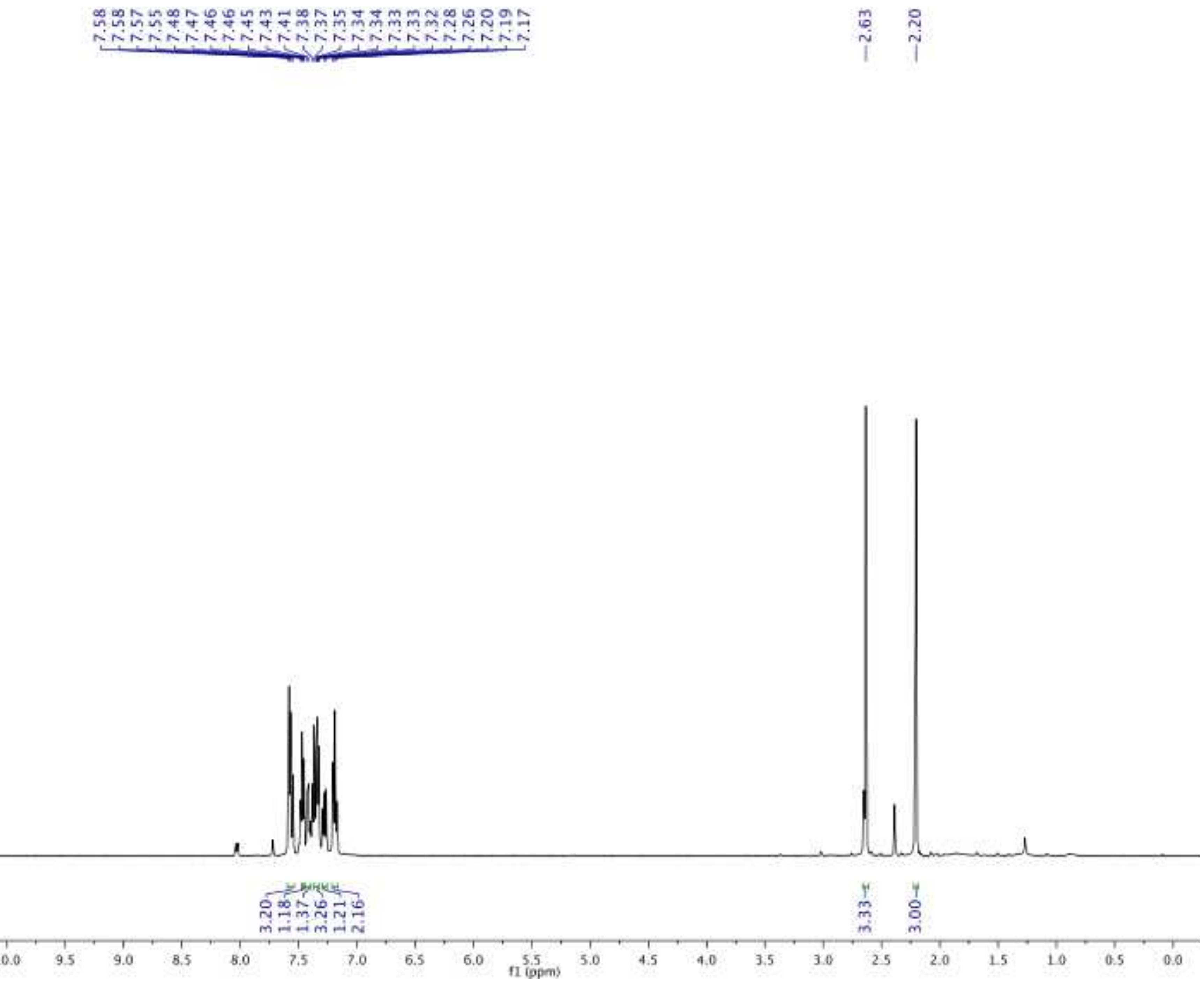

7.58
7.58
7.57
7.55
7.48
7.47
7.46
7.46
7.45
7.43
7.41
7.38
7.37
7.35
7.34
7.34
7.33
7.33
7.32
7.28
7.26
7.20
7.19
7.17
2.63
2.20
3.20
1.18
1.37
3.26
1.21
2.16
3.33
3.00
11.0
10.5
10.0
9.5
9.0
8.5
8.0
7.5
7.0
6.5
6.0
5.5
5.0
4.5
4.0
3.5
3.0
2.5
2.0
1.5
1.0
0.5
0.0
f1 (ppm)

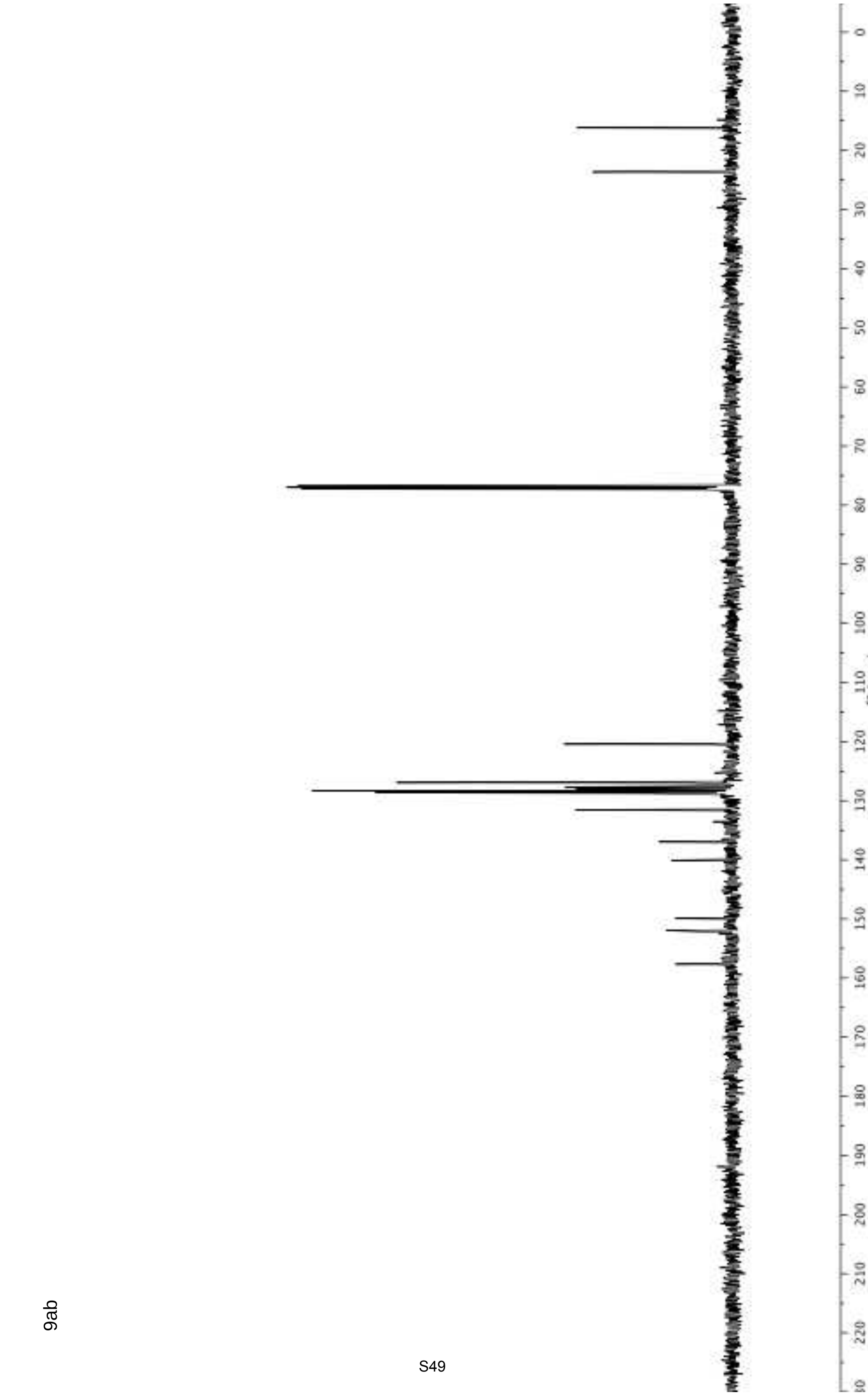

16.19
23.64
76.76
77.02
77.27
120.44
126.94
127.69
127.96
128.33
128.64
128.73
131.53
136.97
140.06
149.93
151.93
157.66
f1 (ppm)

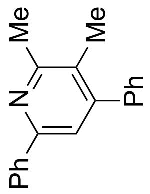

Me
Me
N
Ph
Ph

9ab

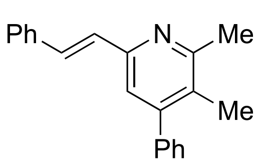

9eb

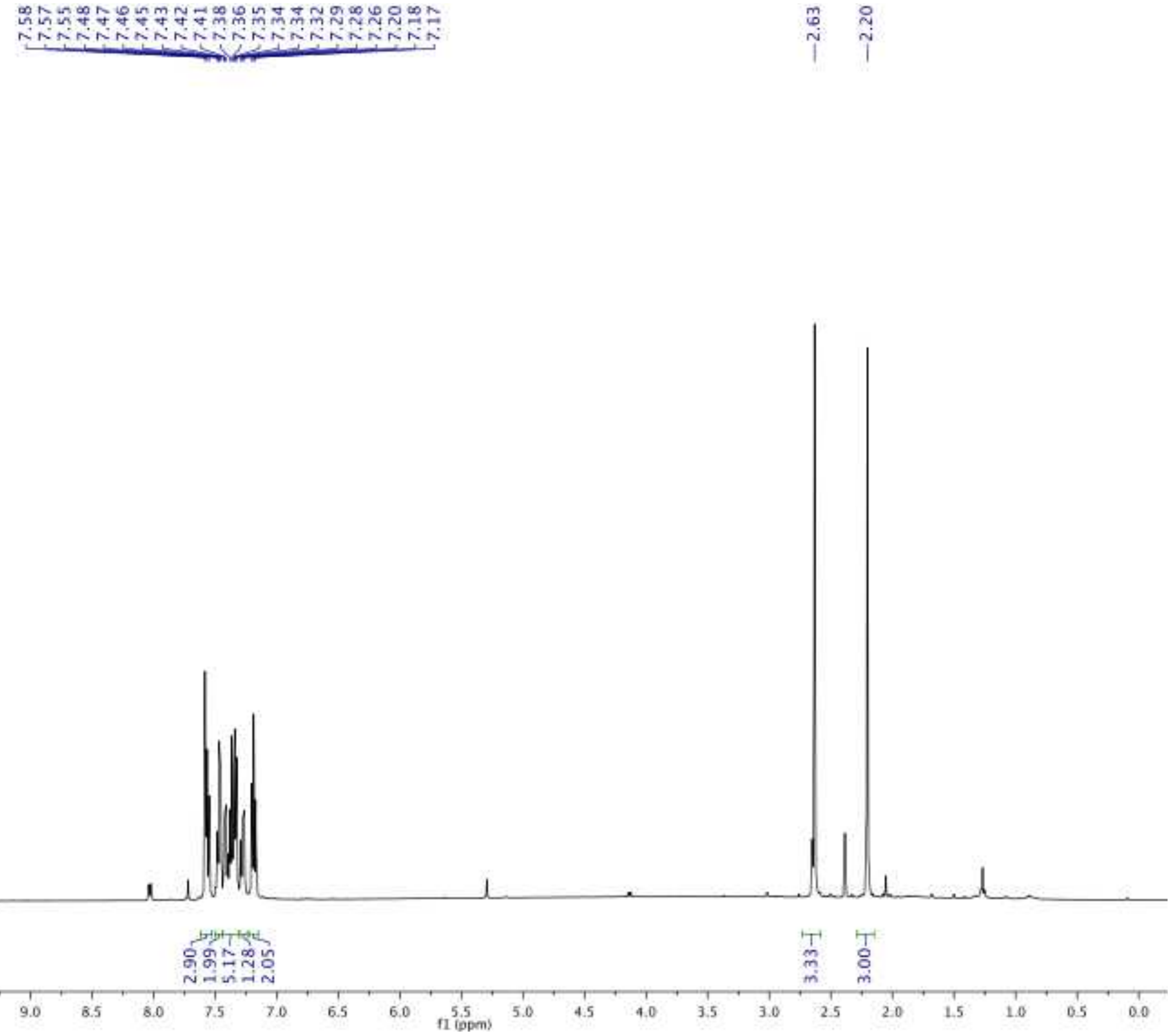
7.58
7.57
7.55
7.48
7.47
7.46
7.45
7.43
7.42
7.41
7.38
7.36
7.35
7.34
7.34
7.32
7.29
7.28
7.26
7.20
7.18
7.17
2.63
2.20
2.90
1.99
5.17
1.28
2.05
3.33
3.00
11.0 10.5 10.0 9.5 9.0 8.5 8.0 7.5 7.0 6.5 6.0 5.5 5.0 4.5 4.0 3.5 3.0 2.5 2.0 1.5 1.0 0.5 0.0
f1 (ppm)

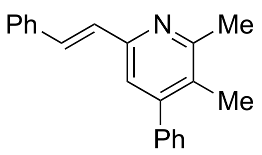

9eb

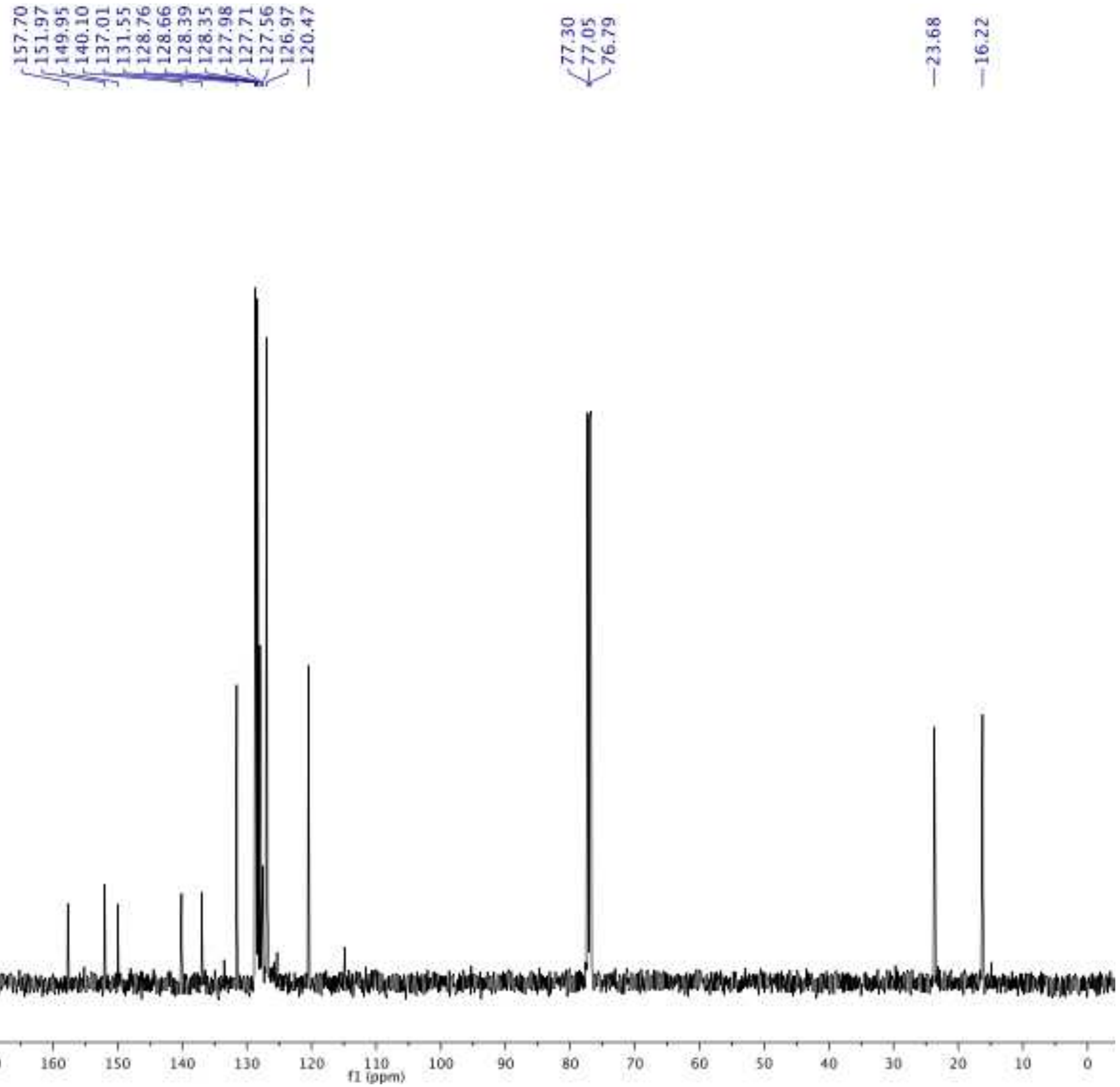
157.70
151.97
149.95
140.10
137.01
131.55
128.76
128.66
128.39
128.35
127.98
127.71
127.56
126.97
120.47
77.30
77.05
76.79
23.68
16.22
f1 (ppm)

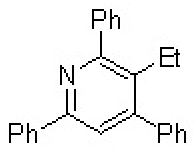

9ac

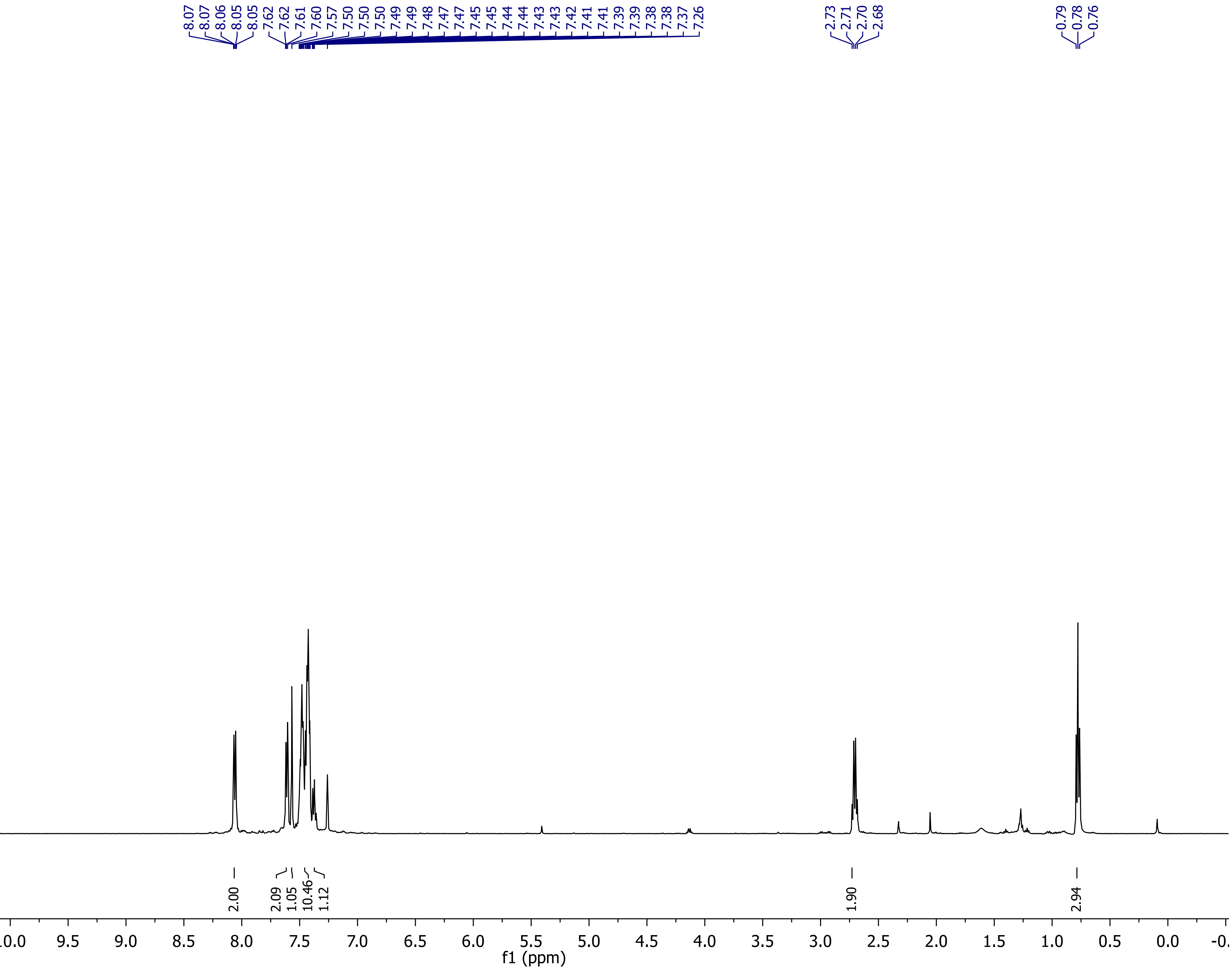

8.07
8.07
8.06
8.05
8.05
7.62
7.62
7.61
7.60
7.57
7.50
7.50
7.50
7.49
7.49
7.48
7.47
7.47
7.45
7.45
7.44
7.44
7.43
7.43
7.42
7.41
7.41
7.39
7.39
7.38
7.38
7.37
7.26
2.73
2.71
2.70
2.68
0.79
0.78
0.76
2.00
2.09
1.05
10.46
1.12
1.90
2.94
f1 (ppm)

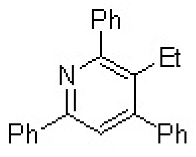

9ac

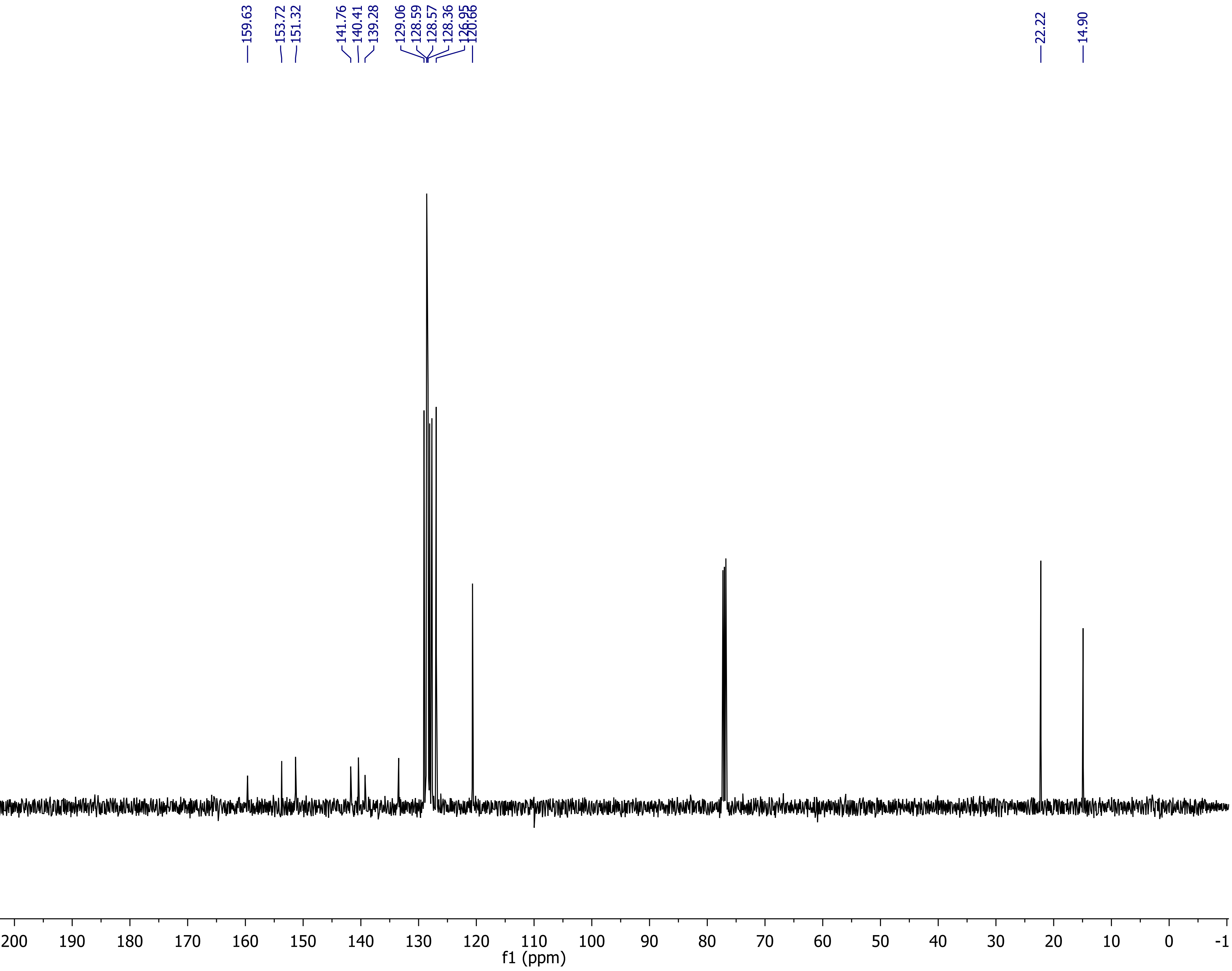

159.63
153.72
151.32
141.76
140.41
139.28
129.06
128.59
128.57
128.36
126.95
120.66
22.22
14.90
f1 (ppm)

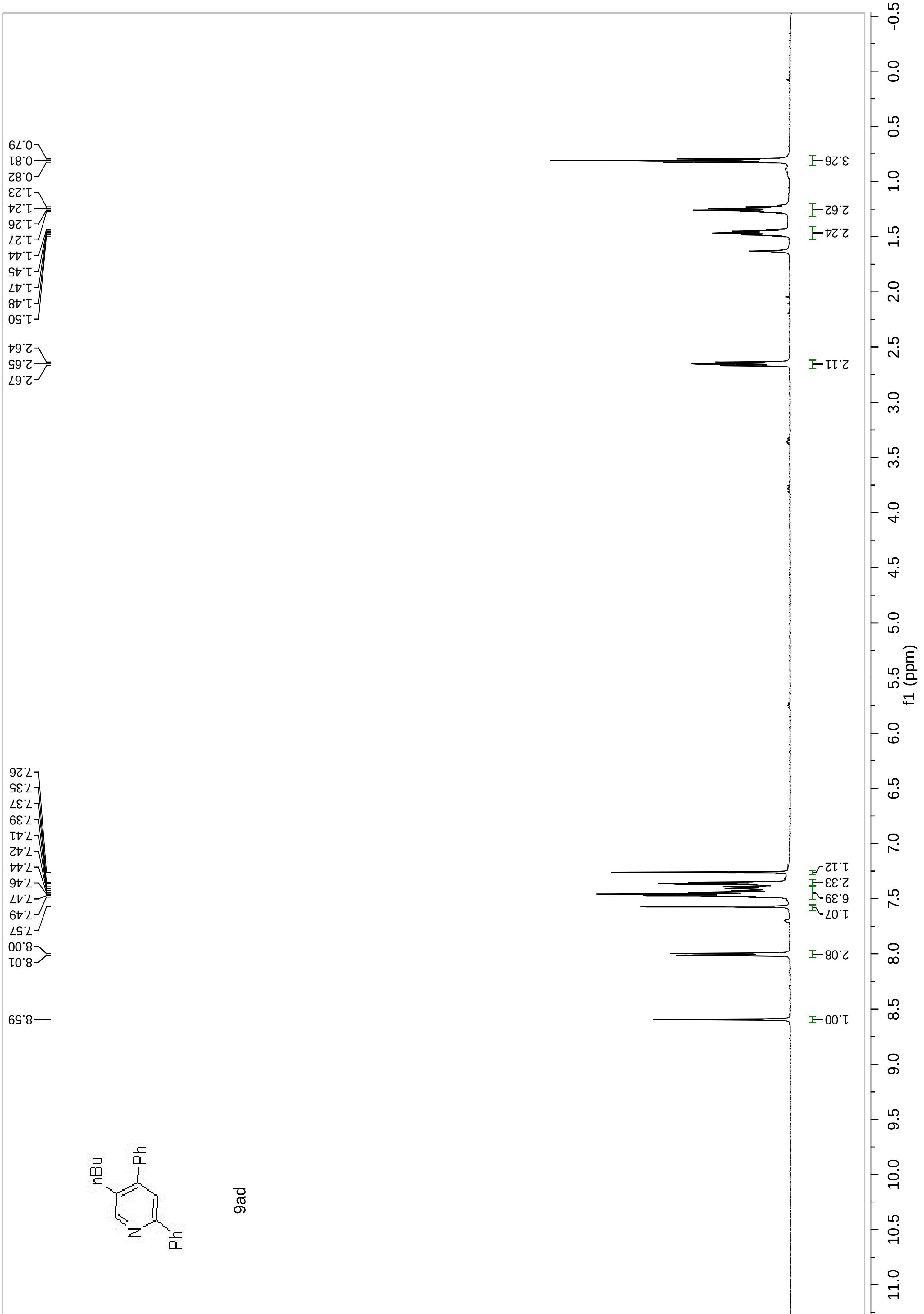
0.79
0.81
0.82
1.23
1.24
1.26
1.27
1.44
1.45
1.47
1.48
1.50
2.64
2.65
2.67
7.26
7.35
7.37
7.39
7.41
7.42
7.44
7.46
7.47
7.49
7.57
8.00
8.01
8.59
3.26
2.62
2.24
2.11
1.12
2.33
6.39
1.07
2.08
1.00
nBu
Ph
N
Ph
9ad
f1 (ppm)

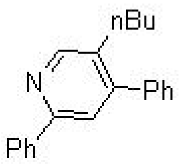
nBu
N
Ph
Ph

9ad

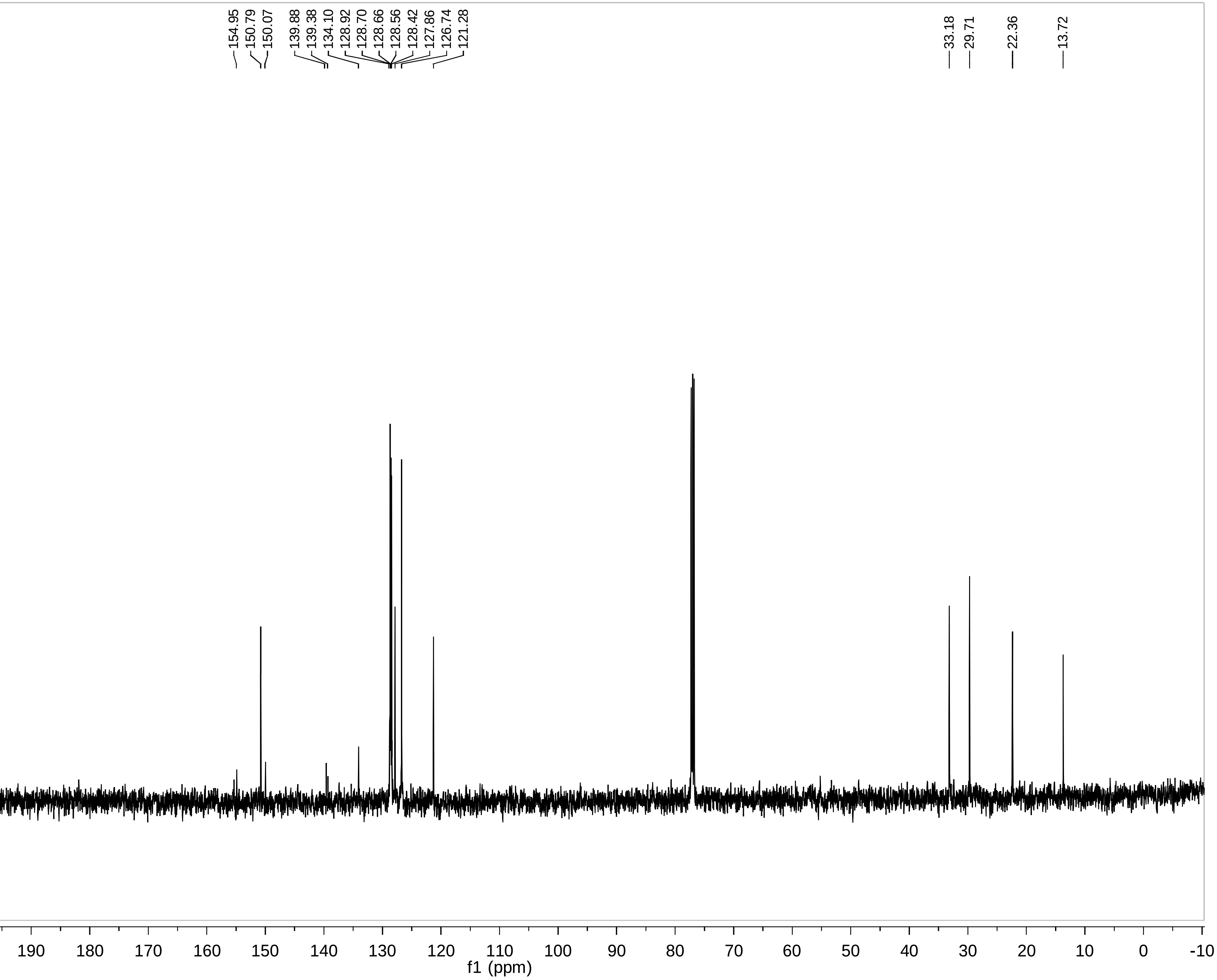
154.95
150.79
150.07
139.88
139.38
134.10
128.92
128.70
128.66
128.56
128.42
127.86
126.74
121.28
33.18
29.71
22.36
13.72
230 220 210 200 190 180 170 160 150 140 130 120 110 100 90 80 70 60 50 40 30 20 10 0 -10
f1 (ppm)

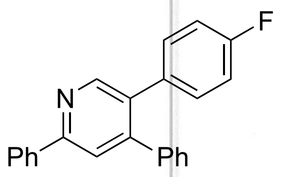
F
N
Ph
Ph

9ae

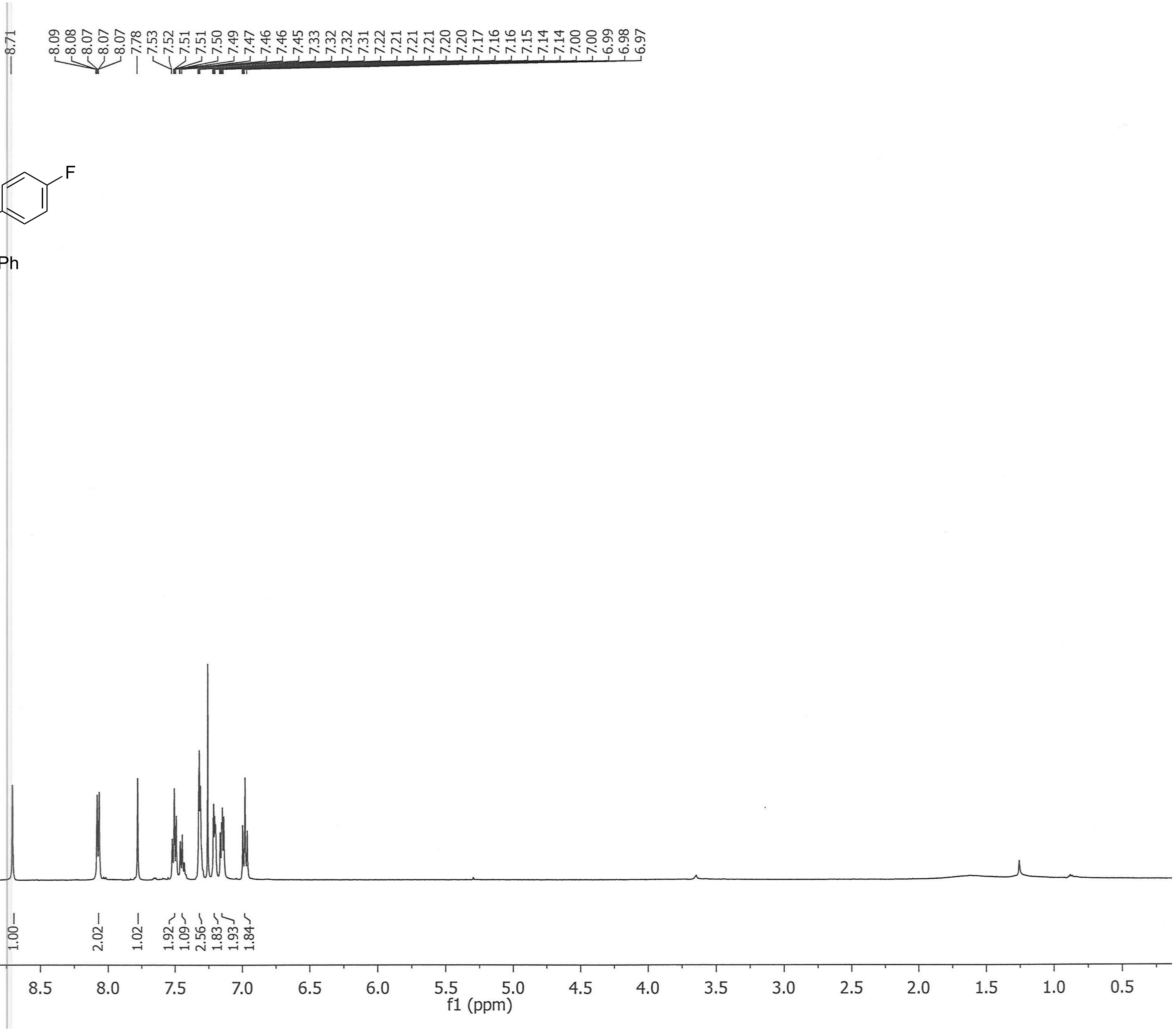
8.71
8.09
8.08
8.07
8.07
8.07
7.78
7.53
7.52
7.51
7.51
7.50
7.49
7.47
7.46
7.46
7.45
7.33
7.32
7.32
7.31
7.22
7.21
7.21
7.21
7.20
7.20
7.17
7.16
7.16
7.15
7.14
7.14
7.00
7.00
6.99
6.98
6.97
1.00
2.02
1.02
1.92
1.09
2.56
1.83
1.93
1.84
9.5
9.0
8.5
8.0
7.5
7.0
6.5
6.0
5.5
5.0
4.5
4.0
3.5
3.0
2.5
2.0
1.5
1.0
0.5
f1 (ppm)

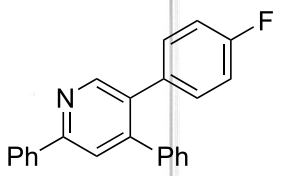
F
N
Ph
Ph

9ae

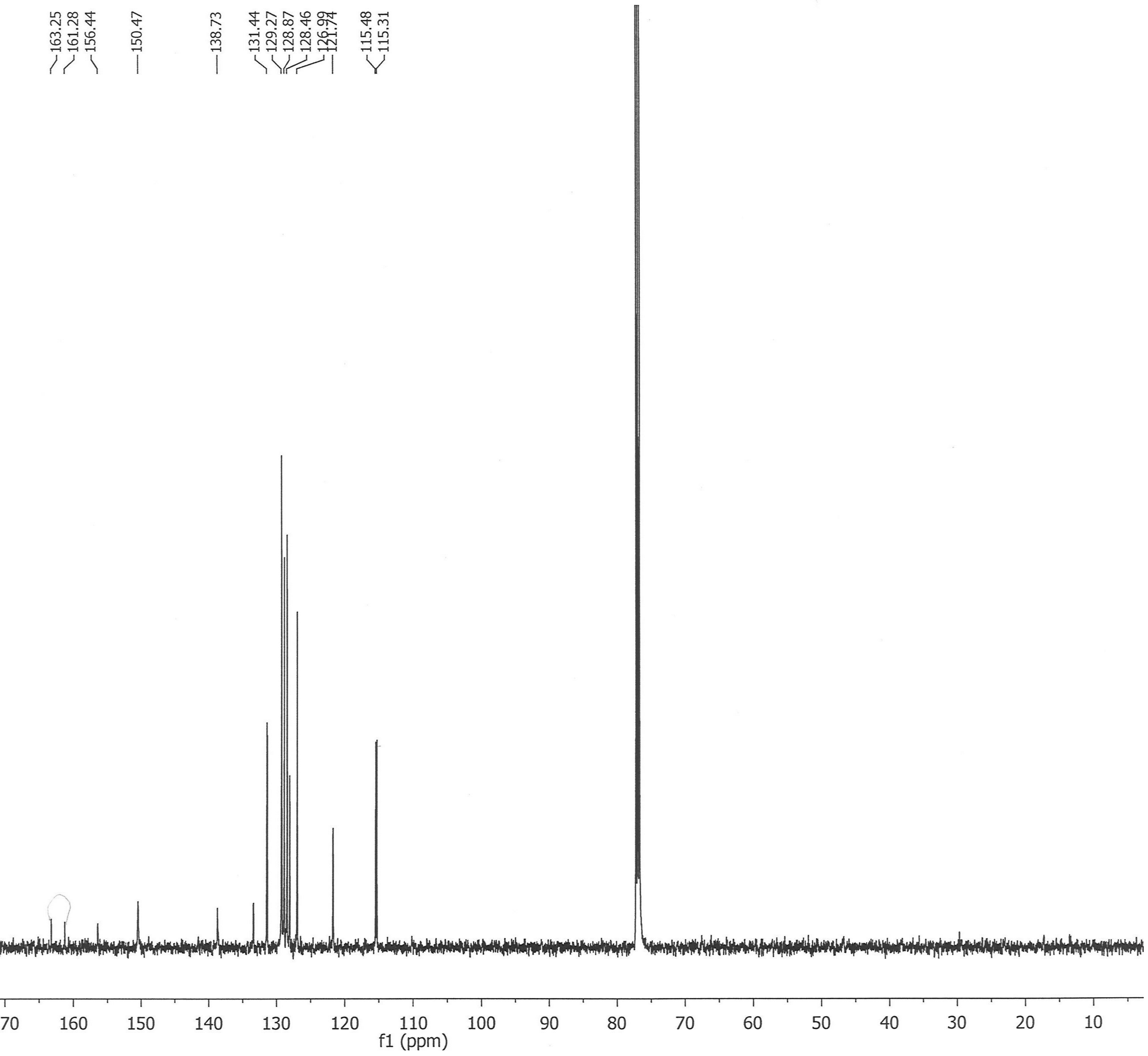
163.25
161.28
156.44
150.47
138.73
131.44
129.27
128.87
128.46
126.99
121.74
115.48
115.31
200
190
180
170
160
150
140
130
120
110
100
90
80
70
60
50
40
30
20
10
f1 (ppm)

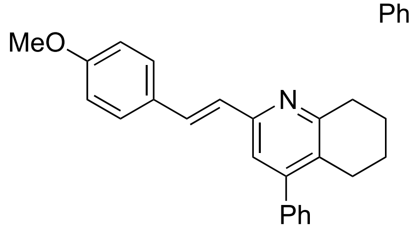

9fa

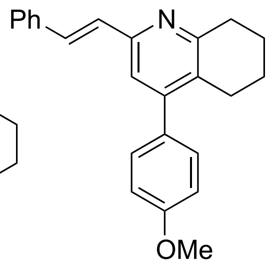

9fa'

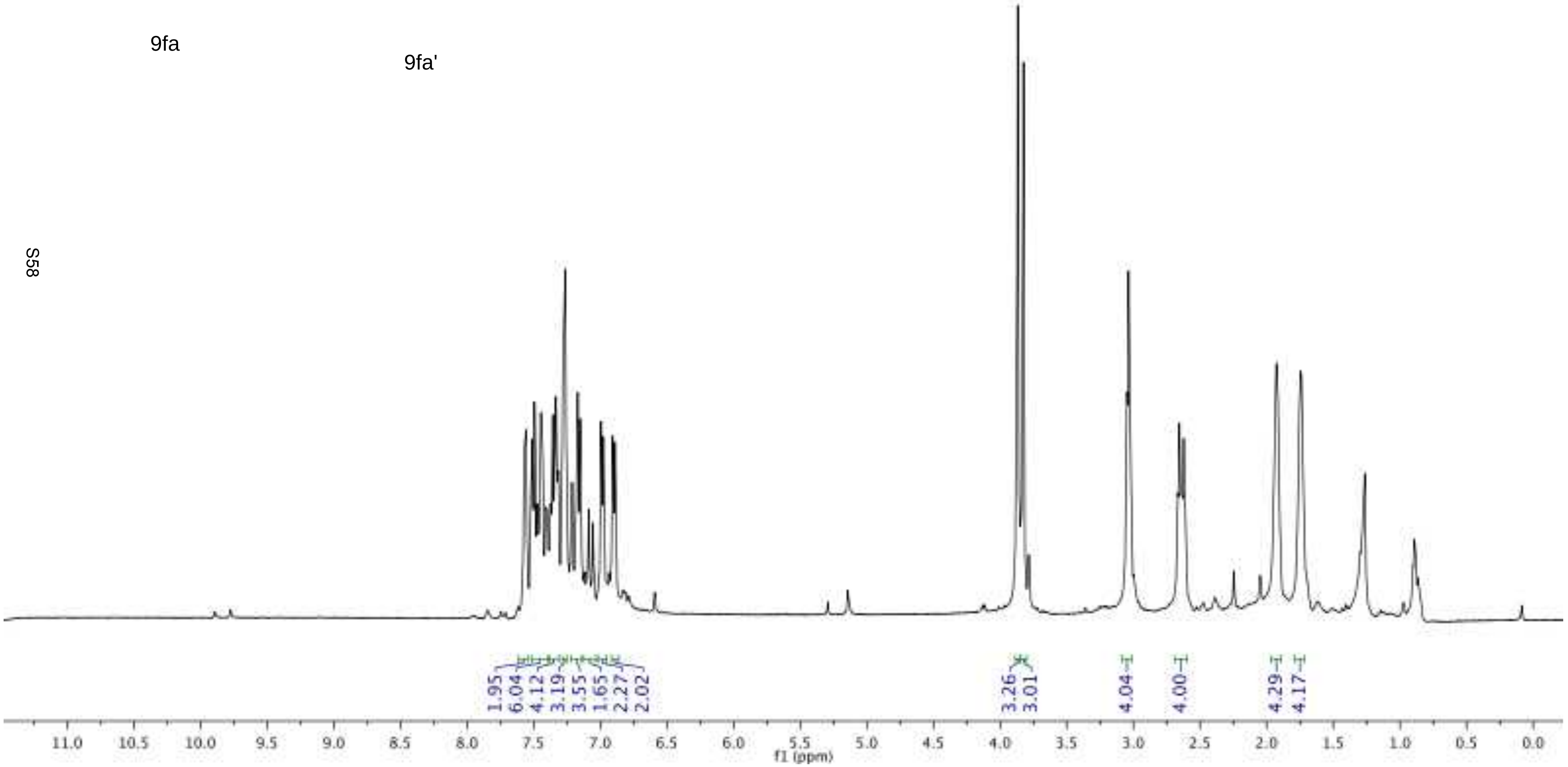
7.57 7.56 7.52 7.51 7.49 7.49 7.47 7.46 7.45 7.44 7.43 7.41 7.39 7.38 7.37 7.36 7.34 7.33 7.32 7.28 7.26 7.21 7.18 7.17 7.15 7.09 7.05 6.99 6.98 6.90 6.89
3.87 3.82
3.05 3.03 3.02 2.67 2.65 2.64 2.63 2.62 2.61 1.94 1.93 1.91 1.76 1.75 1.74 1.74 1.73 1.73
1.95 6.04 4.12 3.19 3.55 1.65 2.27 2.02
3.26 3.01
4.04
4.00
4.29 4.17
11.0 10.5 10.0 9.5 9.0 8.5 8.0 7.5 7.0 6.5 6.0 5.5 5.0 4.5 4.0 3.5 3.0 2.5 2.0 1.5 1.0 0.5 0.0
f1 (ppm)

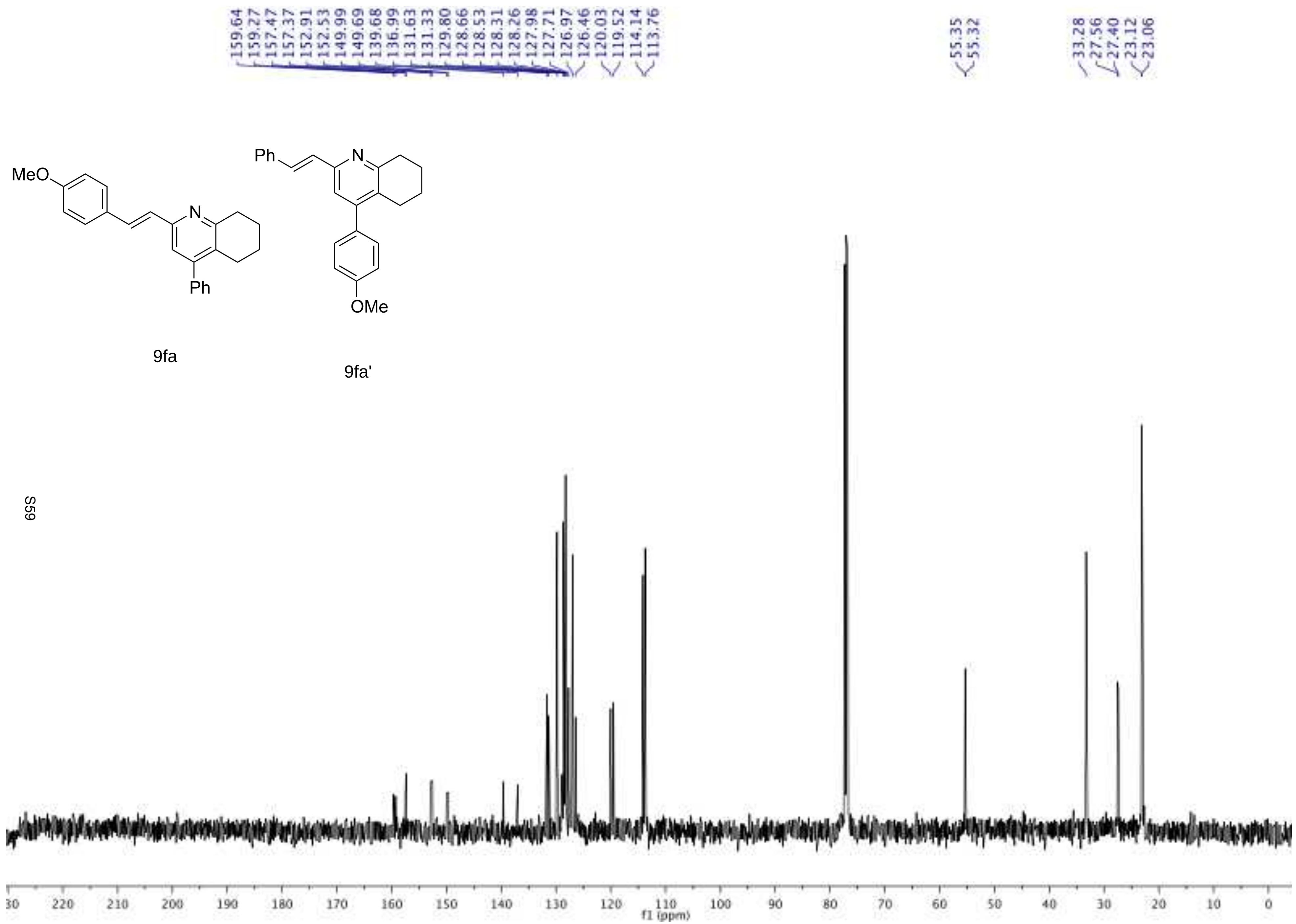
159.64
159.27
157.47
157.37
152.91
152.53
149.99
149.69
139.68
136.99
131.63
131.33
129.80
128.66
128.53
128.31
128.26
127.98
127.71
126.97
126.46
120.03
119.52
114.14
113.76
55.35
55.32
33.28
27.56
27.40
23.12
23.06
MeO
N
Ph
9fa
N
Ph
OMe
9fa'
230
220
210
200
190
180
170
160
150
140
130
120
110
f1 (ppm)
100
90
80
70
60
50
40
30
20
10
0

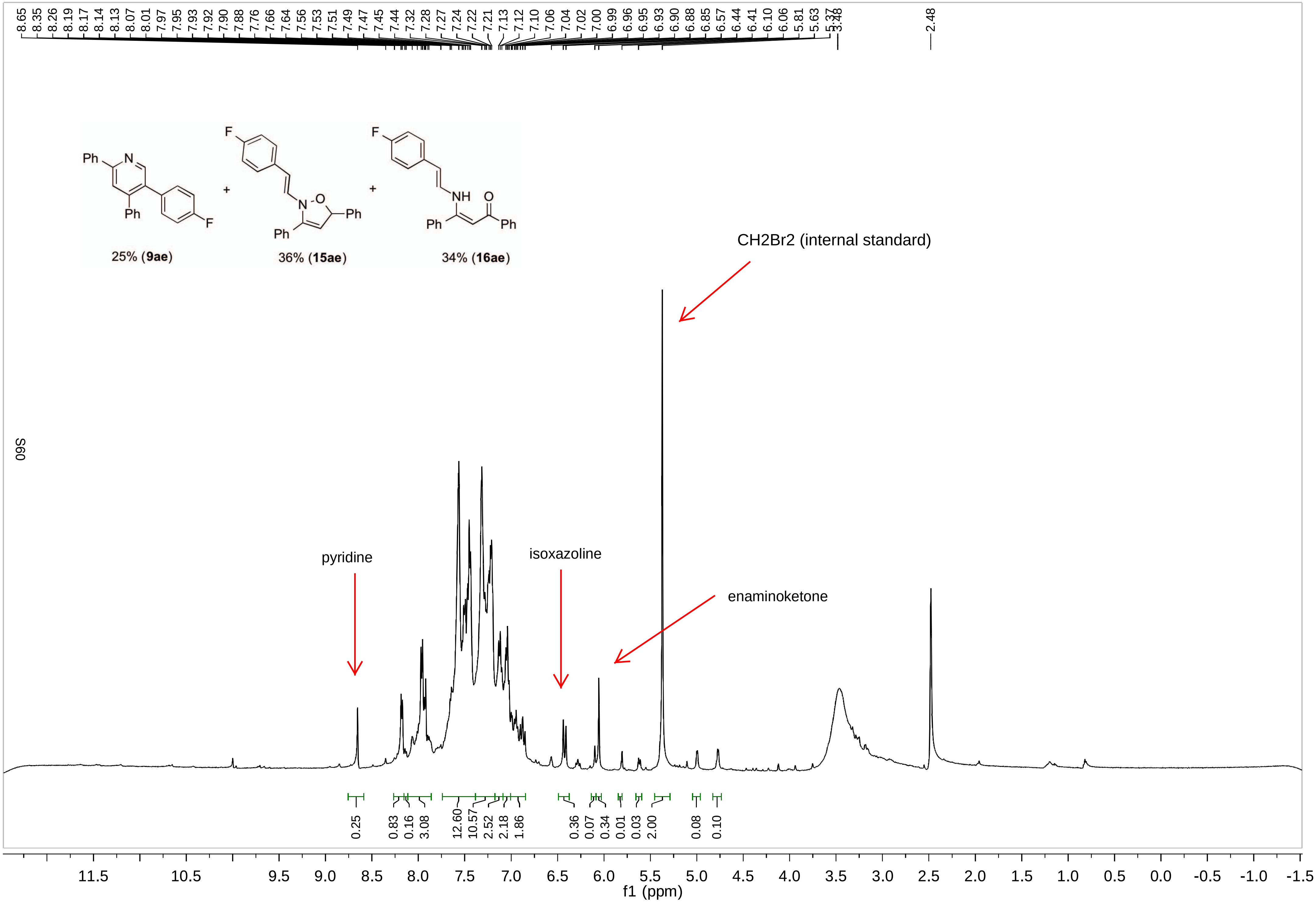
25% (9ae)
36% (15ae)
34% (16ae)
CH2Br2 (internal standard)
pyridine
isoxazoline
enaminoketone
f1 (ppm)

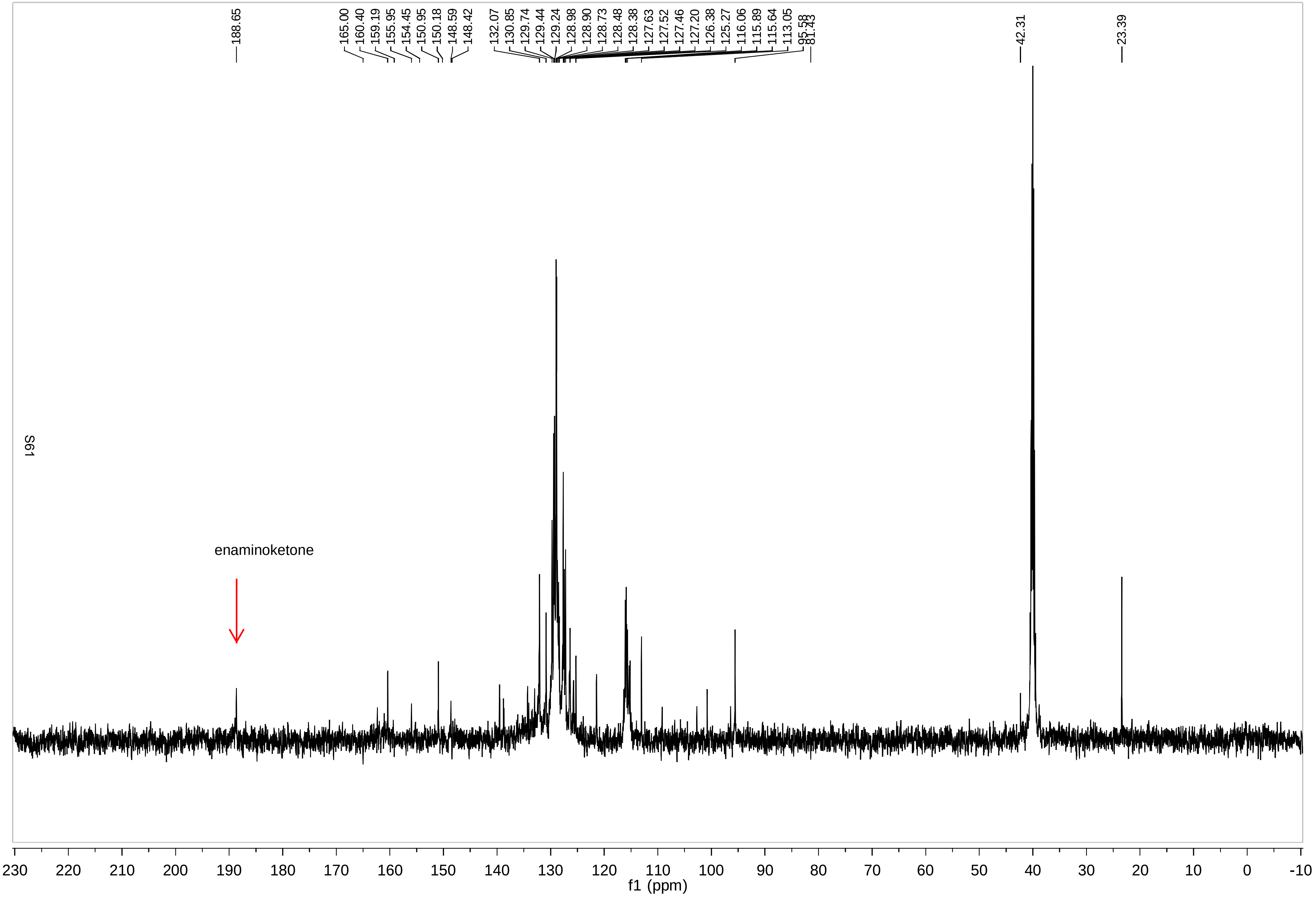
188.65
165.00
160.40
159.19
155.95
154.45
150.95
150.18
148.59
148.42
132.07
130.85
129.74
129.44
129.24
128.98
128.90
128.73
128.48
128.38
127.63
127.52
127.46
127.20
126.38
125.27
116.06
115.89
115.64
113.05
95.58
81.43
42.31
23.39
enaminoketone
230 220 210 200 190 180 170 160 150 140 130 120 110 100 90 80 70 60 50 40 30 20 10 0 -10
f1 (ppm)
